# Supplementary material for: The Spc105/Kre28 complex promotes mitotic error correction by outer kinetochore recruitment of Ipl1/Sli15
Source: EMBO J. 2025 Apr 25;44(12):3492–520. doi: 10.1038/s44318-025-00437-w (PMC12170873; doi:10.1038/s44318-025-00437-w)
Supplement: Supplementary file 1 — Appendix [file 44318_2025_437_MOESM1_ESM.pdf]

Appendix for

The Spc105/Kre28 complex promotes mitotic error correction by outer kinetochore recruitment of Ipl1/Sli15

Alexander Dudziak[1], Richard Pleuger[1], Jasmin Schmidt[1], Frederik Hamm[1], Sharvari Tendulkar[1], Karolin Jänen[1], Ingrid R. Vetter[2], Sylvia Singh[3], Josef Fischböck[3], Franz Herzog[3,4] and Stefan Westermann[1]

[1]Department of Molecular Genetics I, Faculty of Biology, Center of Medical Biotechnology, University of Duisburg-Essen, Universitätsstrasse 5, 45117 Essen, Germany

[2]Department of Mechanistic Cell Biology, Max-Planck-Institute of Molecular Physiology, Otto-Hahn-Straße 11, 44227 Dortmund, Germany

[3]Gene Center Munich and Department of Biochemistry, Ludwig-Maximilians-Universität München, Feodor-Lynen-Str. 25, 81377 Munich, Germany

[4]Institute Krems Bioanalytics, IMC University of Applied Sciences, Krems, Piaristengasse 1, A-3500 Krems, Austria

Table of contents

Appendix Figure S1……………………………………………………………p2

Appendix Figure S2……………………………………………………………p3

Appendix Figure S3……………………………………………………………p4

Appendix Table S1 – Yeast strains used in the study……………………….p5

Appendix Figure S1

Dudziak et al., 2025

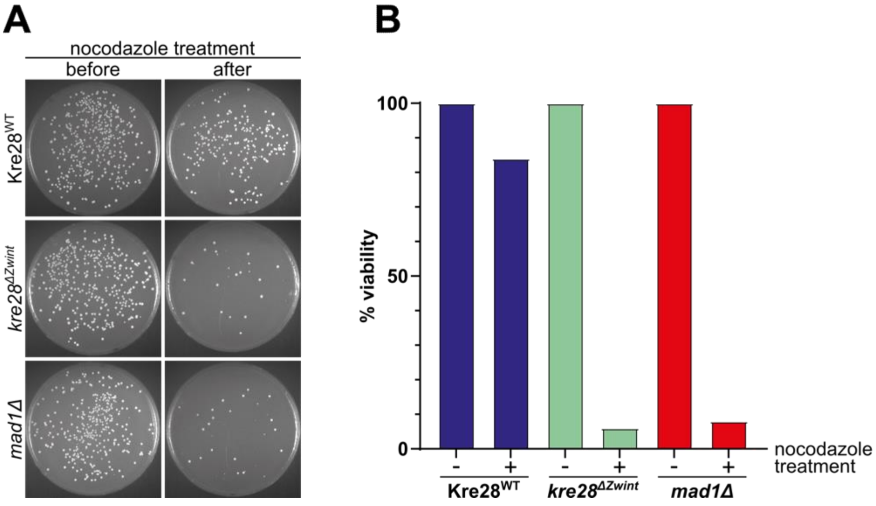

Appendix Figure S1 – Supplement: Viability assay after nocodazole treatment

Kre28^{WT}, *kre28*$^{\Delta Zwint}$ and *mad1*Δ cells were treated with nocodazole for 2 hours. Before and after treatment, a defined number of cells was plated on YEPD plates and incubated at 30 °C. After two days, the number of colonies was counted. A: Images of the plates. B: Quantification of colony formation in the right. For each strain, the number of colonies of the untreated samples was defined as 100 %.

Appendix Figure S2

Dudziak et al., 2025

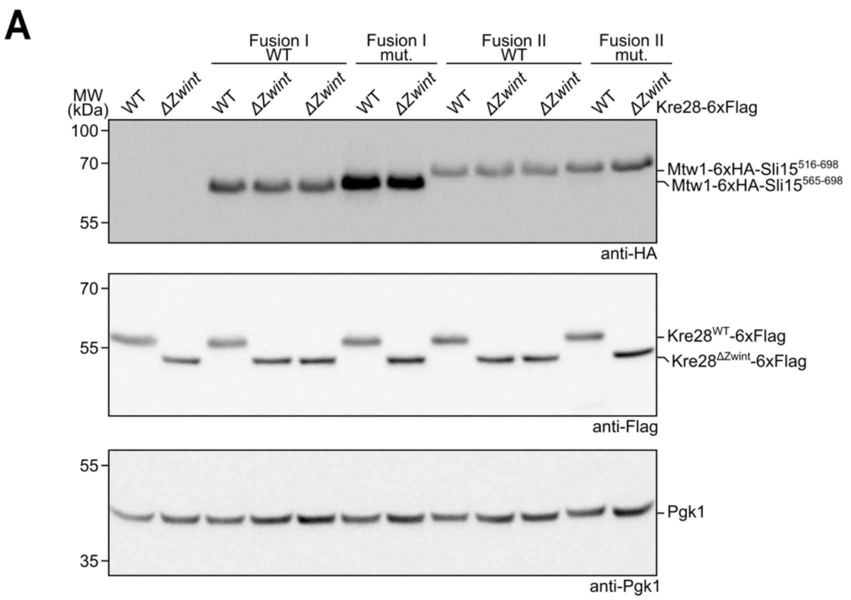

Appendix Figure S2 – Supplement: Western blot analysis of different Mtw1-Sli15 fusion proteins. Western blot analysis comparing the expression of Mtw1-6xHA-Sli15 fusion proteins (shorter Fusion I and longer Fusion II) in wild-type form or in mutant form preventing Ipl1 binding. The respective strains were used for serial dilution assays shown in Figure 6B. Pgk1 served as loading control.

Appendix Figure S3

Dudziak et al., 2025

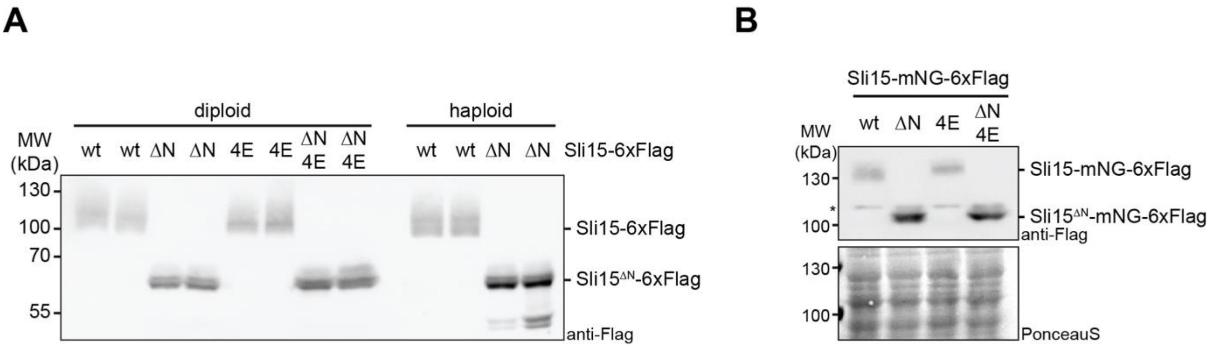

Appendix Figure S3. **A**. Western blot analysis of Sli15 replacement constructs in diploid and haploid cells. Yeast extracts were generated from strains with the indicated genotype and blotted against the Flag tag. Note that 4E mutants were not recovered in haploid cells. **B:** Western blot analysis of Sli15-mNeonGreen constructs in diploid cells.

Appendix Table S1: Yeast strains used in this study

Name	Genotype	MAT	His	Ura	Lys	Ade	Leu	Figure
ADY560	Kre28/Kre28^{WT}-6xFlag::Ura3	a/α	*his3Δ200/ his3Δ200*		Lys2/*lys2-801*	Ade2/*ade2-1*	*leu2-3,112/leu2-3,112*	Fig 1
ADY561	Kre28/*kre28*$^{\Delta Zwint}$-6xFlag::Ura3	a/α	*his3Δ200/ his3Δ200*		Lys2/*lys2-801*	Ade2/*ade2-1*	*leu2-3,112/leu2-3,112*	Fig 1
ADY784	Kre28/*kre28*$^{\Delta RWD}$-6xFlag::Ura3	a/α	*his3Δ200/ his3Δ200*		Lys2/*lys2-801*	Ade2/*ade2-1*	*leu2-3,112/leu2-3,112*	Fig 1
ADY785	Kre28/*kre28*$^{\Delta Zwint\ \Delta RWD}$-6xFlag::Ura3	a/α	*his3Δ200/ his3Δ200*		Lys2/*lys2-801*	Ade2/*ade2-1*	*leu2-3,112/leu2-3,112*	Fig 1
DDY904	wildtype strain background	α	*his3Δ200*		*lys2-801*		*leu2-3,112*	Fig 1
DDY1502	*mad1*Δ::His3	a		*ura3-52*		*ade2-101*	*leu2-3,112*	Fig 2
ADY565	Kre28^{WT}-6xFlag::Ura3	α	*his3Δ200*		*lys2-801*		*leu2-3,112*	Fig 1, 6, Fig1 S2
ADY567	*kre28*$^{\Delta Zwint}$-6xFlag::Ura3	α	*his3Δ200*		*lys2-801*		*leu2-3,112*	Fig 1, 6, Fig1 S2
ADY789	*kre28*$^{\Delta RWD}$-6xFlag::Ura3	α	*his3Δ200*		*lys2-801*		*leu2-3,112*	Fig 1
JPY25.1	*kre28*Δ::His3, *ura3-52*::Kre28^{WT}-6xFlag::Ura3	a				*ade2-1*	*leu2-3,112*	Fig EV1
FHY21a	*kre28*Δ::His3, *ura3-52*::*kre28*$^{\Delta Zwint}$-6xFlag::Ura3	a				*ade2-1*	*leu2-3,112*	Fig EV1
JPY21.3	*kre28*Δ::His3, *ura3-52*::*kre28*$^{\Delta 91\text{-}102}$-6xFlag::Ura3	a				*ade2-1*	*leu2-3,112*	Fig EV1
JPY21.4	*kre28*Δ::His3, *ura3-52*::*kre28*$^{\Delta 91\text{-}102}$-6xFlag::Ura3	a				*ade2-1*	*leu2-3,112*	Fig EV1
JPY22.2	*kre28*Δ::His3, *ura3-52*::*kre28*$^{\Delta 77\text{-}90}$-6xFlag::Ura3	α				*ade2-1*	*leu2-3,112*	Fig EV1
JPY22.4	*kre28*Δ::His3, *ura3-52*::*kre28*$^{\Delta 77\text{-}90}$-6xFlag::Ura3	a					*leu2-3,112*	Fig EV1
JPY23.3	*kre28*Δ::His3, *ura3-52*::*kre28*$^{\Delta 103\text{-}113}$-6xFlag::Ura3	α				*ade2-1*	*leu2-3,112*	Fig EV1
JPY23.4	*kre28*Δ::His3, *ura3-52*::*kre28*$^{\Delta 103\text{-}113}$-6xFlag::Ura3	a			*lys2-801*	*ade2-1*	*leu2-3,112*	Fig EV1
FHY19b	*kre28*Δ::His3, *ura3-52*::Kre28^{WT}-6xFlag::Ura3	α			*lys2-801*		*leu2-3,112*	Fig EV1
FHY22b	*kre28*Δ::His3, *ura3-52*::*kre28*$^{\Delta Zwint}$-6xFlag::Ura3	α			*lys2-801*		*leu2-3,112*	Fig EV1
ADY623.1	*kre28*Δ::His3, *ura3-52*::*kre28*^{4A}-6xFlag::Ura3 (L95A, K96A, Y99A, E101A)	α					*leu2-3,112*	Fig EV1
ADY623.2	*kre28*Δ::His3, *ura3-52*::*kre28*^{4A}-6xFlag::Ura3 (L95A, K96A, Y99A, E101A)	α					*leu2-3,112*	Fig EV1
ADY625.1	*kre28*Δ::His3, *ura3-52*::*kre28*^{3A}-6xFlag::Ura3 (E103A, L105A, D106A)	α					*leu2-3,112*	Fig EV1
ADY625.2	*kre28*Δ::His3, *ura3-52*::*kre28*^{3A}-6xFlag::Ura3 (E103A, L105A, D106A)	α			*lys2-801*		*leu2-3,112*	Fig EV1
ADY627.1	*kre28*Δ::His3, *ura3-52*::*kre28*^{6A}-6xFlag::Ura3 (F108A, F109A, R110A, F111A, T112A, L113A)	α					*leu2-3,112*	Fig EV1
ADY627.2	*kre28*Δ::His3, *ura3-52*::*kre28*^{6A}-6xFlag::Ura3 (F108A, F109A, R110A, F111A, T112A, L113A)	α				*ade2-1*	*leu2-3,112*	Fig EV1
ADY573	Kre28^{WT}-6xFlag::Ura3, Pds1-18xMyc::Leu2	a	*his3Δ200*			*ade2-1*		Fig 2
ADY574	*kre28*$^{\Delta Zwint}$-6xFlag::Ura3, Pds1-18xMyc::Leu2	a	*his3Δ200*			*ade2-1*		Fig 2
ADY539	*mad1*Δ::His3, Pds1-18xMyc::Leu2	a		*ura3-52*		*ade2-1*		Fig 2
DDY902	wildtype strain background	a	*his3Δ200*	*ura3-52*		*ade2-1*	*leu2-3,112*	Fig 2
ADY564	Kre28^{WT}-6xFlag::Ura3	a	*his3Δ200*			*ade2-1*	*leu2-3,112*	Fig 2
ADY566	*kre28*$^{\Delta Zwint}$-6xFlag::Ura3	a	*his3Δ200*			*ade2-1*	*leu2-3,112*	Fig 2
ADY581.1	Kre28^{WT}-6xFlag::Ura3, *mad1*Δ::His3	a				*ade2-1*		Fig 2

ADY581.2	Kre28^{WT}-6xFlag::Ura3, *mad1*Δ::His3	a			*lys2-801*	*ade2-1*		Fig 2
ADY583.1/2	*kre28*$^{\Delta Zwint}$-6xFlag::Ura3, *mad1*Δ::His3	a				*ade2-1*		Fig 2
ADY562	Kre28^{WT}-6xFlag::Ura3, *cdc15-2*::Leu2, *his3Δ200*::pCup1-LacI-GFP::His3, CEN XII-prox.::LacO::Leu2, Spc42-mCherry::KanMx6	a				*ade2-1*		Fig 2, 4
ADY563	*kre28*$^{\Delta Zwint}$-6xFlag::Ura3, *cdc15-2*::Leu2, *his3Δ200*::pCup1-LacI-GFP::His3, CEN XII-prox.::LacO::Leu2, Spc42-mCherry::KanMx6	a				*ade2-1*		Fig 2, 4
ADY620	Kre28/Kre28^{WT}-6xFlag::Ura3, Ipl1/*ipl1-1*	a/α	*his3Δ200/ his3Δ200*		Lys2/*lys2-801*	Ade2/*ade2-1*	*leu2-3,112/leu2-3,112*	Fig 2
ADY621	Kre28/*kre28*$^{\Delta Zwint}$-6xFlag::Ura3, Ipl1/*ipl1-1*	a/α	*his3Δ200/ his3Δ200*		Lys2/*lys2-801*	Ade2/*ade2-1*	*leu2-3,112/leu2-3,112*	Fig 2
FHY18a	*kre28*Δ::His3, *ura3-52*::Kre28^{WT}-6xFlag::Ura3	a				*ade2-1*	*leu2-3,112*	Fig 2
SWY2265	*mad1Δ*::KanMx6	α	*his3Δ200*	*ura3-52*	*lys2-801*		*leu2-3,112*	Fig2
ADY661	Kre28^{WT}-6xFlag::Ura3, Mtw1-GFP::KanMx6, Spc42-RFP::His3	a					*leu2-3,112*	Fig 3
ADY663	*kre28*$^{\Delta Zwint}$-6xFlag::Ura3, Mtw1-GFP::KanMx6, Spc42-RFP::His3	a					*leu2-3,112*	Fig 3
ADY674	Kre28^{WT}-6xFlag::Ura3, Nuf2-GFP::KanMx6, Spc42-RFP::His3	a					*leu2-3,112*	Fig 3
ADY668	*kre28*$^{\Delta Zwint}$-6xFlag::Ura3, Nuf2-GFP::KanMx6, Spc42-RFP::His3	a					*leu2-3,112*	Fig 3
ADY638	Kre28^{WT}-6xFlag::Ura3, Spc105-GFP::KanMx6, Spc42-RFP::His3	a					*leu2-3,112*	Fig 3
ADY641	*kre28*$^{\Delta Zwint}$-6xFlag::Ura3, Spc105-GFP::KanMx6, Spc42-RFP::His3	a			*lys2-801*		*leu2-3,112*	Fig 3
ADY791	*kre28*Δ::His3, *ura3-52*::GFP-Kre28^{WT}-6xFlag::Ura3, Spc42-mCherry::KanMx6	α			*lys2-801*		*leu2-3,112*	Fig EV2
ADY793	*kre28*Δ::His3, *ura3-52*::GFP-*kre28*$^{\Delta Zwint}$-6xFlag::Ura3, Spc42-mCherry::KanMx6	α			*lys2-801*		*leu2-3,112*	Fig EV2
ADY639	Kre28^{WT}-6xFlag::Ura3, Spc105-GFP::KanMx6, Spc42-RFP::His3	α			*lys2-801*		*leu2-3,112*	Fig EV2
ADY642	*kre28*$^{\Delta Zwint}$-6xFlag::Ura3, Spc105-GFP::KanMx6, Spc42-RFP::His3	α			*lys2-801*		*leu2-3,112*	Fig EV2
ADY662	Kre28^{WT}-6xFlag::Ura3, Mtw1-GFP::KanMx6, Spc42-RFP::His3	α			*lys2-801*		*leu2-3,112*	Fig EV2
ADY664	*kre28*$^{\Delta Zwint}$-6xFlag::Ura3, Mtw1-GFP::KanMx6, Spc42-RFP::His3	α			*lys2-801*		*leu2-3,112*	Fig EV2
ADY675	Kre28^{WT}-6xFlag::Ura3, Nuf2-GFP::KanMx6, Spc42-RFP::His3	α					*leu2-3,112*	Fig EV2
ADY669	*kre28*$^{\Delta Zwint}$-6xFlag::Ura3, Nuf2-GFP::KanMx6, Spc42-RFP::His3	α					*leu2-3,112*	Fig EV2
ADY790	*kre28*Δ::His3, *ura3-52*::GFP-Kre28^{WT}-6xFlag::Ura3, Spc42-mCherry::KanMx6	a			*lys2-801*		*leu2-3,112*	Fig EV2
ADY792	*kre28*Δ::His3, *ura3-52*::GFP-*kre28*$^{\Delta Zwint}$-6xFlag::Ura3, Spc42-mCherry::KanMx6	a			*lys2-801*		*leu2-3,112*	Fig EV2
ADY712	Kre28^{WT}-GFP::Ura3, Spc42-RFP::His3	a					*leu2-3,112*	Fig EV2
ADY713	Kre28^{WT}-GFP::Ura3, Spc42-RFP::His3	α			*lys2-801*		*leu2-3,112*	Fig EV2
ADY714	*kre28*$^{\Delta Zwint}$-GFP::Ura3, Spc42-RFP::His3	a					*leu2-3,112*	Fig EV2
ADY715	*kre28*$^{\Delta Zwint}$-GFP::Ura3, Spc42-RFP::His3	α			*lys2-801*		*leu2-3,112*	Fig EV2
ADY654	Kre28^{WT}-6xFlag::Ura3, Sgo1-GFP::KanMx6, Spc42-RFP::His3	a					*leu2-3,112*	Fig EV2
ADY656	*kre28*$^{\Delta Zwint}$-6xFlag::Ura3, Sgo1-GFP::KanMx6, Spc42-RFP::His3	a					*leu2-3,112*	Fig EV2
ADY697	Kre28/Kre28^{WT}-6xFlag::Ura3, Sgo1/*sgo1Δ*::natNT2	a/α	*his3Δ200/ his3Δ200*		Lys2/*lys2-801*	Ade2/*ade2-1*	*leu2-3,112/leu2-3,112*	Fig 2
ADY698	Kre28/*kre28*$^{\Delta Zwint}$-6xFlag::Ura3, Sgo1/*sgo1Δ*::natNT2	a/α	*his3Δ200/ his3Δ200*		Lys2/*lys2-801*	Ade2/*ade2-1*	*leu2-3,112/leu2-3,112*	Fig 2

ADY707	Kre28/Kre28^{WT}-6xFlag::Ura3, Sli15/sli15-3	a/α	*his3Δ200*/ *his3Δ200*		Lys2/*lys2-801*	Ade2/*ade2-1*	*leu2-3,112*/*leu2-3,112*	Fig 2
ADY708	Kre28/*kre28*$^{\Delta Zwint}$-6xFlag::Ura3, Sli15/*sli15-3*	a/α	*his3Δ200*/ *his3Δ200*		Lys2/*lys2-801*	Ade2/*ade2-1*	*leu2-3,112*/*leu2-3,112*	Fig 2
ADY618	Kre28/Kre28^{WT}-6xFlag::Ura3, Ndc80/*ndc80-1*	a/α	*his3Δ200*/ *his3Δ200*		Lys2/*lys2-801*	Ade2/*ade2-1*	*leu2-3,112*/*leu2-3,112*	Fig 2
ADY619	Kre28/*kre28*$^{\Delta Zwint}$-6xFlag::Ura3, Ndc80/*ndc80-1*	a/α	*his3Δ200*/ *his3Δ200*		Lys2/*lys2-801*	Ade2/*ade2-1*	*leu2-3,112*/*leu2-3,112*	Fig 2
ADY775	Kre28/Kre28^{WT}-6xFlag::Ura3, Ctf19/Ctf19^{WT}-13xMyc::His3	a/α			Lys2/*lys2-801*	Ade2/*ade2-1*	*leu2-3,112*/*leu2-3,112*	Fig 2
ADY776	Kre28/Kre28^{WT}-6xFlag::Ura3, Ctf19/*ctf19*$^{\Delta C\text{-}RWD\ (270\text{-}369)}$-13xMyc::His3	a/α			Lys2/*lys2-801*	Ade2/*ade2-1*	*leu2-3,112*/*leu2-3,112*	Fig 2
ADY777	Kre28/*kre28*$^{\Delta Zwint}$-6xFlag::Ura3, Ctf19/Ctf19^{WT}-13xMyc::His3	a/α			Lys2/*lys2-801*	Ade2/*ade2-1*	*leu2-3,112*/*leu2-3,112*	Fig 2
ADY778	Kre28/*kre28*$^{\Delta Zwint}$-6xFlag::Ura3, Ctf19/*ctf19*$^{\Delta C\text{-}RWD\ (270\text{-}369)}$-13xMyc::His3	a/α			Lys2/*lys2-801*	Ade2/*ade2-1*	*leu2-3,112*/*leu2-3,112*	Fig 2
ADY616	Kre28/Kre28^{WT}-6xFlag::Ura3, Dam1/*dam1-1*::KanMx6	a/α	*his3Δ200*/ *his3Δ200*		Lys2/*lys2-801*	Ade2/*ade2-1*	*leu2-3,112*/*leu2-3,112*	Fig 2
ADY617	Kre28/*kre28*$^{\Delta Zwint}$-6xFlag::Ura3, Dam1/*dam1-1*::KanMx6	a/α	*his3Δ200*/ *his3Δ200*		Lys2/*lys2-801*	Ade2/*ade2-1*	*leu2-3,112*/*leu2-3,112*	Fig 2
ADY315	*duo1*$^{\Delta SxIP}$::Leu2	a	*his3Δ200*	*ura3-52*		*ade2-1*		Fig 2
ADY577.1/2	Kre28^{WT}-6xFlag::Ura3, *duo1*$^{\Delta SxIP}$	a	*his3Δ200*			*ade2-1*		Fig 2
ADY579.1	Kre28$^{\Delta Zwint}$-6xFlag::Ura3, *duo1*$^{\Delta SxIP}$	a	*his3Δ200*			*ade2-1*		Fig 2
ADY579.2	Kre28$^{\Delta Zwint}$-6xFlag::Ura3, *duo1*$^{\Delta SxIP}$	a	*his3Δ200*		*lys2-801*	*ade2-1*		Fig 2
SWY389A	*cnn1Δ*::His3	a		*ura3-52*		*ade2-1*	*leu2-3,112*	Fig 2
ADY685	Kre28^{WT}-6xFlag::Ura3, *cnn1Δ*::His3	a				*ade2-1*	*leu2-3,112*	Fig 2
ADY686	Kre28^{WT}-6xFlag::Ura3, *cnn1Δ*::His3	α			*lys2-801*		*leu2-3,112*	Fig 2
ADY687	*kre28*$^{\Delta SxIP}$-6xFlag::Ura3, *cnn1Δ*::His3	a				*ade2-1*	*leu2-3,112*	Fig 2
ADY688	*kre28*$^{\Delta SxIP}$-6xFlag::Ura3, *cnn1Δ*::His3	α			*lys2-801*		*leu2-3,112*	Fig 2
ADY768	Kre28^{WT}-6xFlag::Ura3, *lys2-801*::Mtw1-6xHA-Sli15$^{565\text{-}698\ (WT)}$::Lys2	α	*his3Δ200*				*leu2-3,112*	Fig 4
ADY769	*kre28*$^{\Delta Zwint}$-6xFlag::Ura3, *lys2-801*::Mtw1-6xHA-Sli15$^{565\text{-}698\ (WT)}$::Lys2	α	*his3Δ200*				*leu2-3,112*	Fig 4
ADY802	Kre28^{WT}-6xFlag::Ura3, *lys2-801*::Mtw1-6xHA-Sli15$^{516\text{-}698\ (WT)}$::Lys2	α	*his3Δ200*				*leu2-3,112*	Fig 4
ADY803	*kre28*$^{\Delta Zwint}$-6xFlag::Ura3, *lys2-801*::Mtw1-6xHA-Sli15$^{516\text{-}698\ (WT)}$::Lys2	α	*his3Δ200*				*leu2-3,112*	Fig 4
ADY794	Kre28^{WT}-6xFlag::Ura3, *lys2-801*::Mtw1-6xHA-Sli15$^{565\text{-}698\ (W646G,\ F680A)}$::Lys2	α	*his3Δ200*				*leu2-3,112*	Fig 4
ADY795	*kre28*$^{\Delta Zwint}$-6xFlag::Ura3, *lys2-801*::Mtw1-6xHA-Sli15$^{565\text{-}698\ (W646G,\ F680A)}$::Lys2	α	*his3Δ200*				*leu2-3,112*	Fig 4
ADY840	Kre28^{WT}-6xFlag::Ura3, *lys2-801*::Mtw1-6xHA-Sli15$^{516\text{-}698\ (W646G,\ F680A)}$::Lys2	α	*his3Δ200*				*leu2-3,112*	Fig 4
ADY841	*kre28*$^{\Delta Zwint}$-6xFlag::Ura3, *lys2-801*::Mtw1-6xHA-Sli15$^{516\text{-}698\ (W646G,\ F680A)}$::Lys2	α	*his3Δ200*				*leu2-3,112*	Fig 4
ADY836	Kre28^{WT}-6xFlag::Ura3, *cdc15-2*::Leu2, *his3Δ200*::pCup1-LacI-GFP::His3, CEN XII-prox.::LacO::Leu2, Spc42-mCherry::KanMx6, *lys2-801*::Mtw1-6xHA-Sli15$^{565\text{-}698\ (WT)}$::natNT2::Lys2	α				*ade2-1*		Fig 4
ADY837	*kre28*$^{\Delta Zwint}$-6xFlag::Ura3, *cdc15-2*::Leu2, *his3Δ200*::pCup1-LacI-GFP::His3, CEN XII-prox.::LacO::Leu2, Spc42-mCherry::KanMx6, *lys2-801*::Mtw1-6xHA-Sli15$^{565\text{-}698\ (WT)}$::natNT2::Lys2	α				*ade2-1*		Fig 4

ADY838	Kre28^{WT}-6xFlag::Ura3, *cdc15-2*::Leu2, *his3Δ200*::pCup1-LacI-GFP::His3, CEN XII-prox.::LacO::Leu2, Spc42-mCherry::KanMx6, *lys2-801*::Mtw1-6xHA-Sli15$^{516\text{-}698\ (WT)}$::natNT2::Lys2	α				*ade2-1*		Fig 4
ADY839	*kre28$^{\Delta Zwint}$*-6xFlag::Ura3, *cdc15-2*::Leu2, *his3Δ200*::pCup1-LacI-GFP::His3, CEN XII-prox.::LacO::Leu2, Spc42-mCherry::KanMx6, *lys2-801*::Mtw1-6xHA-Sli15$^{516\text{-}698\ (WT)}$::natNT2::Lys2	α				*ade2-1*		Fig 4
ADY816	Kre28 WT-6xFlag, Spc105-TurboID-3xMyc::KanMx6	a	*his3Δ200*			*ade2-1*	*leu2-3,112*	Fig 5
ADY828	Spc105-TurboID-3xMyc::KanMx6, Sli15 WT-6xFlag::His3	a		*ura3-52*		*ade2-1*	*leu2-3,112*	Fig 5
STY327	Cin8-6xFlag::URA3	a	*his3Δ200*				*leu2-3,112*	Fig 5
STY328	Cin8-6xFlag::URA3, Spc105-TurboID-3xMyc::KanMx6	a	*his3Δ200*				*leu2-3,112*	Fig 5
ADY865	sli15::Sli15-6xFlag::His3	a						Fig 7
ADY866	sli15::Sli15(delta2-230)-6xFlag::His3	a						Fig 7
ADY873	Mtw1/Mtw1-mCherry::KanMx6, Sli15/Sli15-mNeonGreen-6xFlag	a/α						Fig 7
ADY874	Mtw1/Mtw1-mCherry::KanMx6, Sli15/Sli15(delta2-230)-mNeonGreen-6xFlag	a/α						Fig 7
ADY875	Mtw1/Mtw1-mCherry::KanMx6, Sli15/Sli15(R231E R232E L239E K242E)-mNeonGreen-6xFlag	a/α						Fig 7
ADY876	Mtw1/Mtw1-mCherry::KanMx6, Sli15/Sli15(delta2-230, R231E R232E L239E K242E)-mNeonGreen-6xFlag	a/α						Fig 7
ADY877	Sli15-aid*-9xmyc::KanMX6 ura3-52::pTEF1-OsTIR1_tADH1::URA3	a	*his3Δ200*					Fig EV5
ADY878	Sli15-aid::KanMX with Sli15-wt-6xFlag::LEU2	a	*his3Δ200*					Fig EV5
ADY879	Sli15-aid::KanMX with Sli15-deltaN-6xFlag::LEU	a	*his3Δ200*					Fig EV5
ADY880	Sli15-aid::KanMX with Sli15-(R231E R232E L239E K242E)-6xFlag::LEU2	a	*his3Δ200*					Fig EV5
ADY881	Sli15-aid::KanMX with Sli15(delta2-230, R231E R232E L239E K242E)-6xFlag	a	*his3Δ200*					Fig EV5
ADY882	Sli15-aid::KanMX with Sli15-2E (R231E R1232E)-6xFlag::LEU2	a	*his3Δ200*					Fig EV5
ADY883	Sli15-aid::KanMX with Sli15-1E(K242E)-6xFlag::LEU2	a	*his3Δ200*					Fig EV5
ADY884	Spc105-FRB::KanMX, ura3-52::Spc105-wt::URA3	a						Fig EV5
ADY885	Spc105-FRB::KanMX, ura3-52::Spc105 E578K E596K::URA3	a						Fig EV5
ADY886	Spc105-FRB::KanMX, ra3-52::Spc105 E510K E578K E596K::URA3	a						Fig EV5
