# Supplementary figures and images for: The Spc105/Kre28 complex promotes mitotic error correction by outer kinetochore recruitment of Ipl1/Sli15
Source: EMBO J. 2025 Apr 25;44(12):3492–520. doi: 10.1038/s44318-025-00437-w (PMC12170873; doi:10.1038/s44318-025-00437-w)

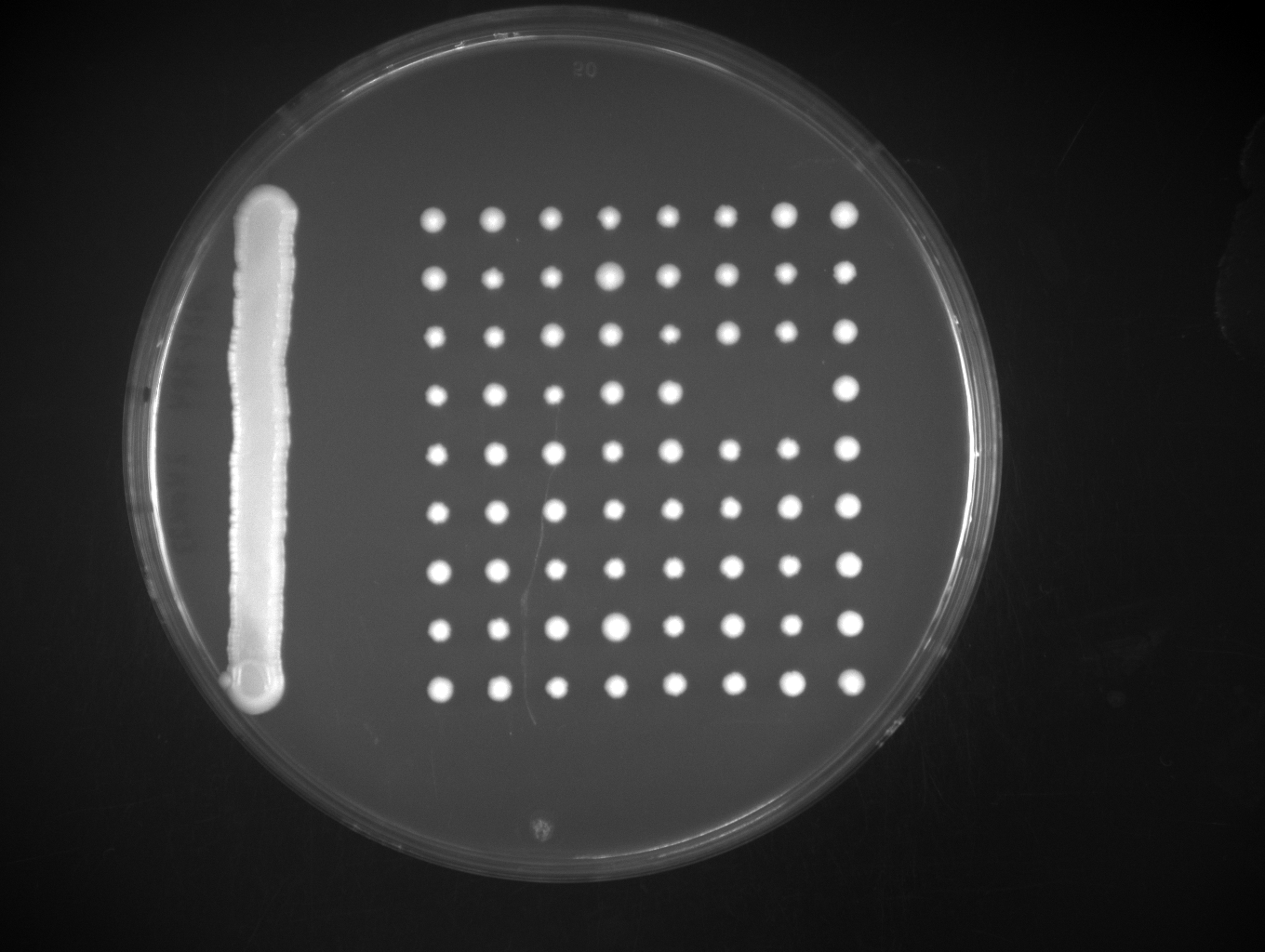

Supplement: Supplementary file 3 — Source data Fig. 1 [file 44318_2025_437_MOESM3_ESM.zip › Figure1/1C/WholeMount_Dissection_Kre28_deltaRWD.tif]

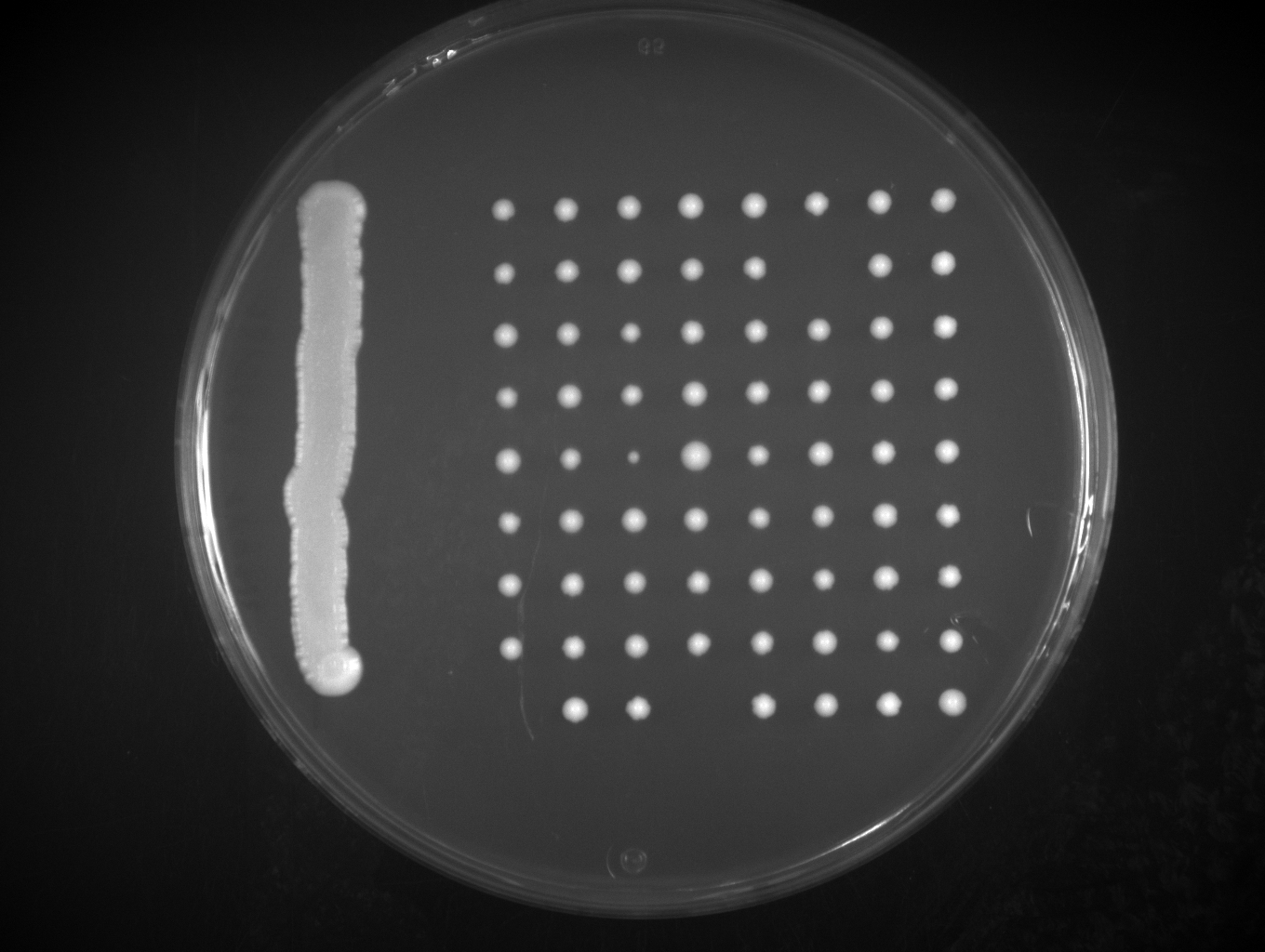

Supplement: Supplementary file 3 — Source data Fig. 1 [file 44318_2025_437_MOESM3_ESM.zip › Figure1/1C/WholeMount_Dissection_Kre28_DeltaZwint.tif]

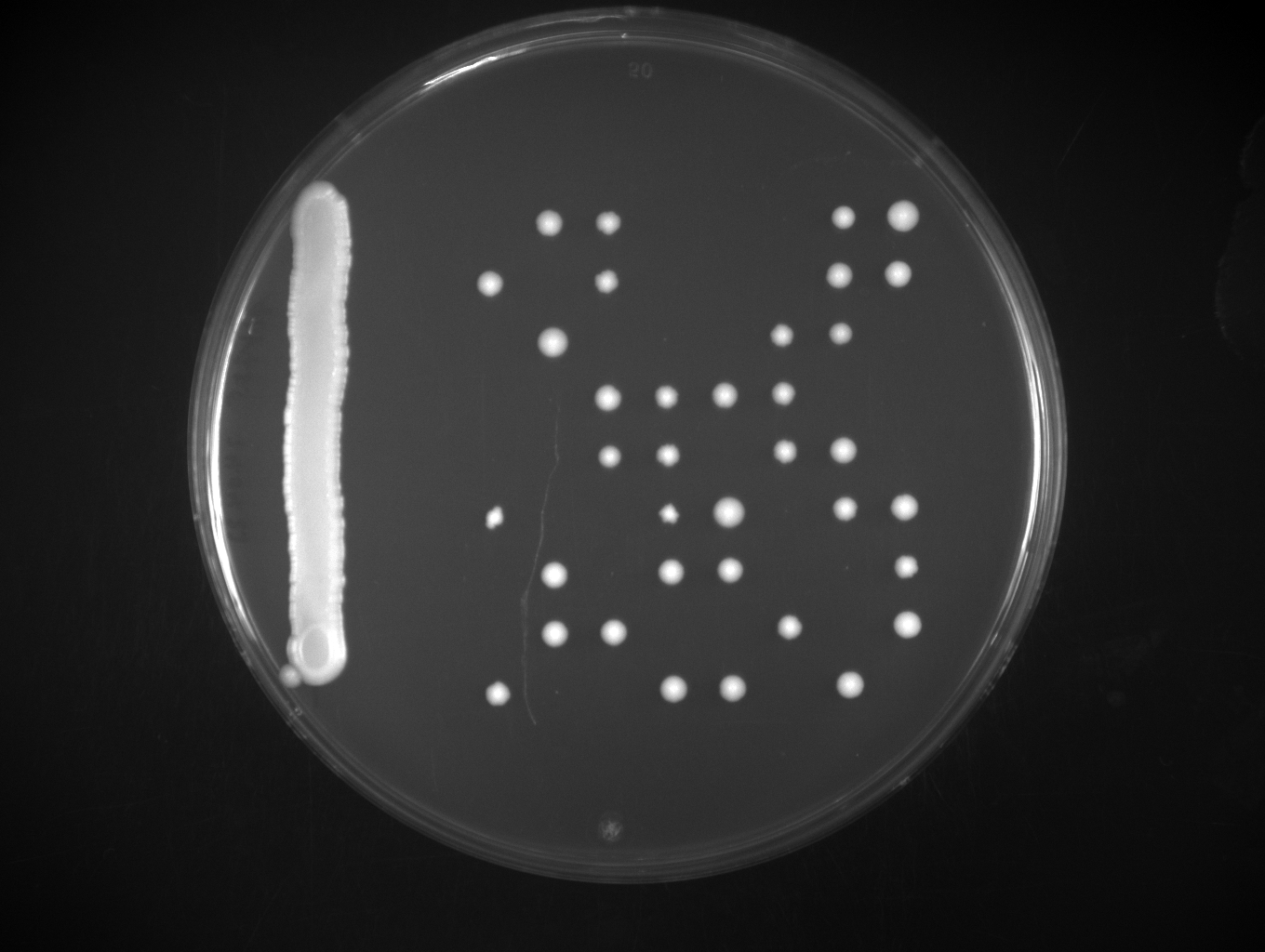

Supplement: Supplementary file 3 — Source data Fig. 1 [file 44318_2025_437_MOESM3_ESM.zip › Figure1/1C/WholeMount_Dissection_Kre28_deltaZwintRWD.tif]

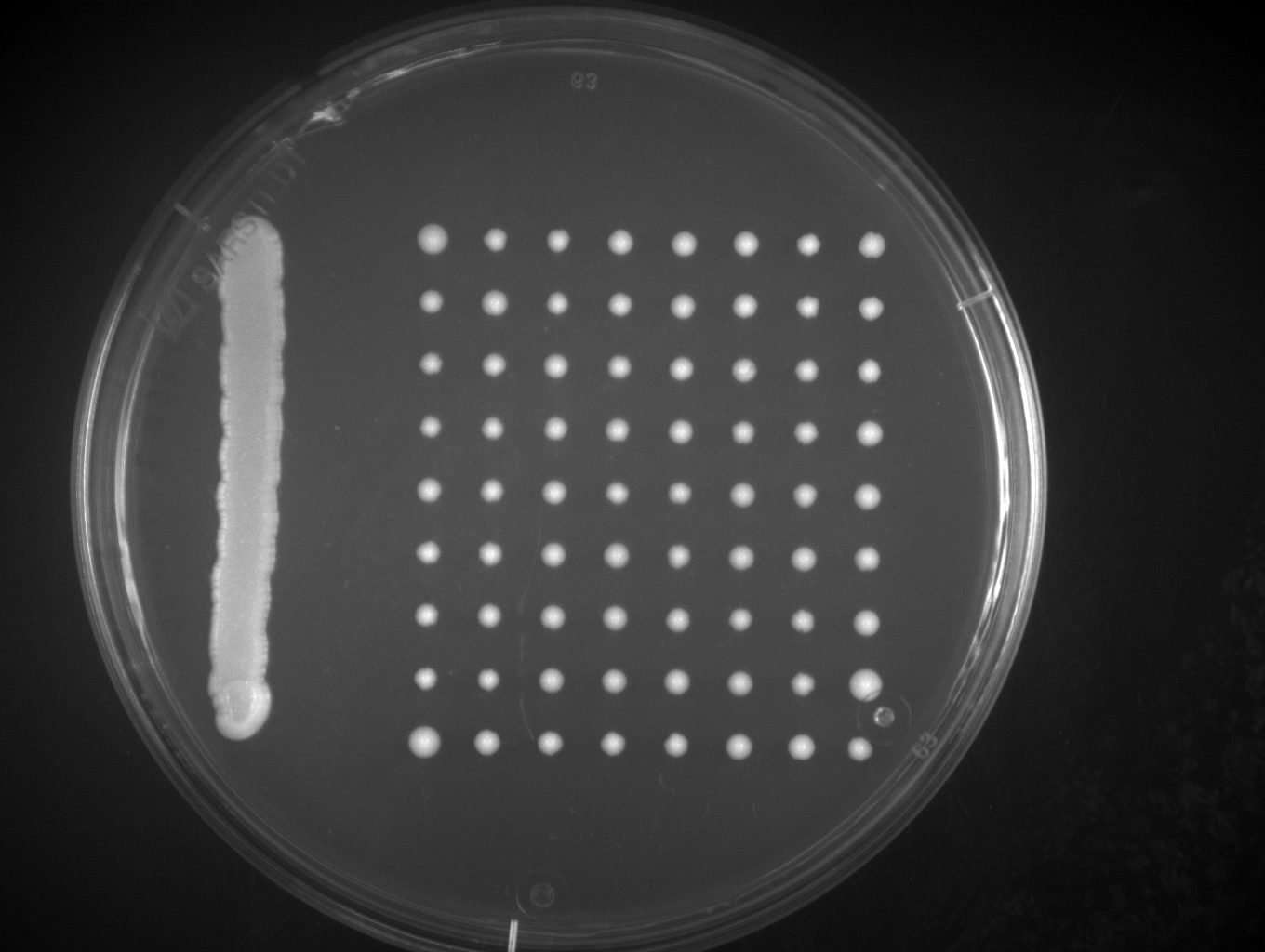

Supplement: Supplementary file 3 — Source data Fig. 1 [file 44318_2025_437_MOESM3_ESM.zip › Figure1/1C/WholeMount_Dissection_Kre28_wt.tif]

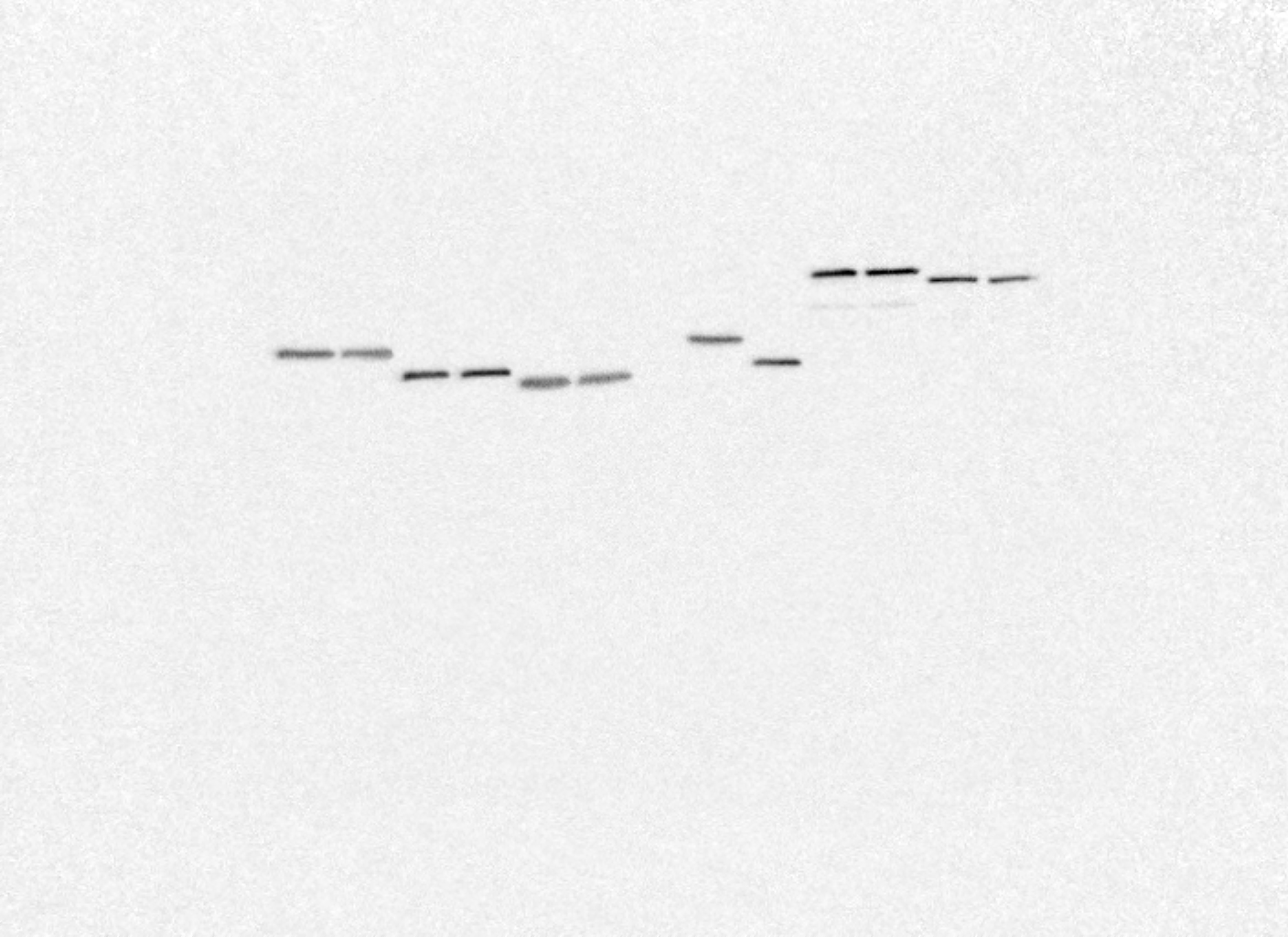

Supplement: Supplementary file 3 — Source data Fig. 1 [file 44318_2025_437_MOESM3_ESM.zip › Figure1/1D/Blot_antiFlag.tif]

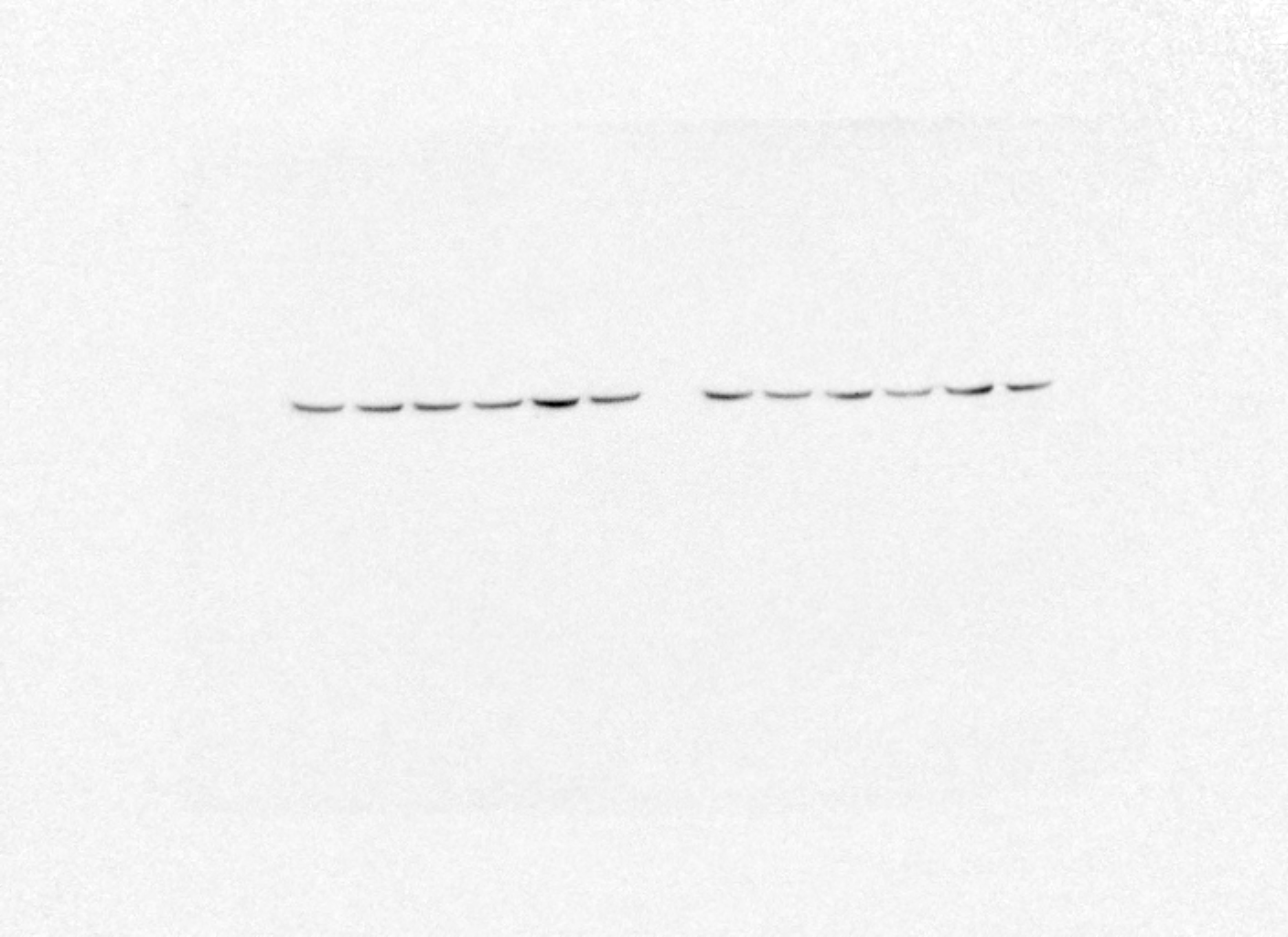

Supplement: Supplementary file 3 — Source data Fig. 1 [file 44318_2025_437_MOESM3_ESM.zip › Figure1/1D/Blot_antiPGK1.tif]

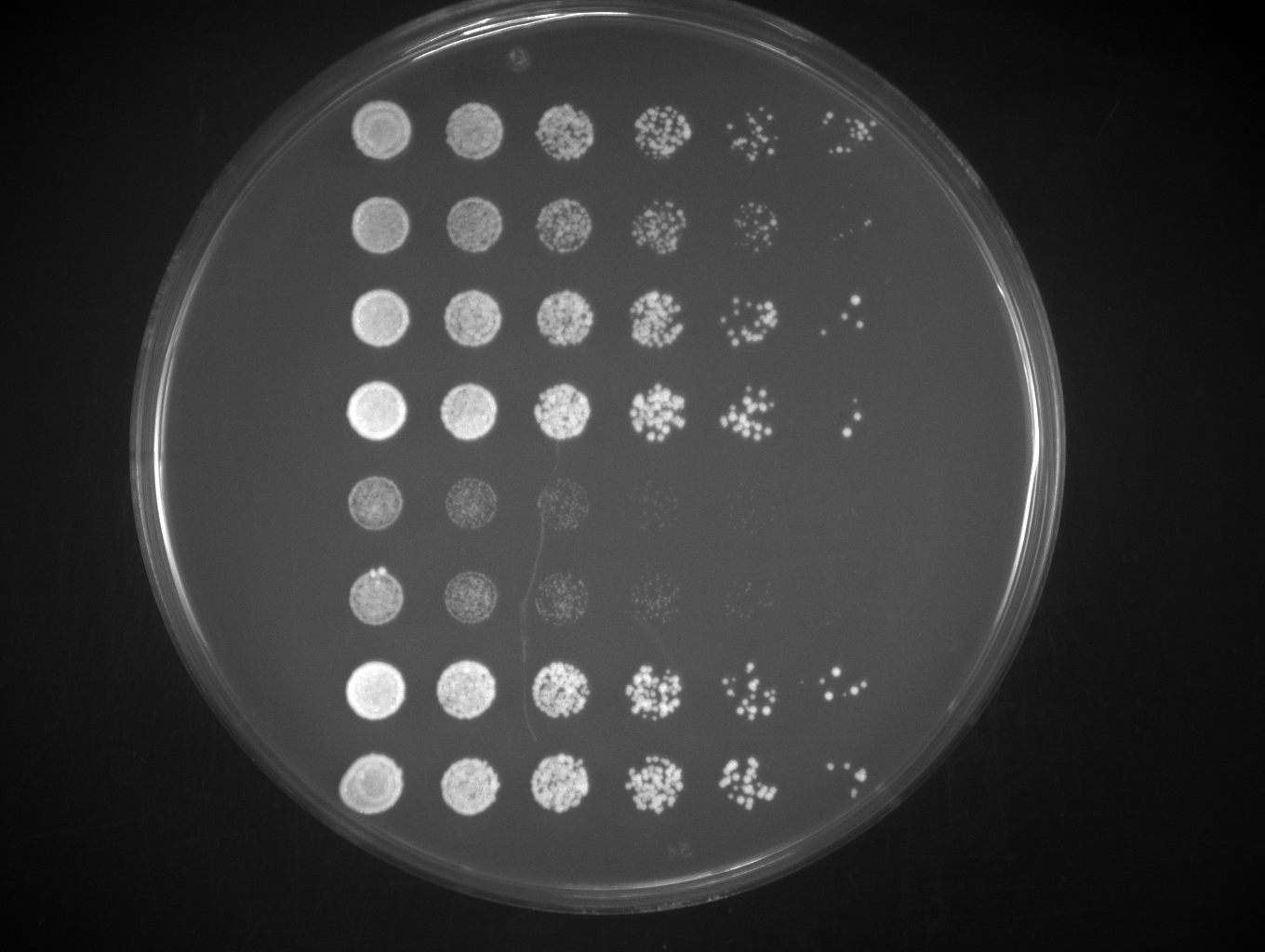

Supplement: Supplementary file 3 — Source data Fig. 1 [file 44318_2025_437_MOESM3_ESM.zip › Figure1/1E/16 °C plate.tif]

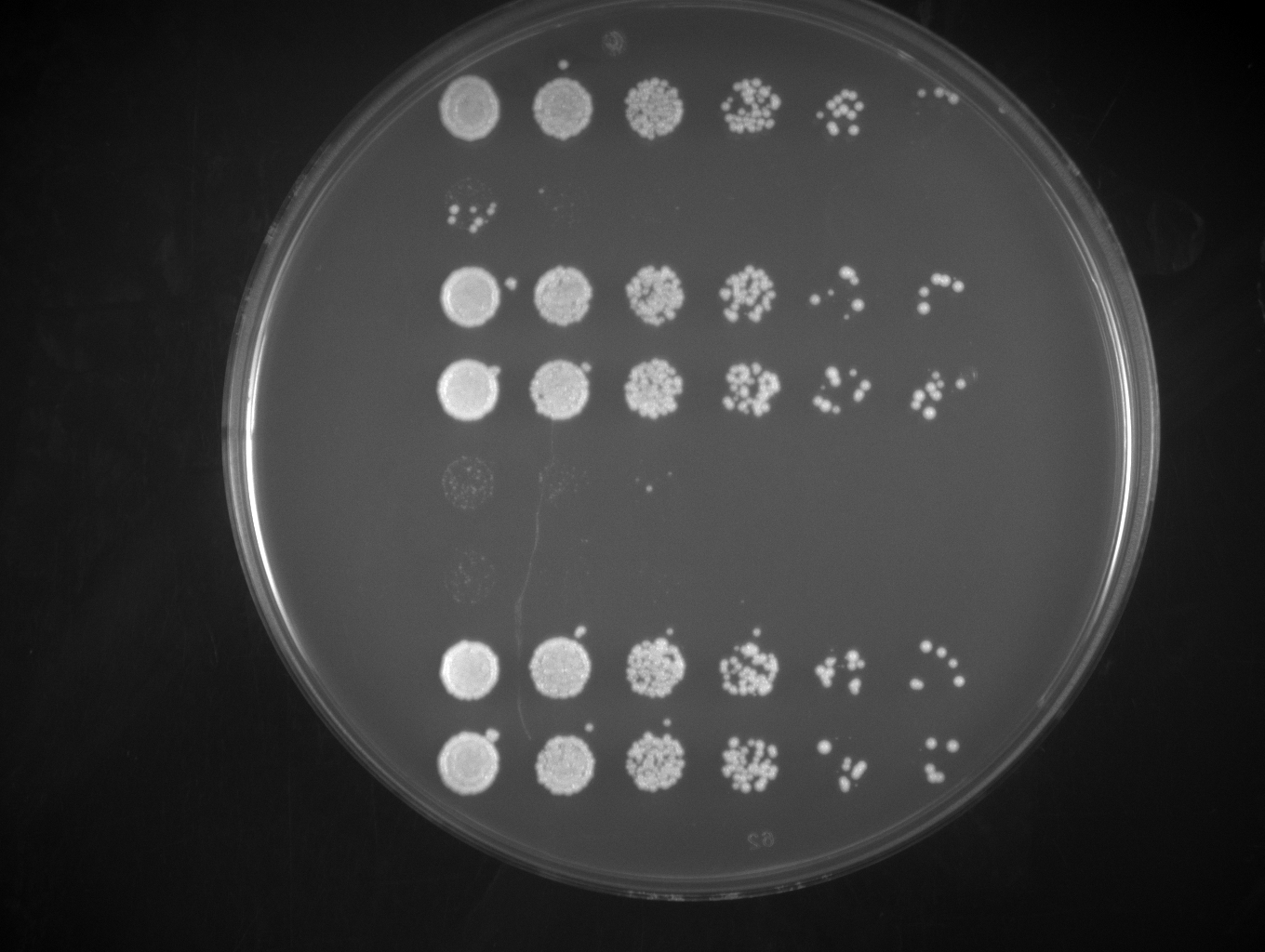

Supplement: Supplementary file 3 — Source data Fig. 1 [file 44318_2025_437_MOESM3_ESM.zip › Figure1/1E/30 °C Benomyl plate.tif]

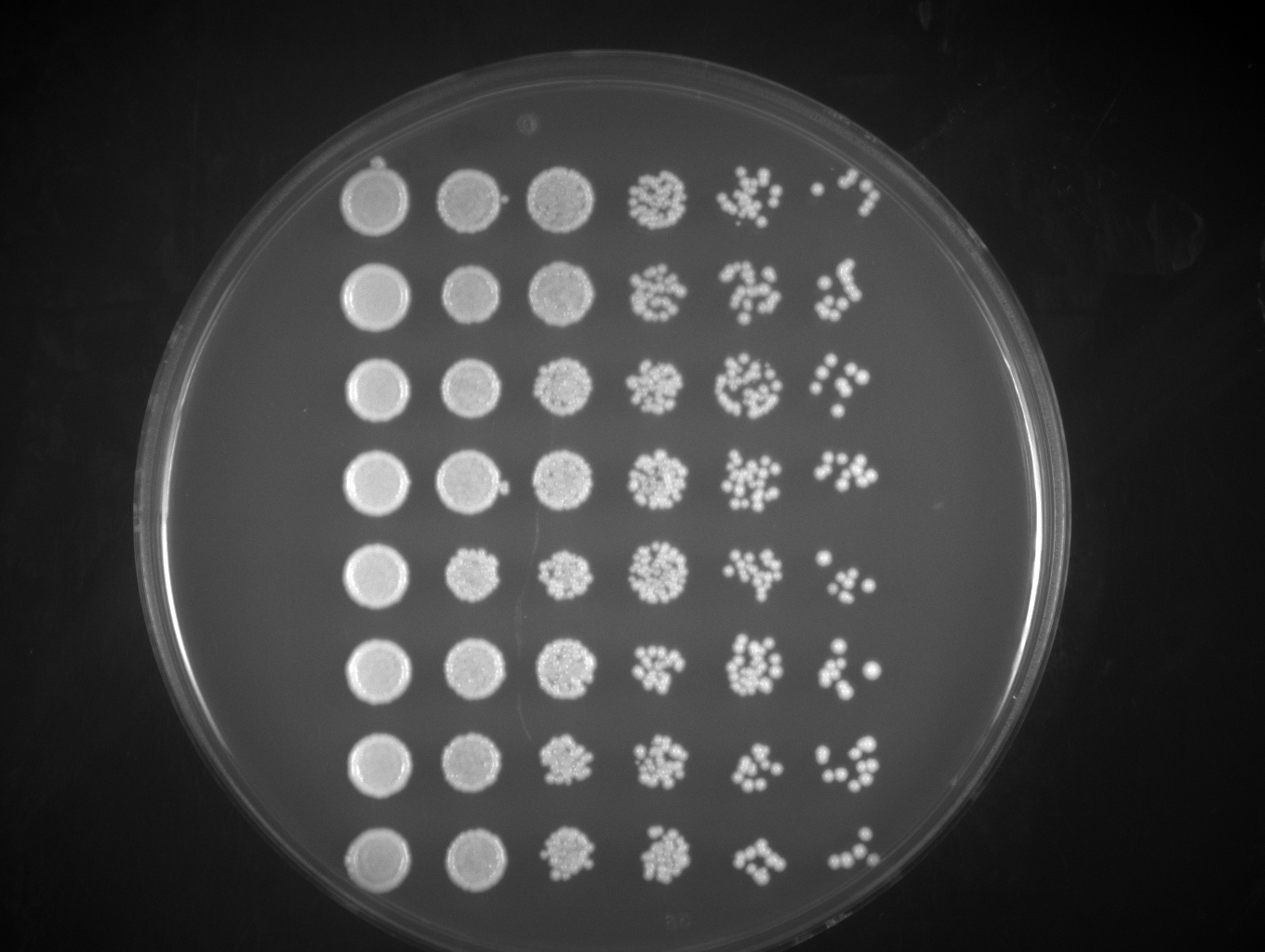

Supplement: Supplementary file 3 — Source data Fig. 1 [file 44318_2025_437_MOESM3_ESM.zip › Figure1/1E/30 °C plate.tif]

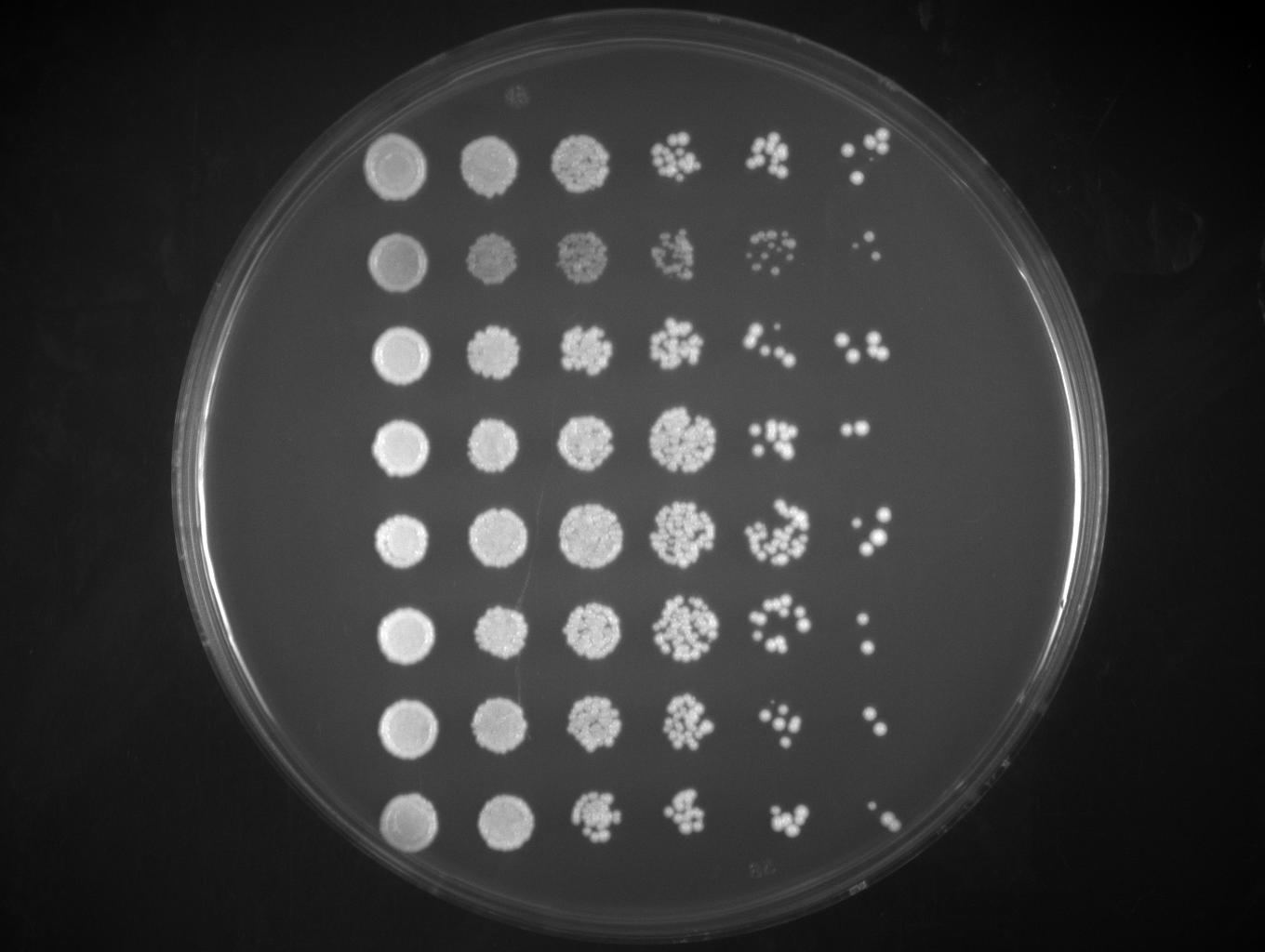

Supplement: Supplementary file 3 — Source data Fig. 1 [file 44318_2025_437_MOESM3_ESM.zip › Figure1/1E/37 °C plate.tif]

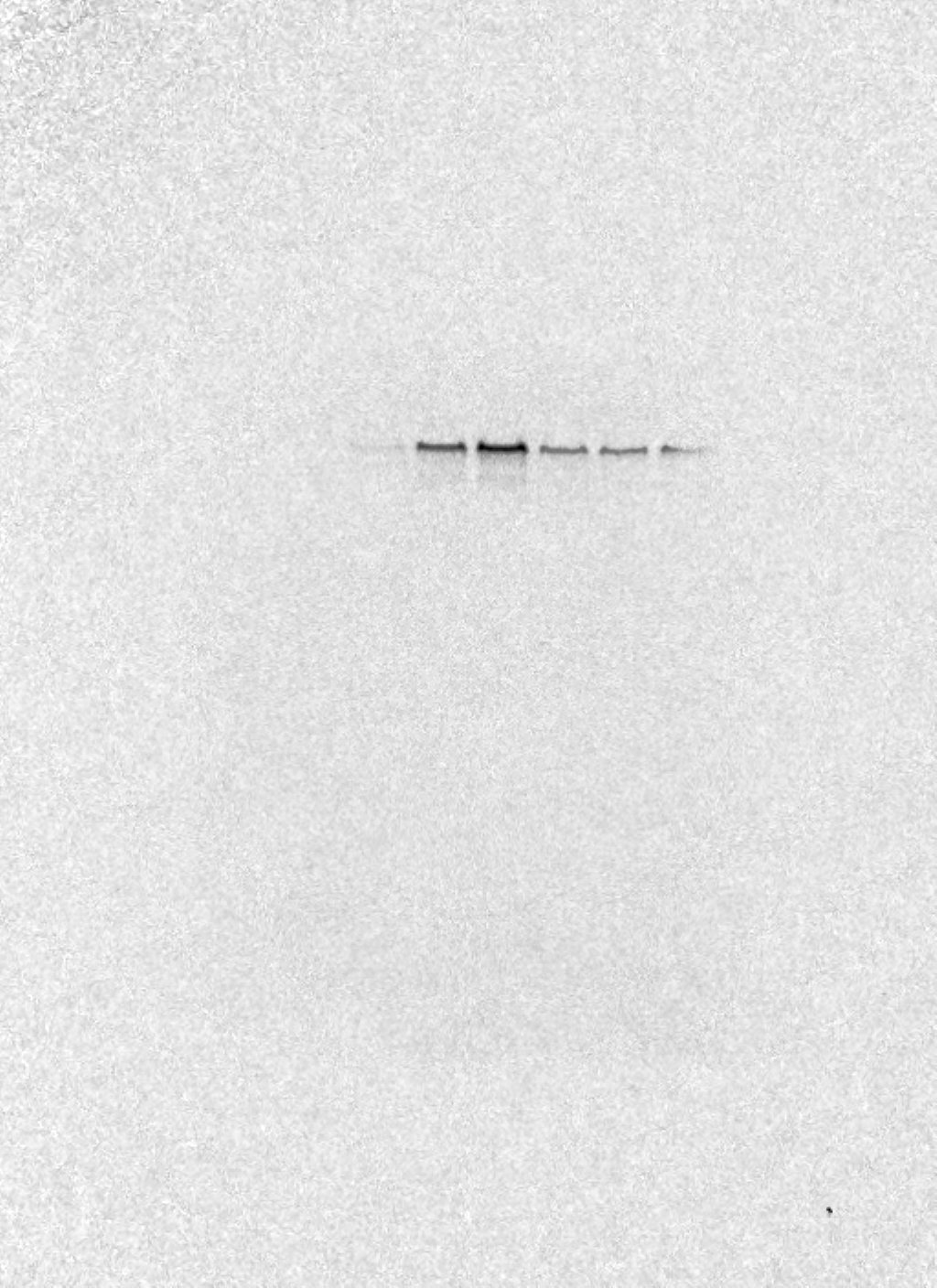

Supplement: Supplementary file 4 — Source data Fig. 2 [file 44318_2025_437_MOESM4_ESM.zip › Figure2/2A/Blot_deltaMad1_myc.tif]

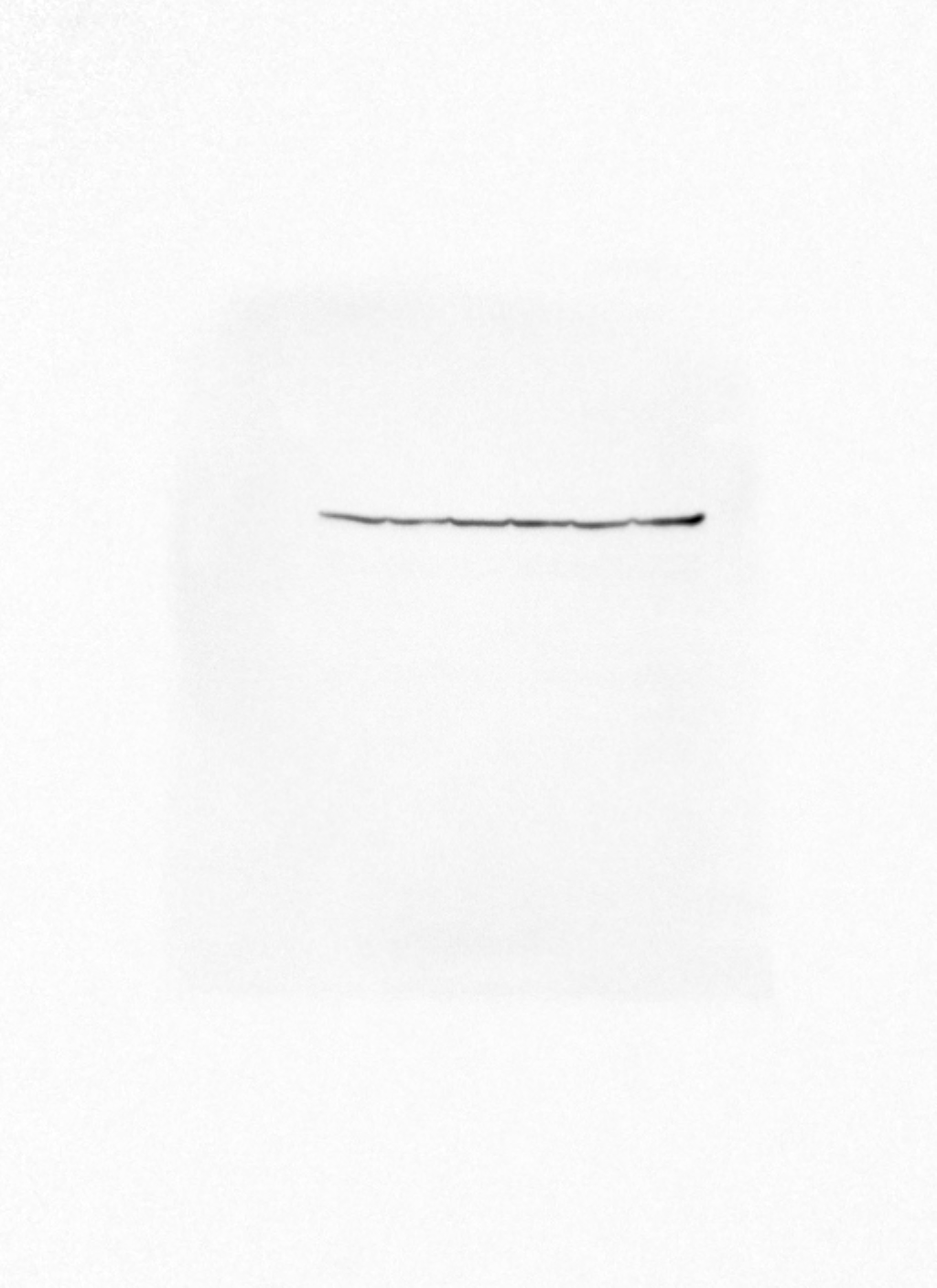

Supplement: Supplementary file 4 — Source data Fig. 2 [file 44318_2025_437_MOESM4_ESM.zip › Figure2/2A/Blot_deltaMad1_PGK1.tif]

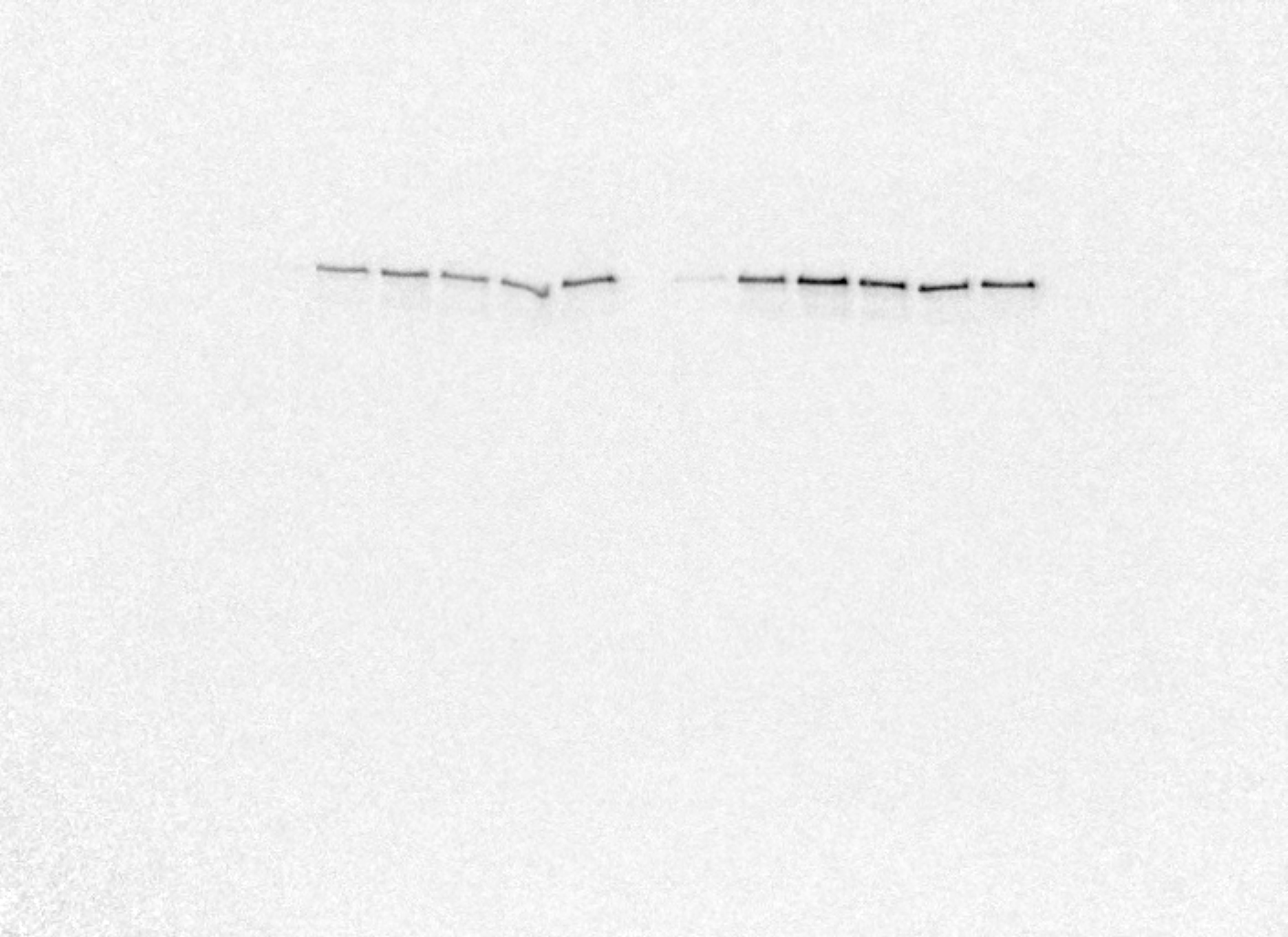

Supplement: Supplementary file 4 — Source data Fig. 2 [file 44318_2025_437_MOESM4_ESM.zip › Figure2/2A/Blot_WT_deltaZwint_myc.tif]

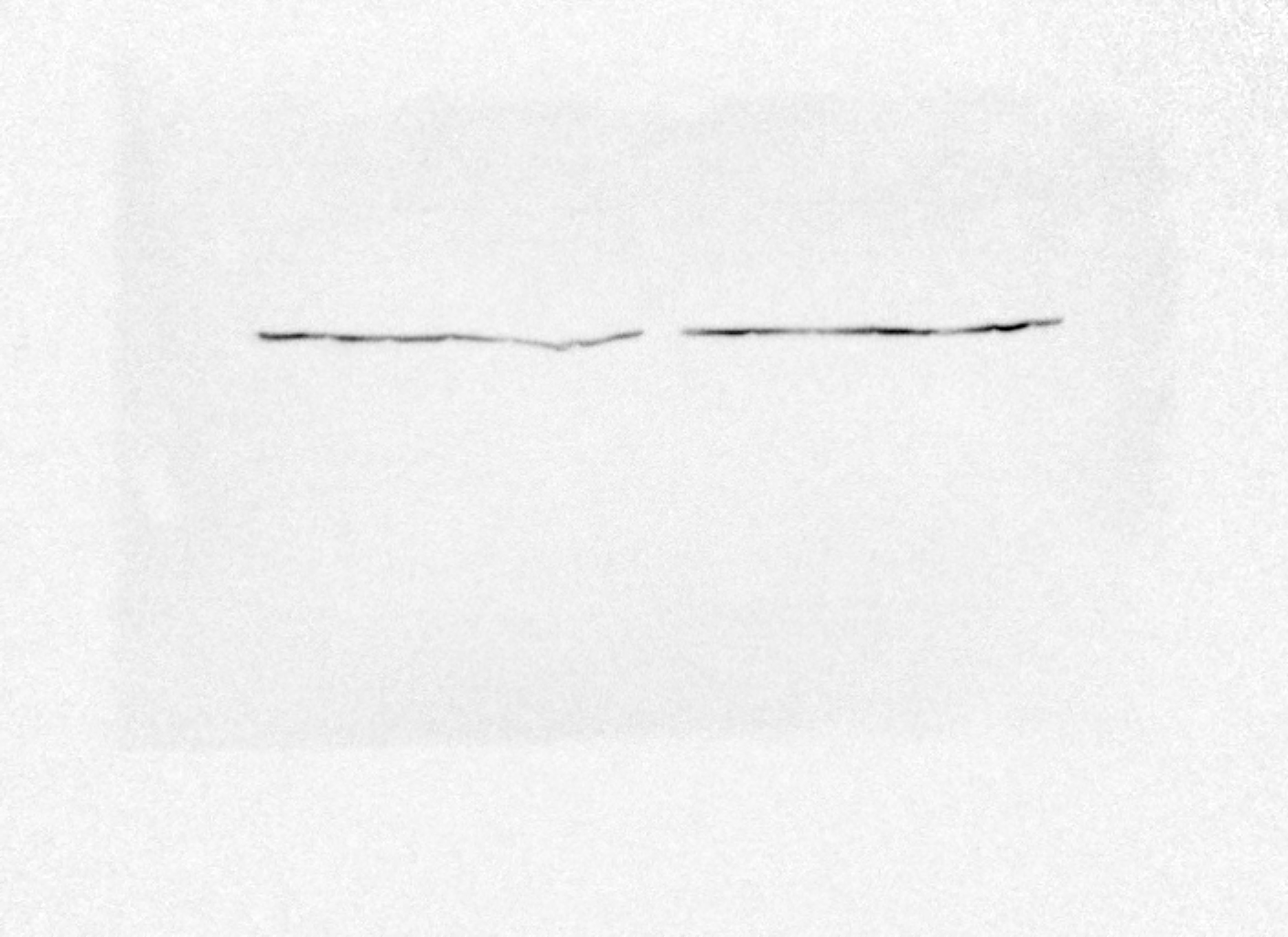

Supplement: Supplementary file 4 — Source data Fig. 2 [file 44318_2025_437_MOESM4_ESM.zip › Figure2/2A/Blot_WT_deltaZwint_PGK1.tif]

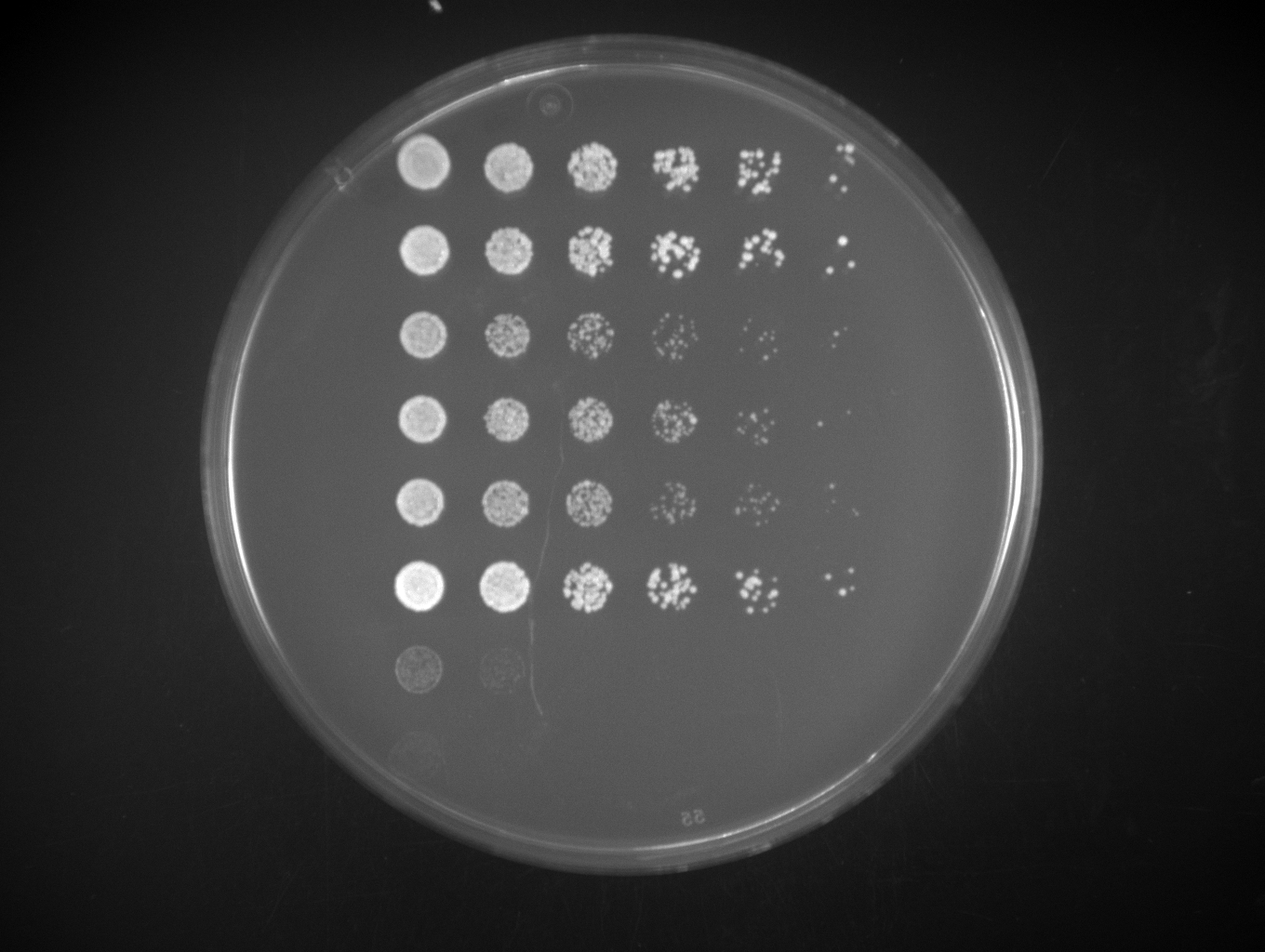

Supplement: Supplementary file 4 — Source data Fig. 2 [file 44318_2025_437_MOESM4_ESM.zip › Figure2/2B/WholeMount_16_5days.tif]

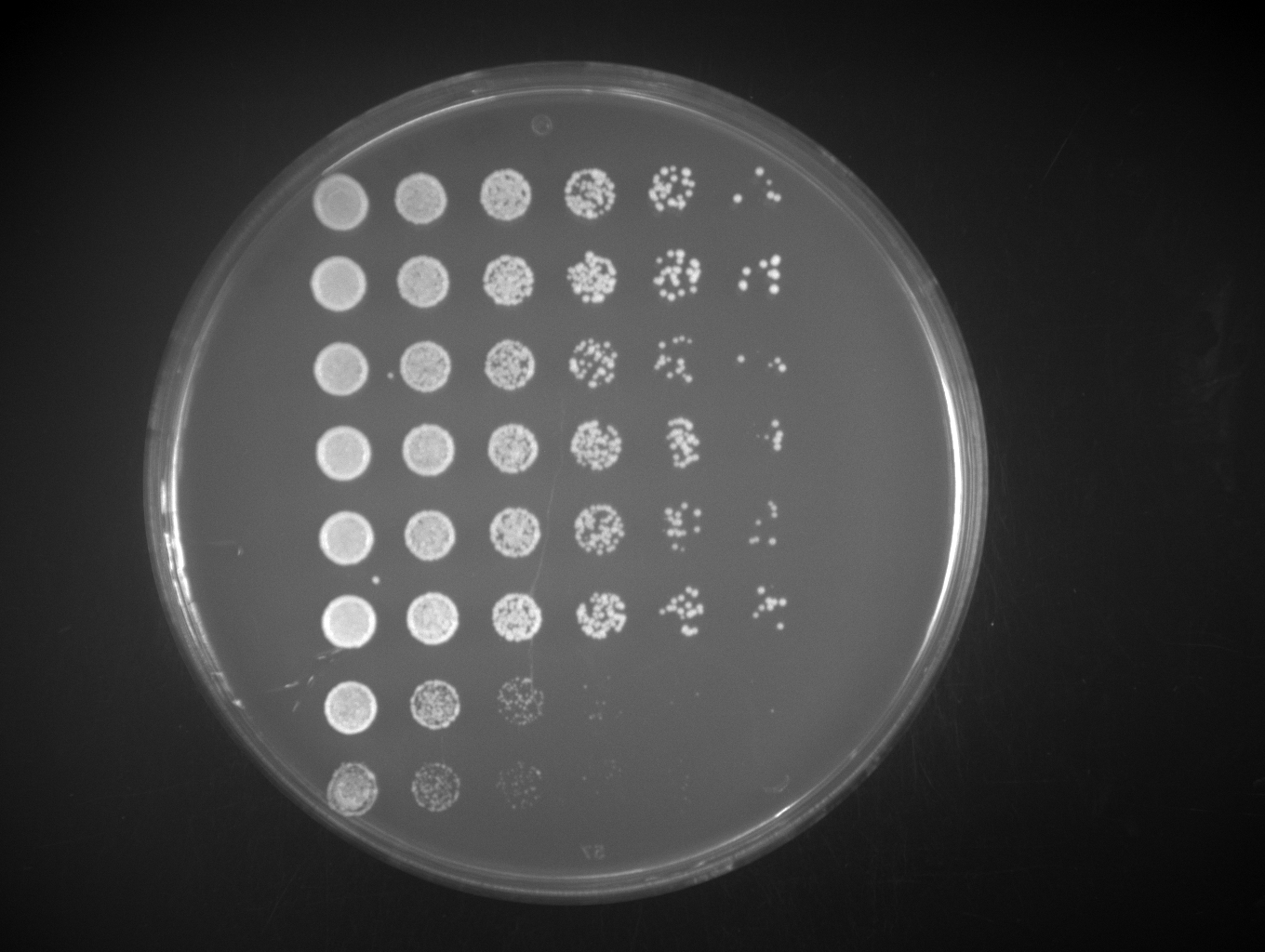

Supplement: Supplementary file 4 — Source data Fig. 2 [file 44318_2025_437_MOESM4_ESM.zip › Figure2/2B/WholeMount_25C.tif]

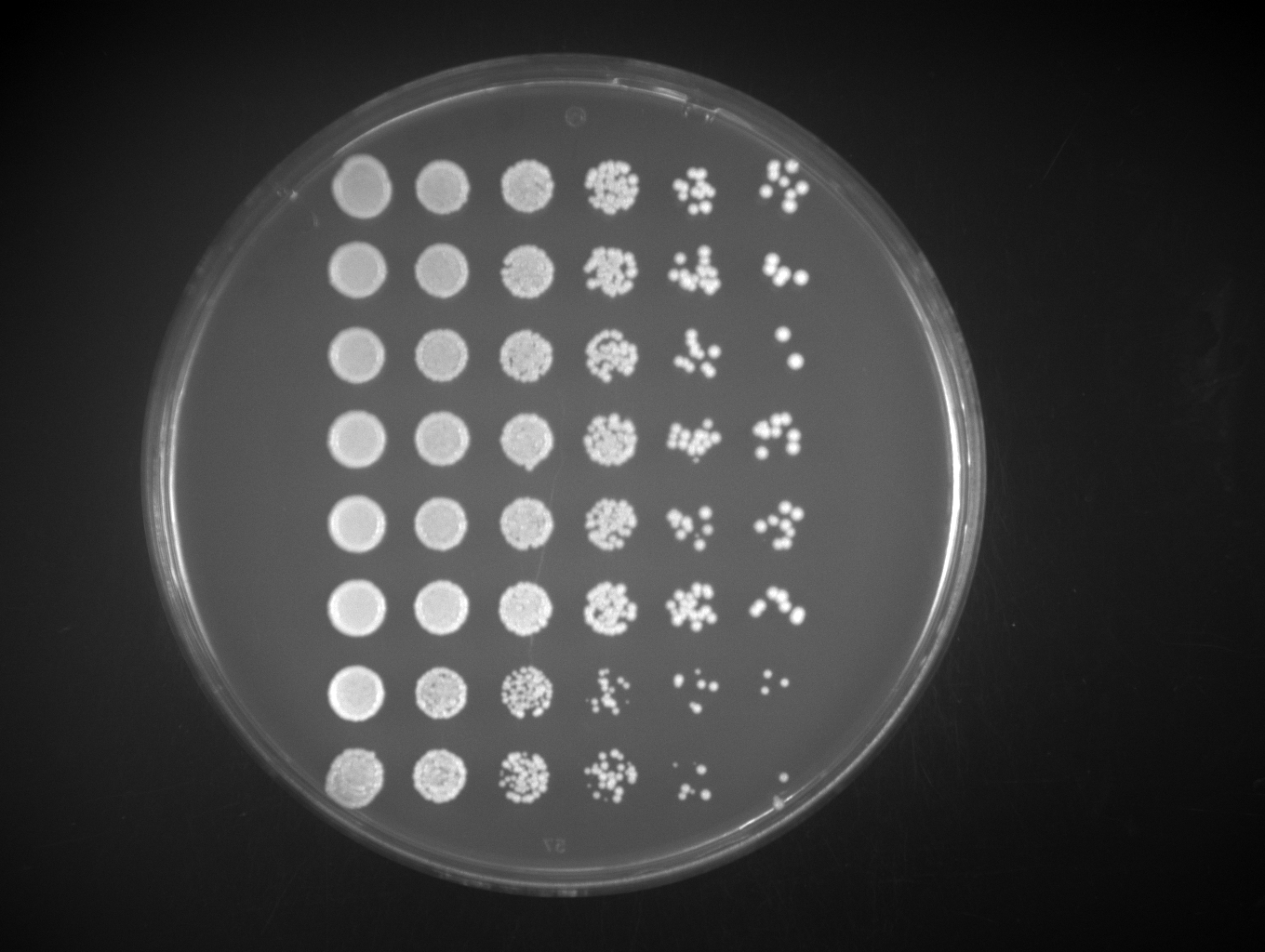

Supplement: Supplementary file 4 — Source data Fig. 2 [file 44318_2025_437_MOESM4_ESM.zip › Figure2/2B/WholeMount_30C.tif]

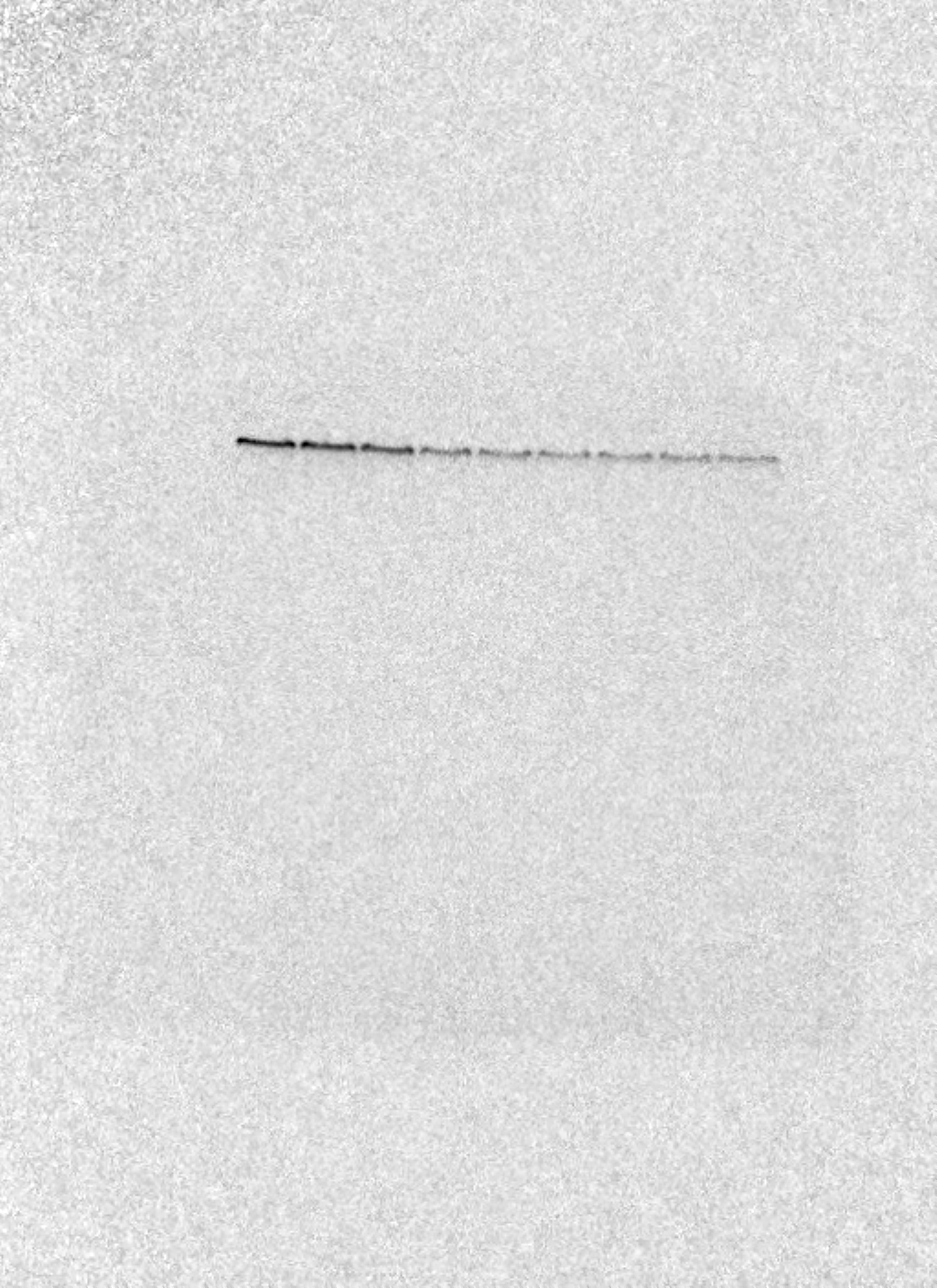

Supplement: Supplementary file 4 — Source data Fig. 2 [file 44318_2025_437_MOESM4_ESM.zip › Figure2/2C/Blot_deltaZwint_myc.tif]

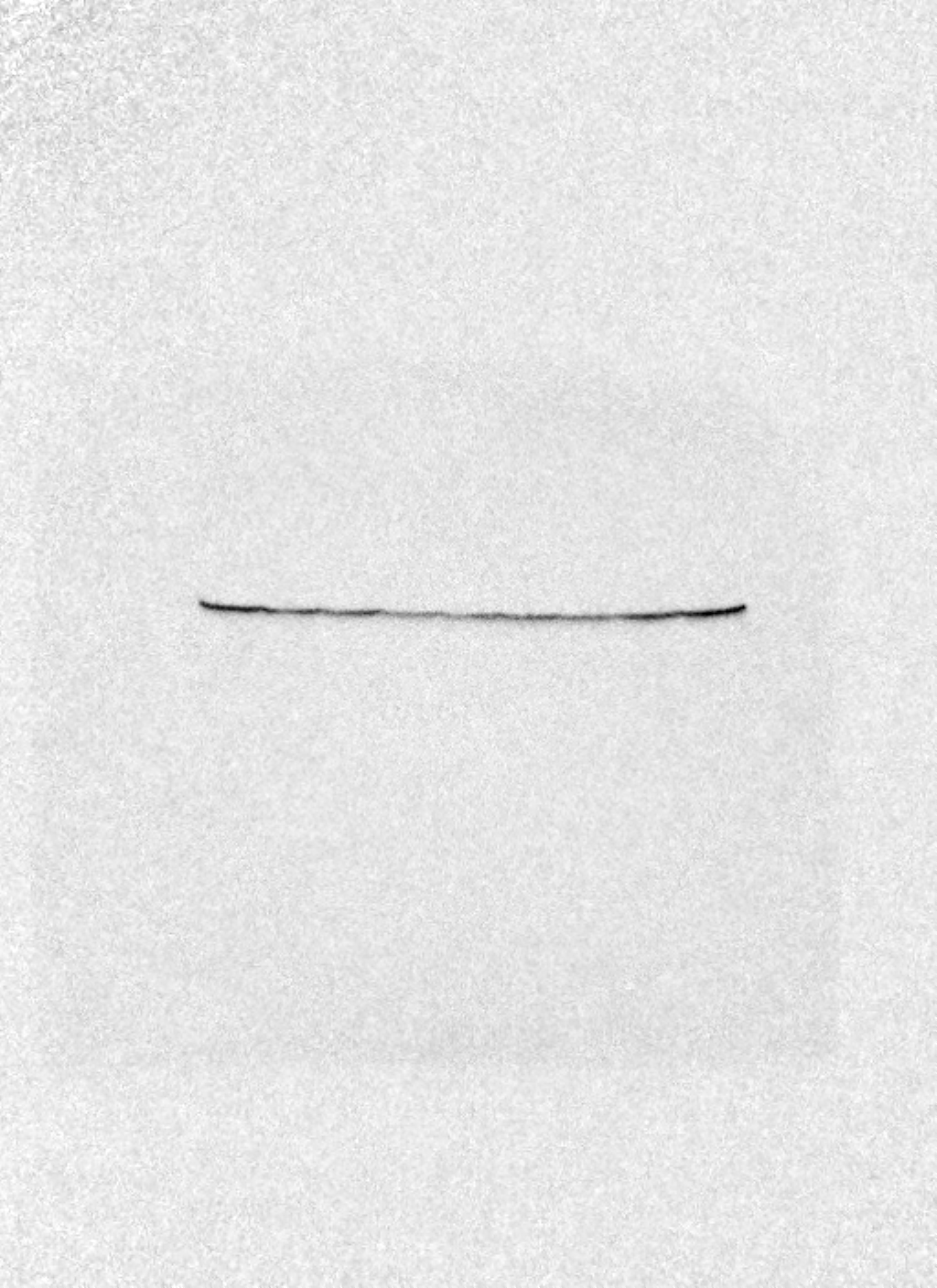

Supplement: Supplementary file 4 — Source data Fig. 2 [file 44318_2025_437_MOESM4_ESM.zip › Figure2/2C/Blot_deltaZwint_PGK1.tif]

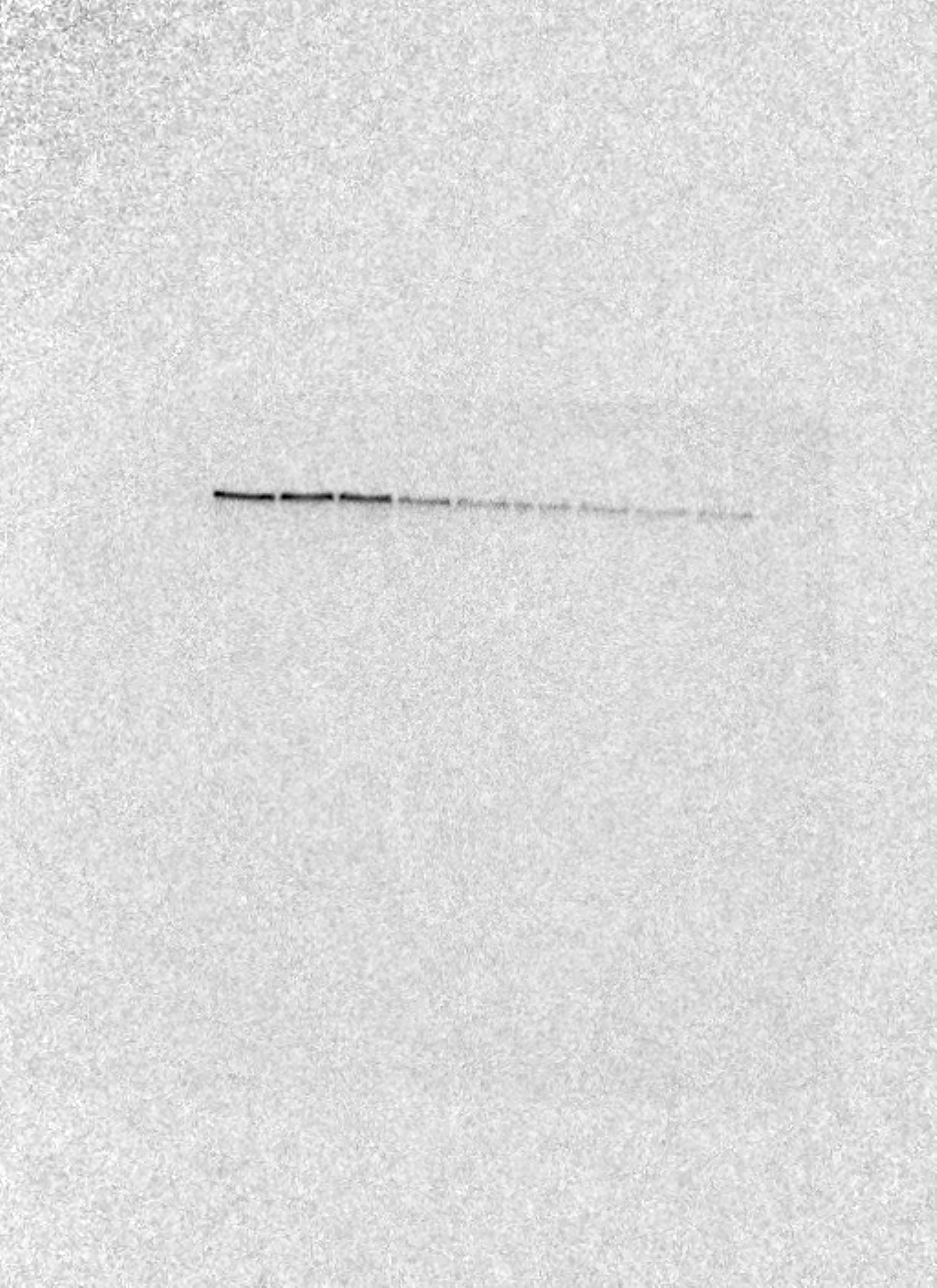

Supplement: Supplementary file 4 — Source data Fig. 2 [file 44318_2025_437_MOESM4_ESM.zip › Figure2/2C/Blot_wt_myc.tif]

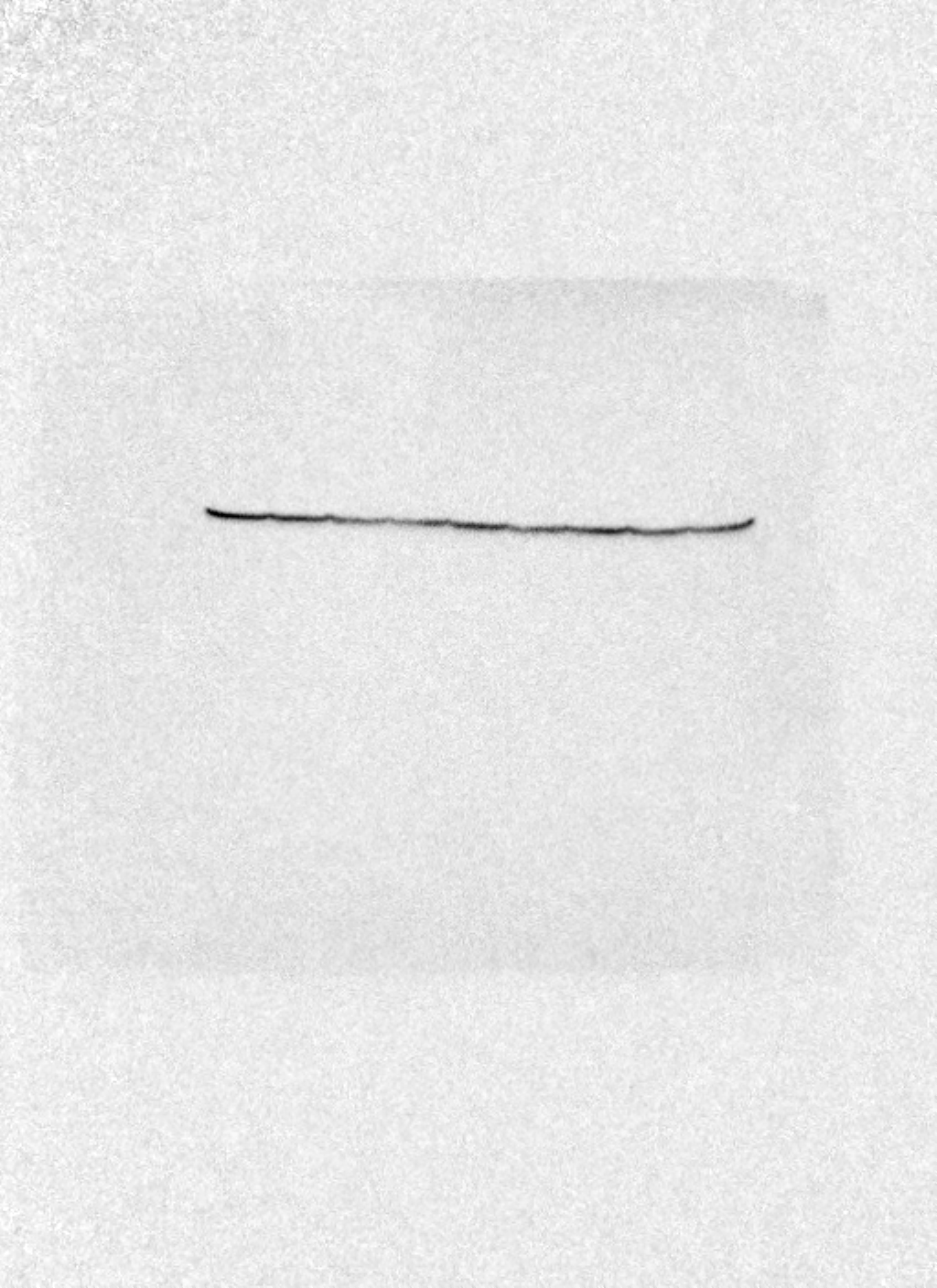

Supplement: Supplementary file 4 — Source data Fig. 2 [file 44318_2025_437_MOESM4_ESM.zip › Figure2/2C/Blot_wt_PGK1.tif]

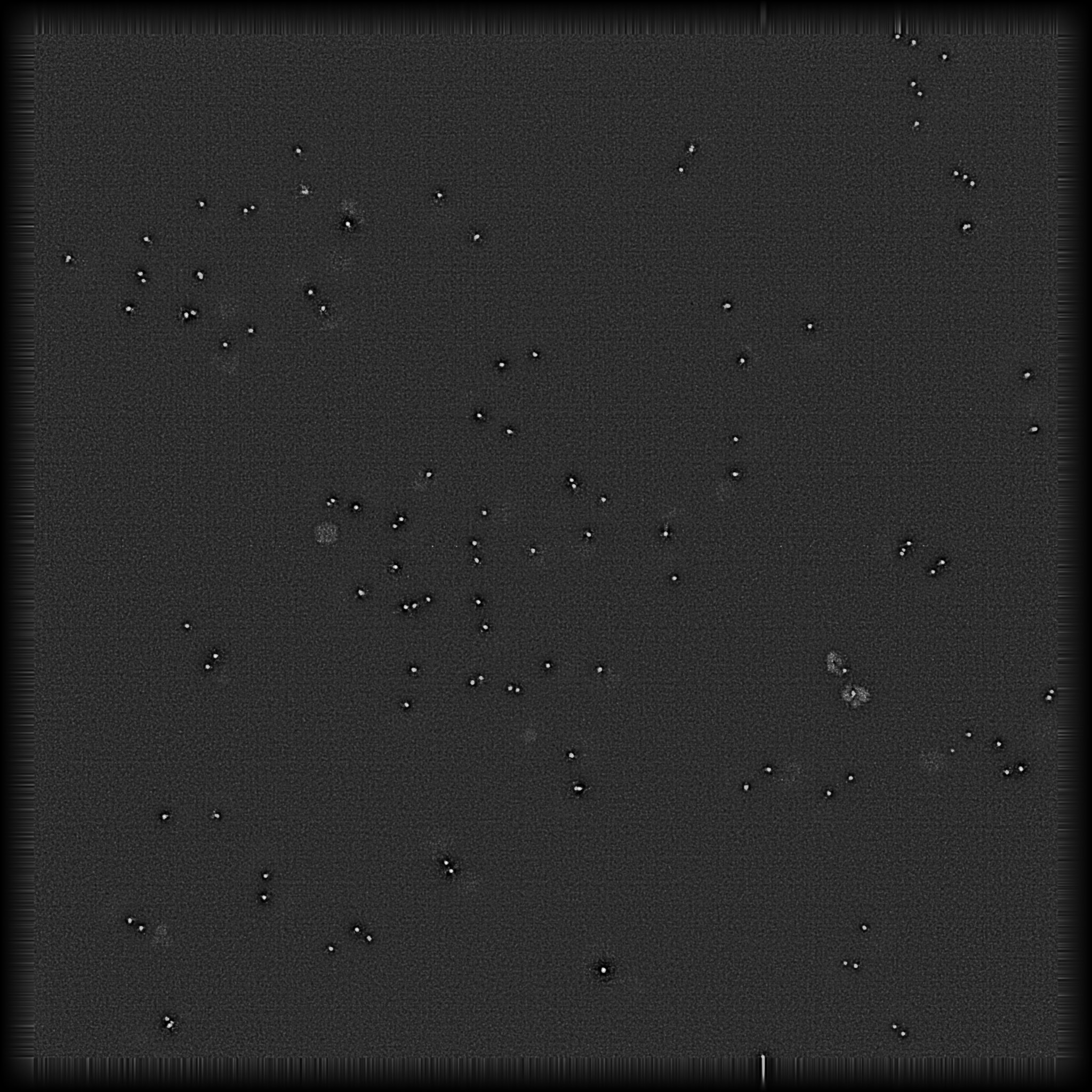

Supplement: Supplementary file 4 — Source data Fig. 2 [file 44318_2025_437_MOESM4_ESM.zip › Figure2/2D/WholeMount_mCherry_GFP_POL.tif]

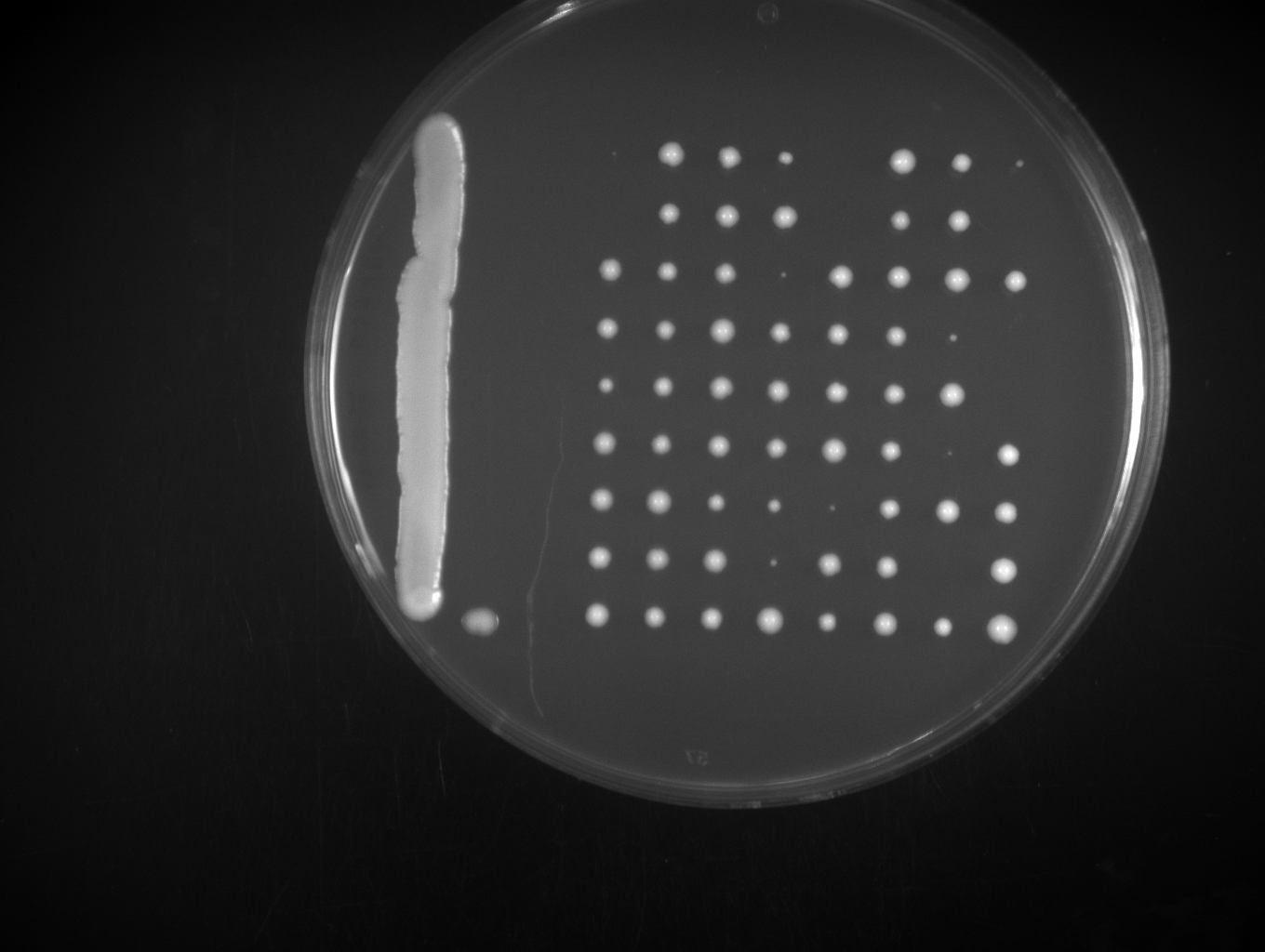

Supplement: Supplementary file 4 — Source data Fig. 2 [file 44318_2025_437_MOESM4_ESM.zip › Figure2/2F/WholeMount_Kre28_deltaZwint.tif]

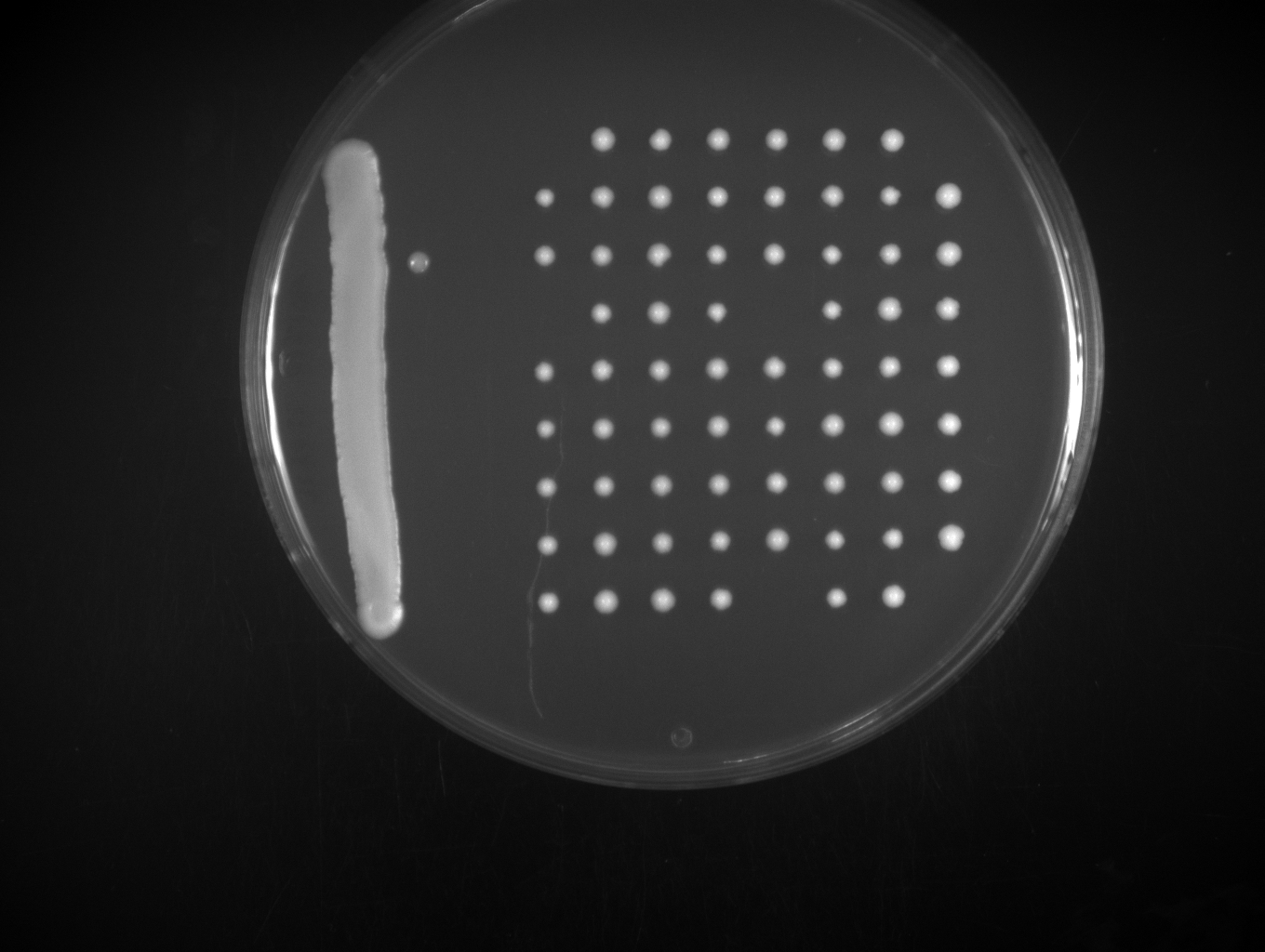

Supplement: Supplementary file 4 — Source data Fig. 2 [file 44318_2025_437_MOESM4_ESM.zip › Figure2/2F/WholeMount_Kre28_wt.tif]

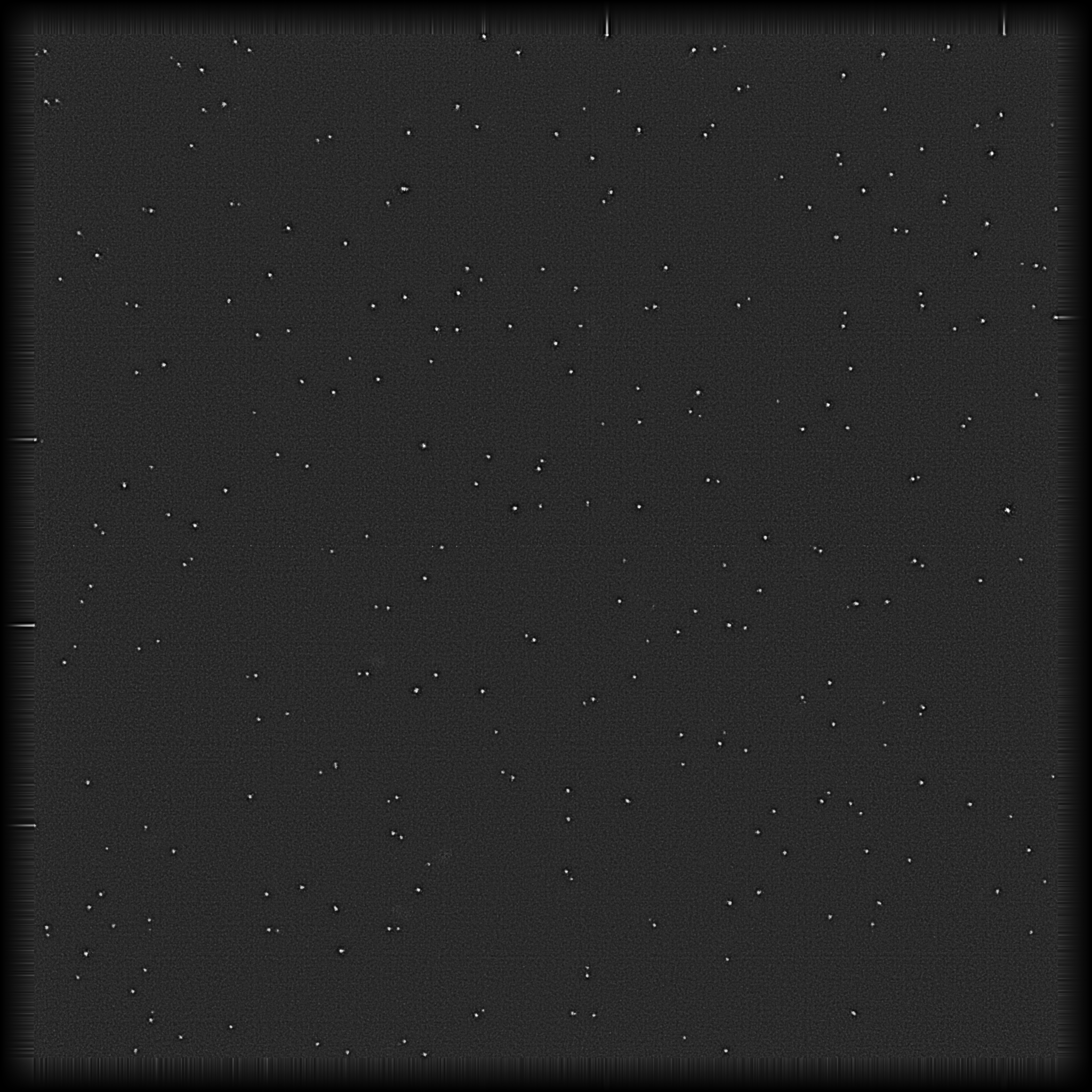

Supplement: Supplementary file 5 — Source data Fig. 3 [file 44318_2025_437_MOESM5_ESM.zip › Figure3/3A/WholeMount_Mtw1-GFP_deltaZwint_RFP_GFP_POL.tif]

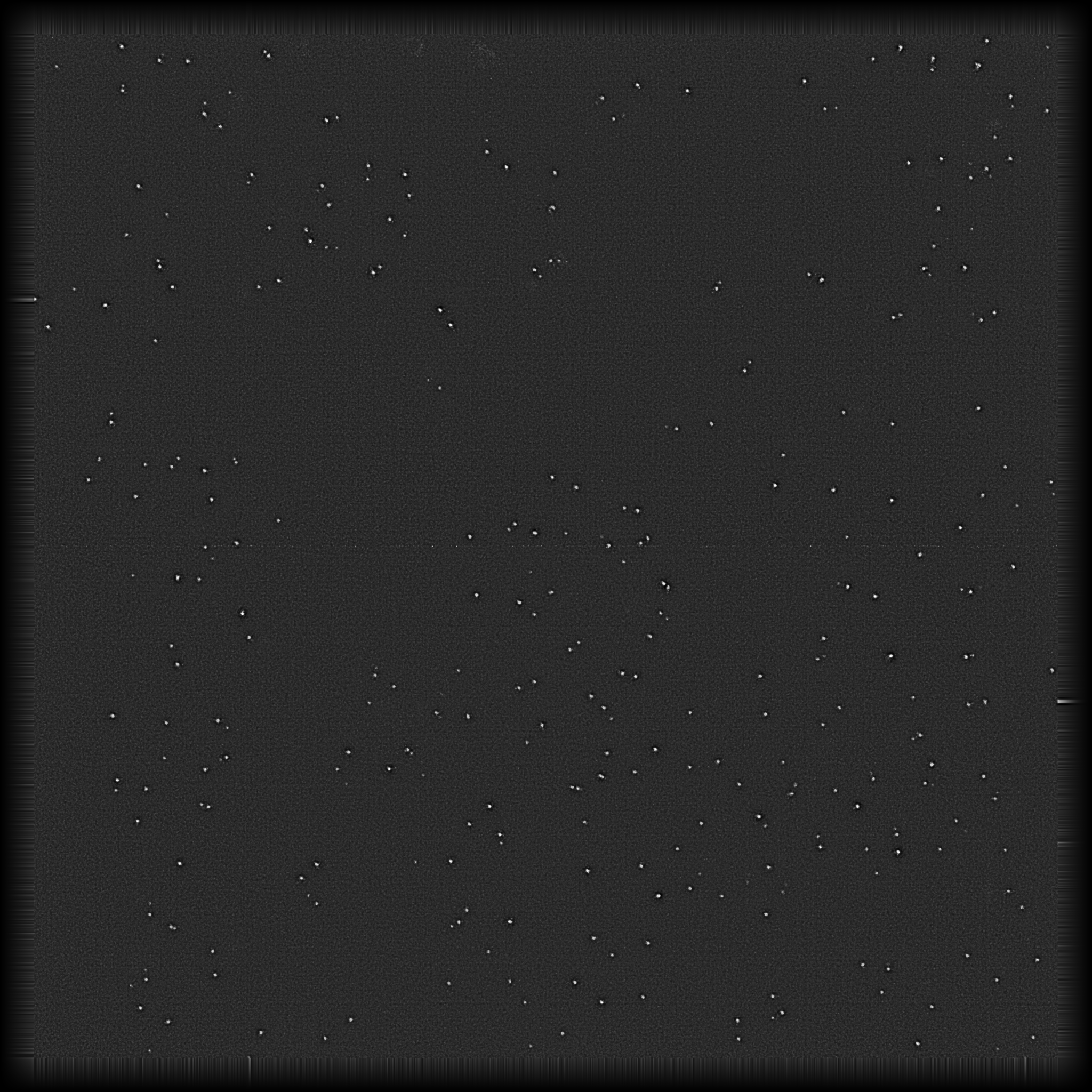

Supplement: Supplementary file 5 — Source data Fig. 3 [file 44318_2025_437_MOESM5_ESM.zip › Figure3/3A/WholeMount_Mtw1-GFP_wt_RFP_GFP_POL.tif]

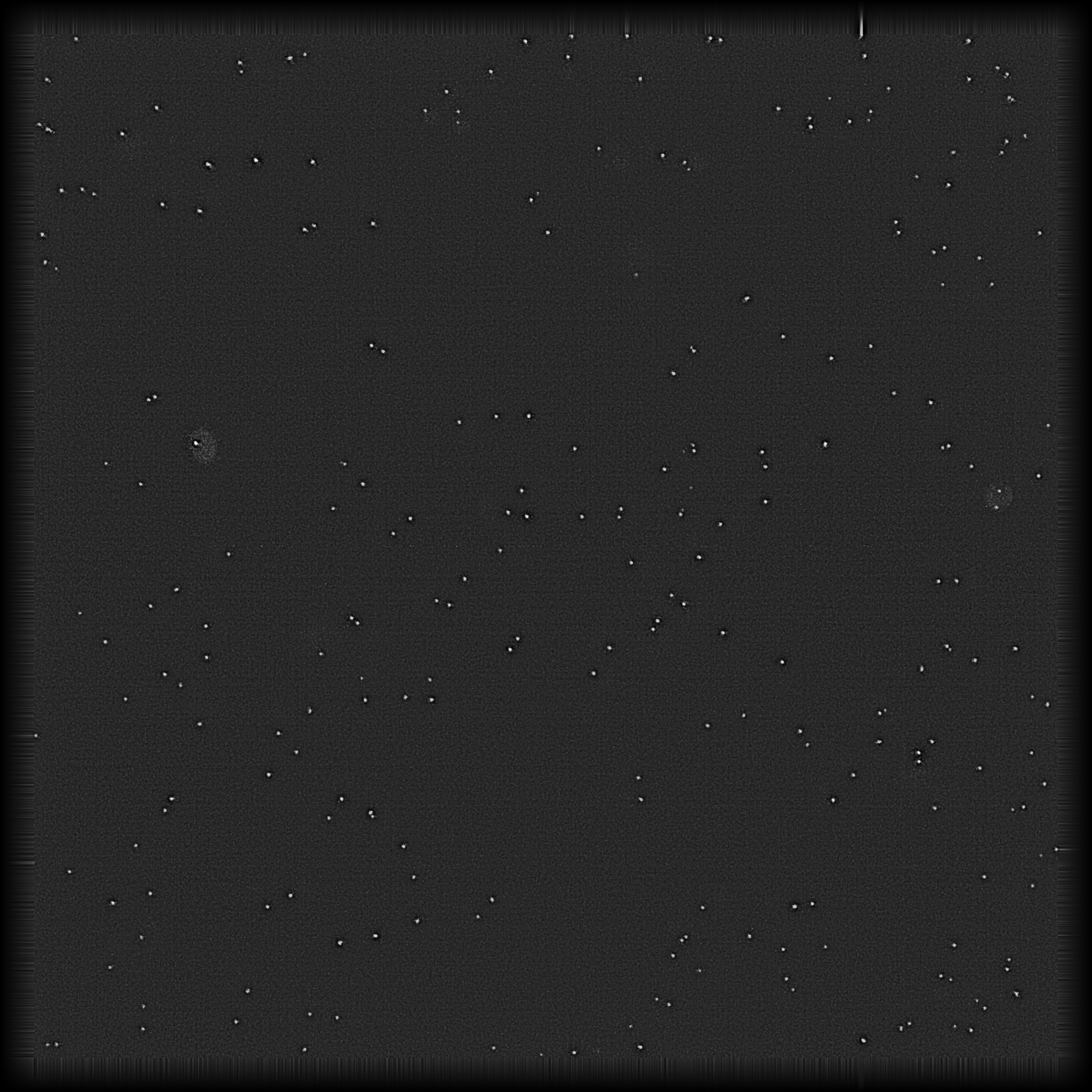

Supplement: Supplementary file 5 — Source data Fig. 3 [file 44318_2025_437_MOESM5_ESM.zip › Figure3/3A/WholeMount_Nuf2-GFP_deltaZwint_RFP_GFP_POL.tif]

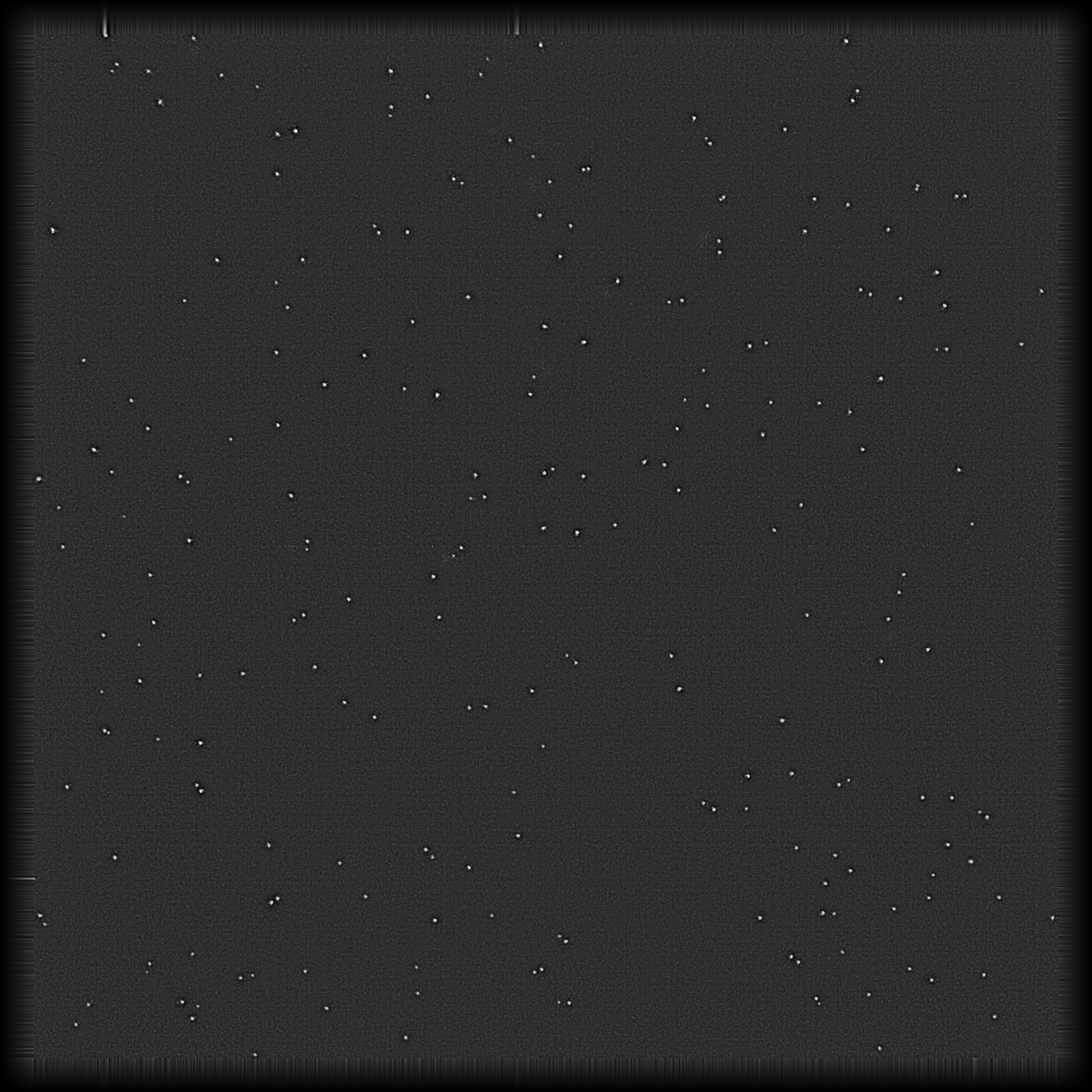

Supplement: Supplementary file 5 — Source data Fig. 3 [file 44318_2025_437_MOESM5_ESM.zip › Figure3/3A/WholeMount_Nuf2-GFP_wt_RFP_GFP_POL.tif]

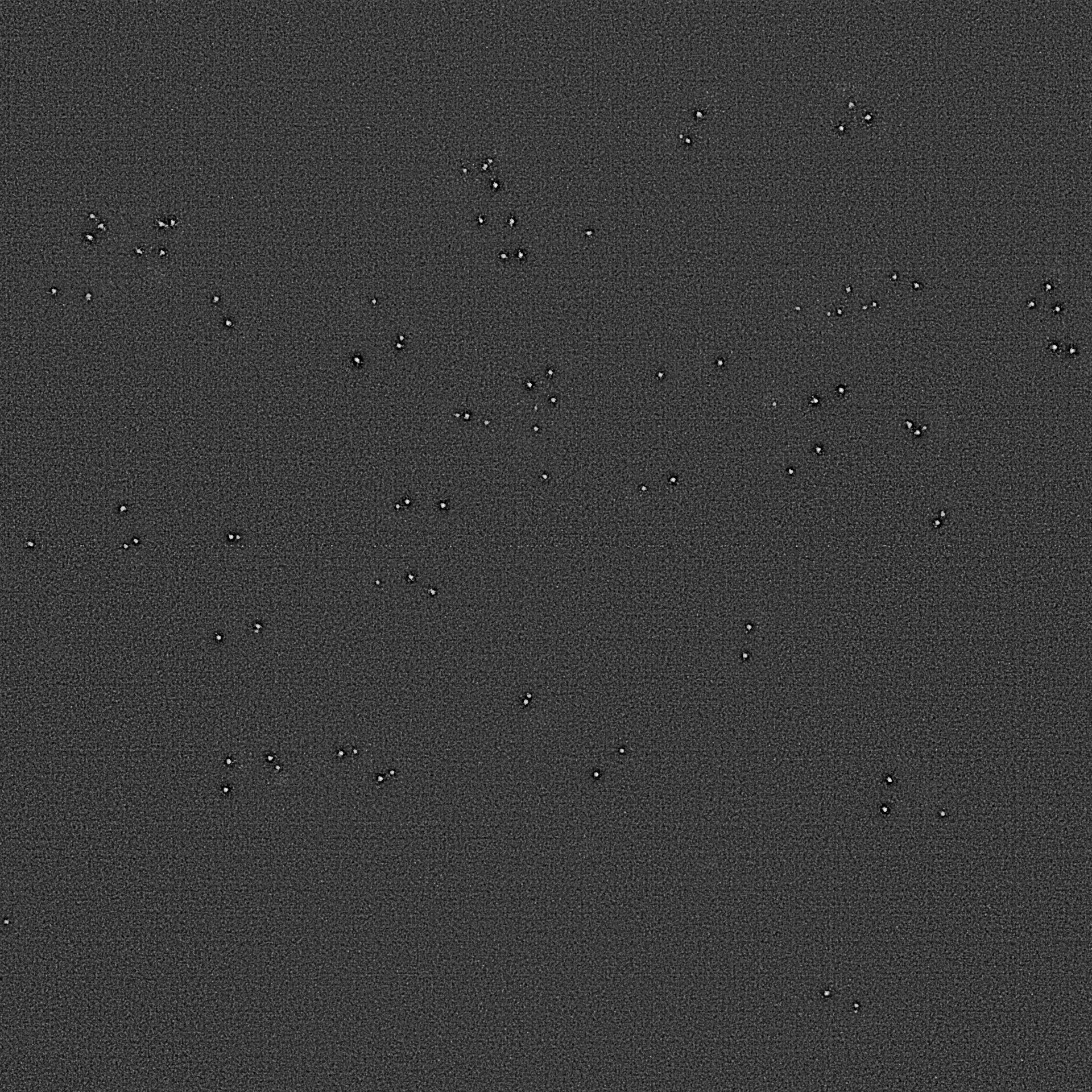

Supplement: Supplementary file 5 — Source data Fig. 3 [file 44318_2025_437_MOESM5_ESM.zip › Figure3/3B/WholeMount_GFP-Kre28_deltaZwint_RFP_GFP_POL.tif]

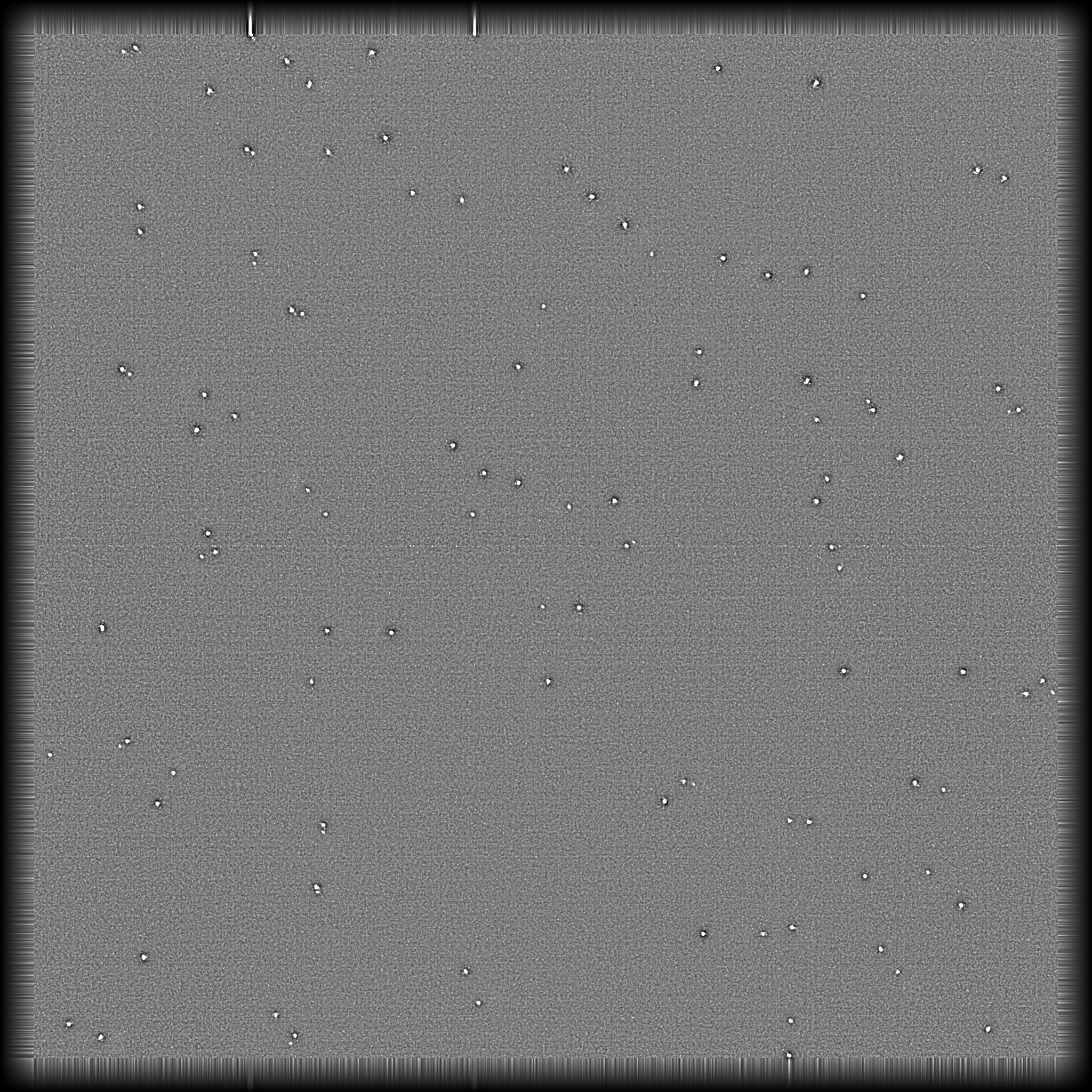

Supplement: Supplementary file 5 — Source data Fig. 3 [file 44318_2025_437_MOESM5_ESM.zip › Figure3/3B/WholeMount_GFP-Kre28_wt_RFP_GFP_POL.tif]

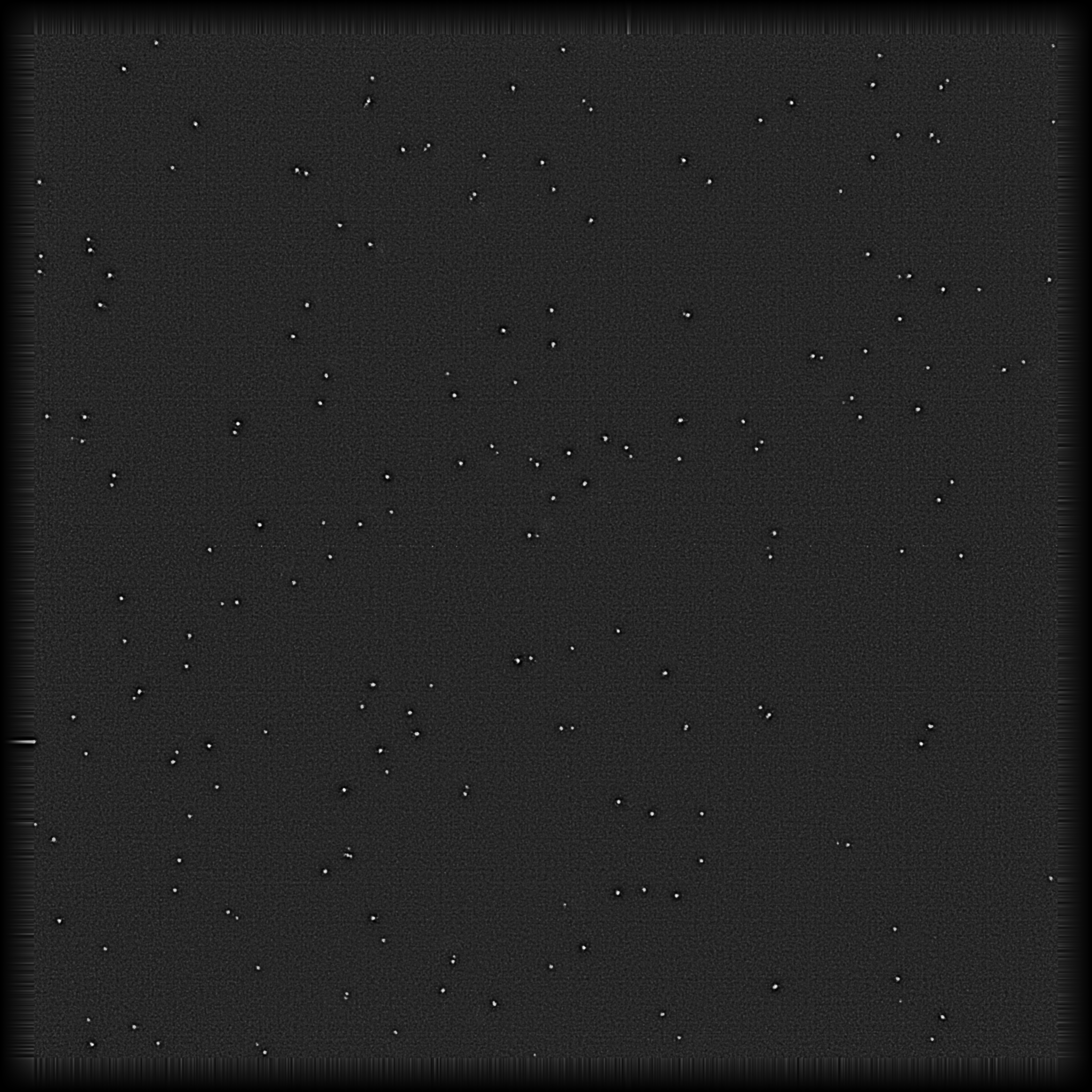

Supplement: Supplementary file 5 — Source data Fig. 3 [file 44318_2025_437_MOESM5_ESM.zip › Figure3/3B/WholeMount_Spc105-GFP_deltaZwint_RFP_GFP_POL.tif]

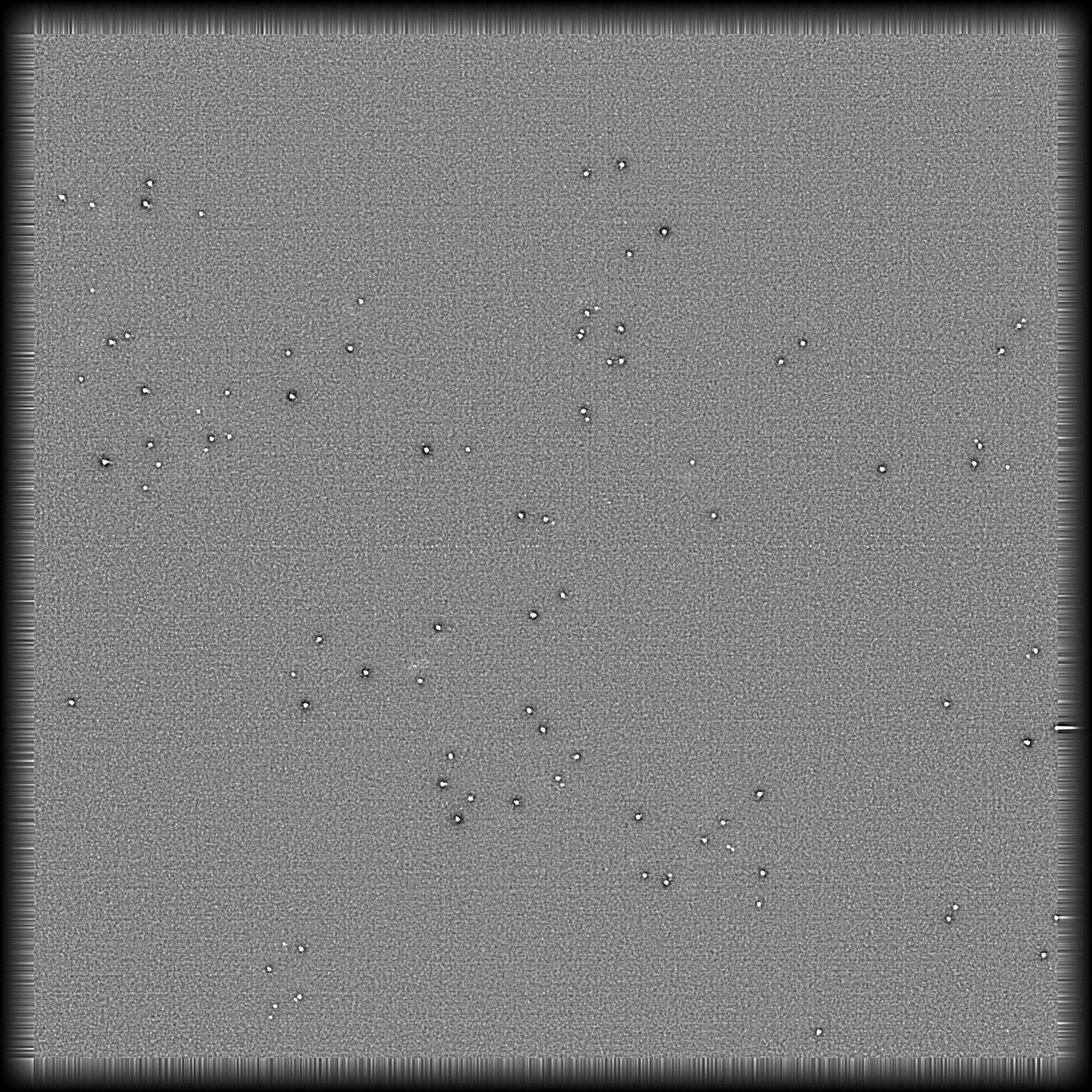

Supplement: Supplementary file 5 — Source data Fig. 3 [file 44318_2025_437_MOESM5_ESM.zip › Figure3/3B/WholeMount_Spc105-GFP_wt_RFP_GFP_POL.tif]

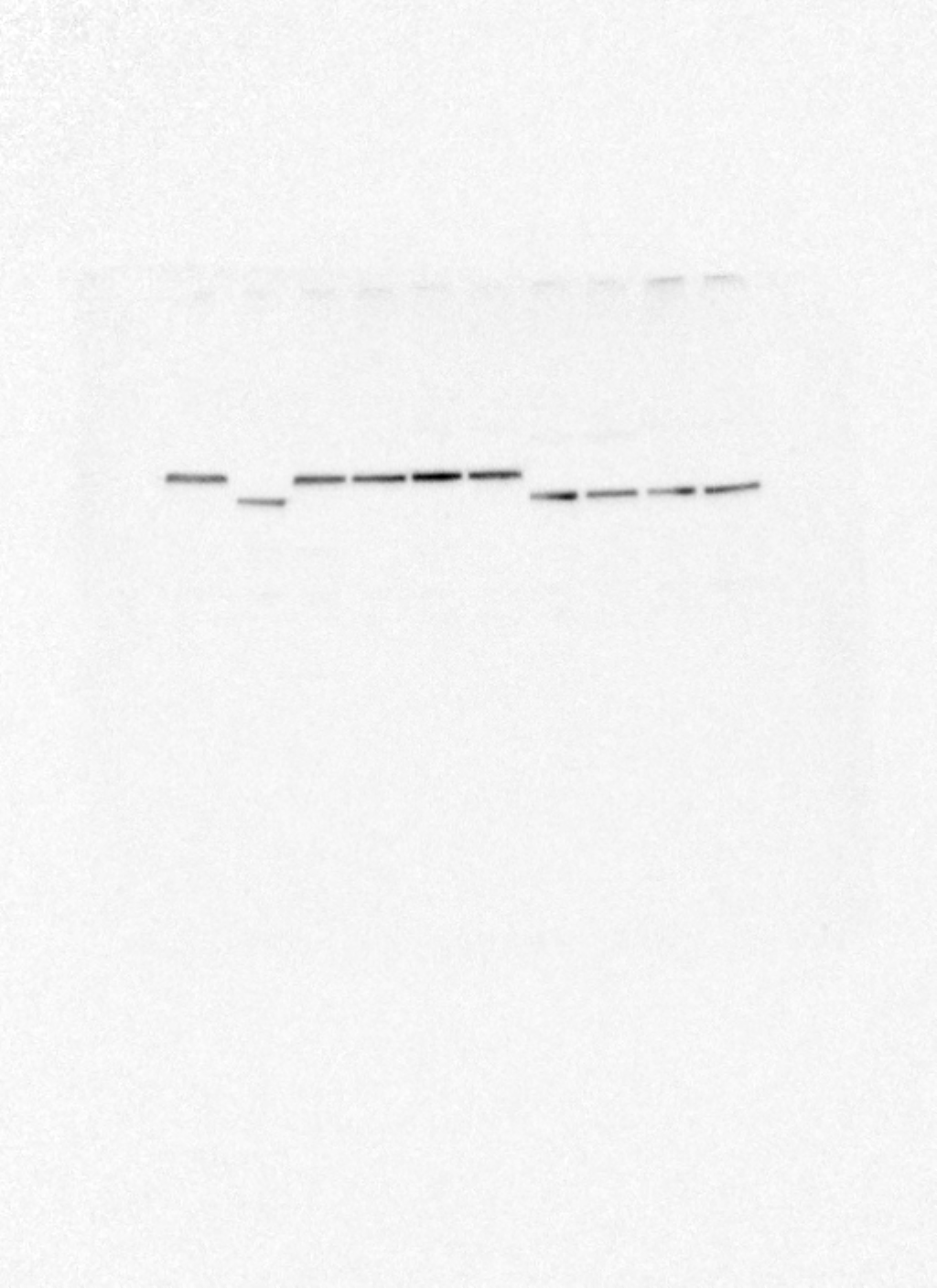

Supplement: Supplementary file 6 — Source data Fig. 4 [file 44318_2025_437_MOESM6_ESM.zip › Figure4/4B/Blot_anti_Flag.tif]

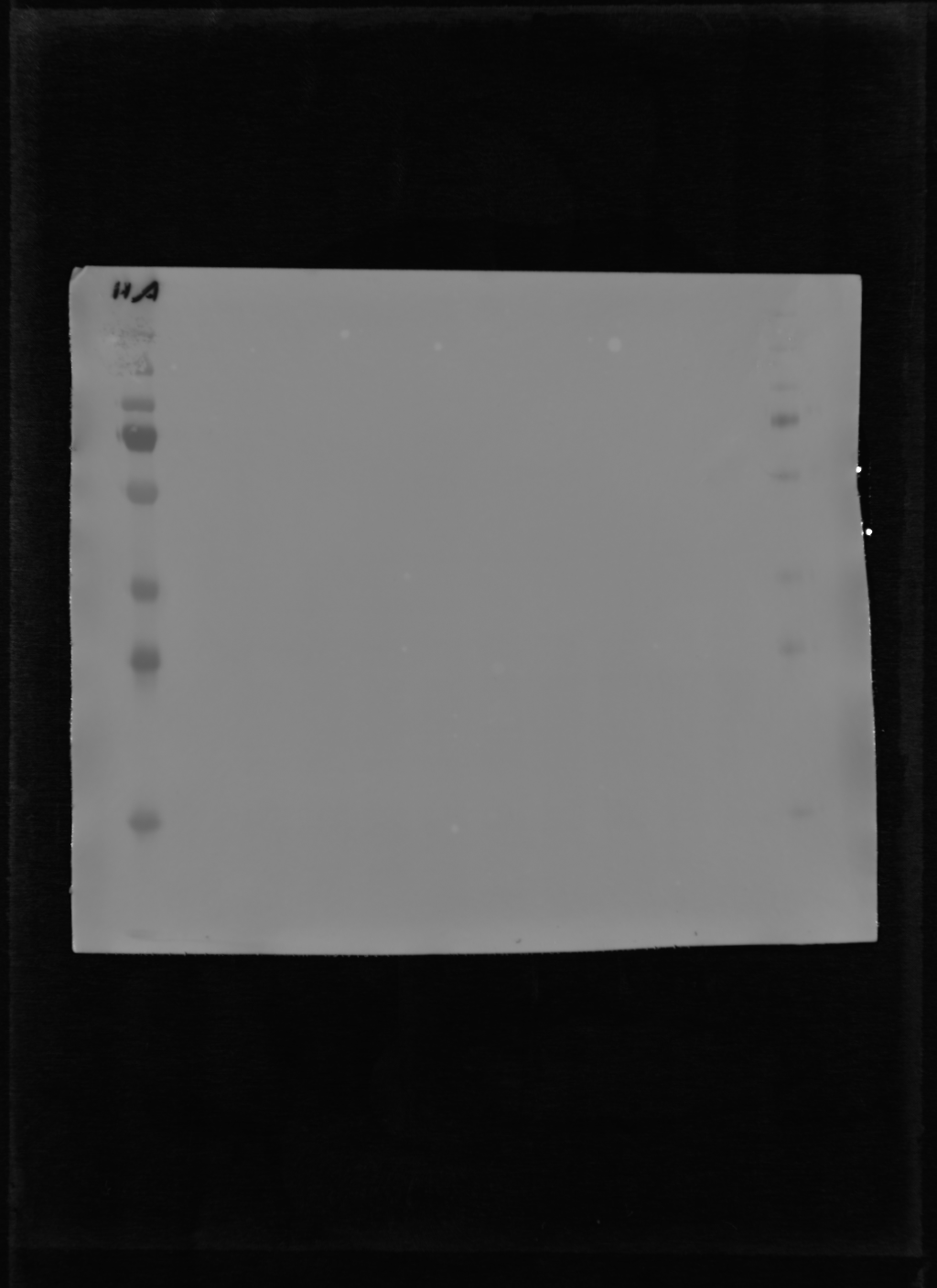

Supplement: Supplementary file 6 — Source data Fig. 4 [file 44318_2025_437_MOESM6_ESM.zip › Figure4/4B/Blot_anti_Flag-Marker.tif]

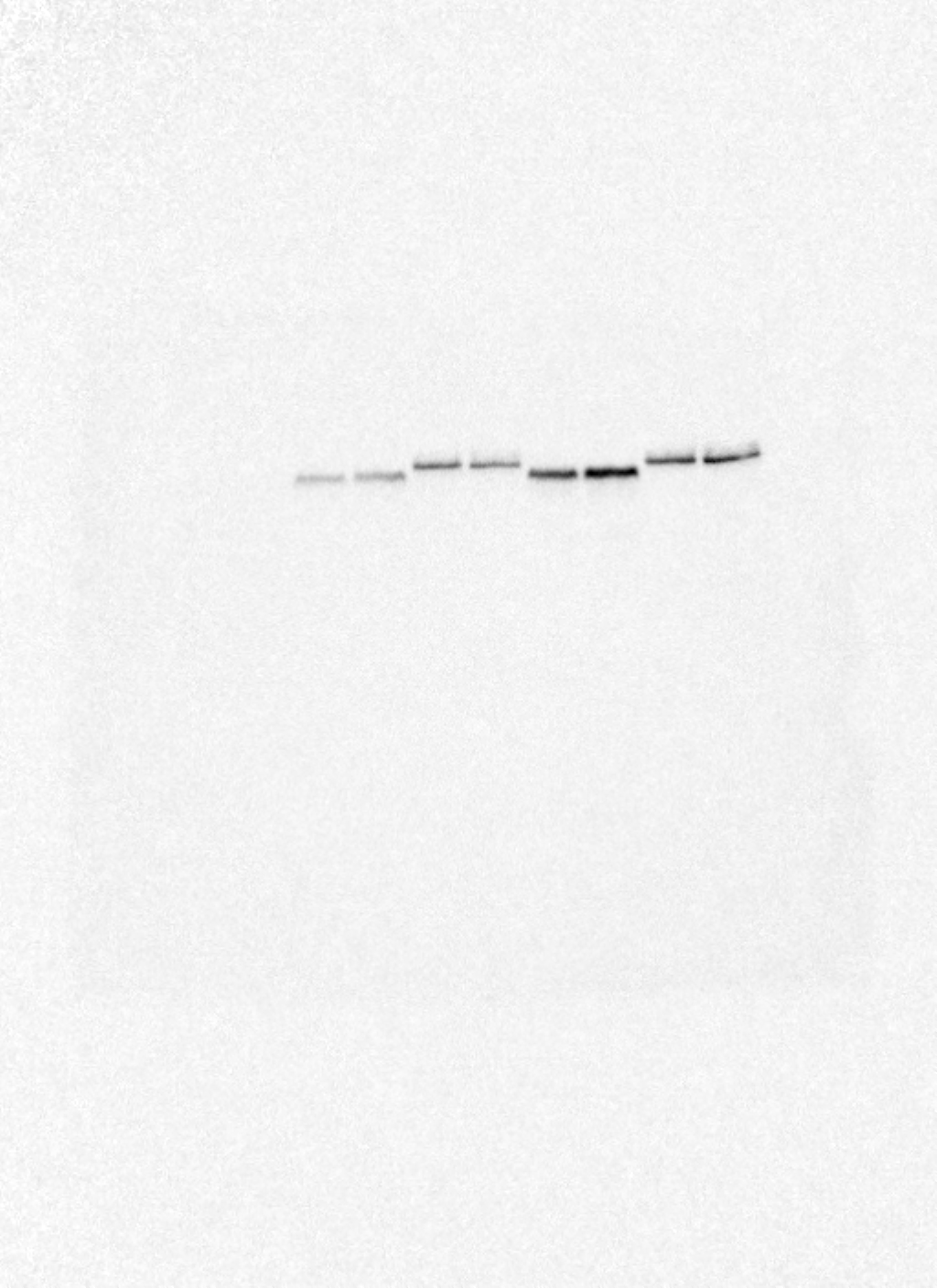

Supplement: Supplementary file 6 — Source data Fig. 4 [file 44318_2025_437_MOESM6_ESM.zip › Figure4/4B/Blot_anti_HA.tif]

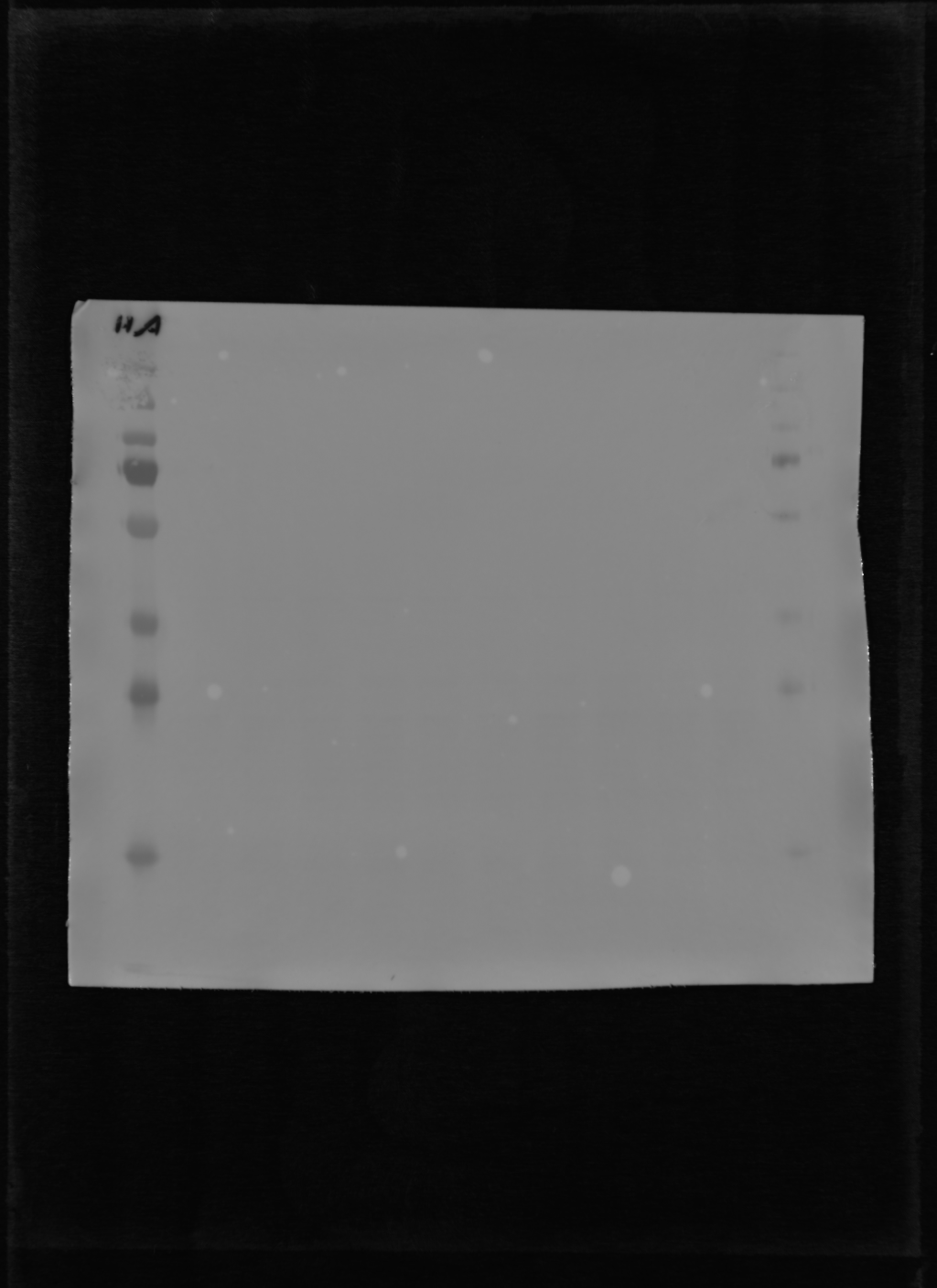

Supplement: Supplementary file 6 — Source data Fig. 4 [file 44318_2025_437_MOESM6_ESM.zip › Figure4/4B/Blot_anti-HA_Marker.tif]

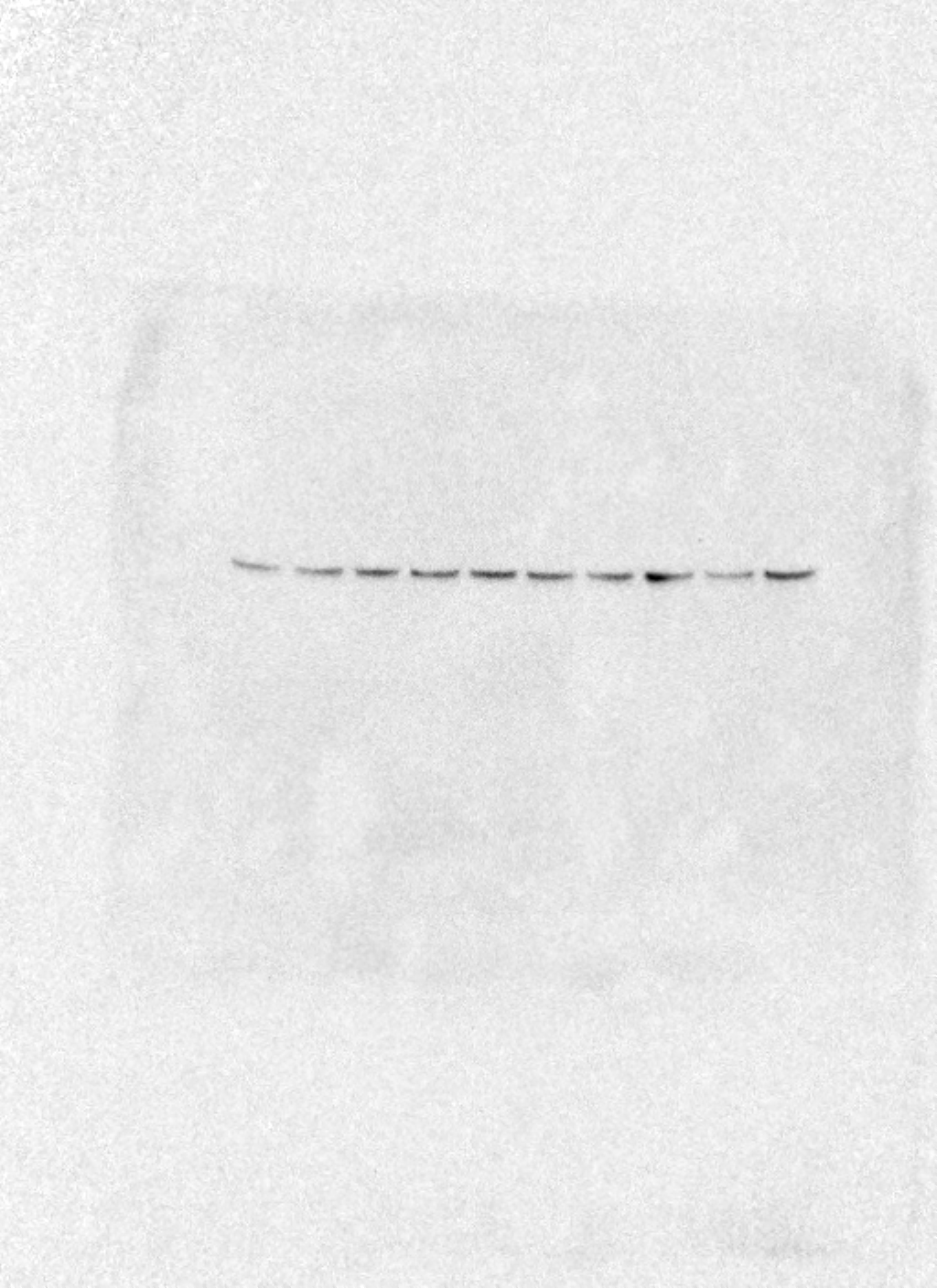

Supplement: Supplementary file 6 — Source data Fig. 4 [file 44318_2025_437_MOESM6_ESM.zip › Figure4/4B/Blot_aPgk1 .tif]

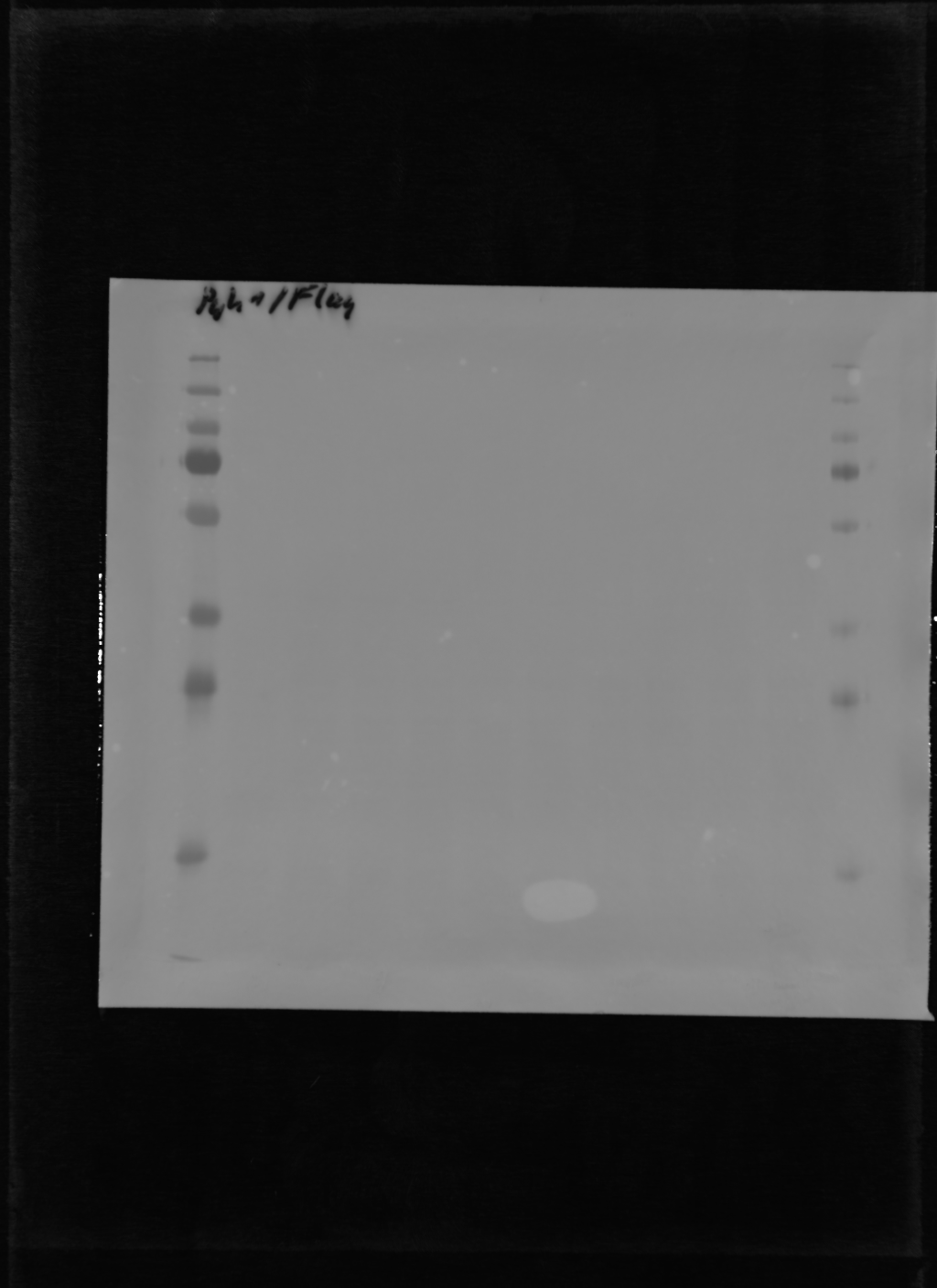

Supplement: Supplementary file 6 — Source data Fig. 4 [file 44318_2025_437_MOESM6_ESM.zip › Figure4/4B/Blot_aPgk1_Marker.tif]

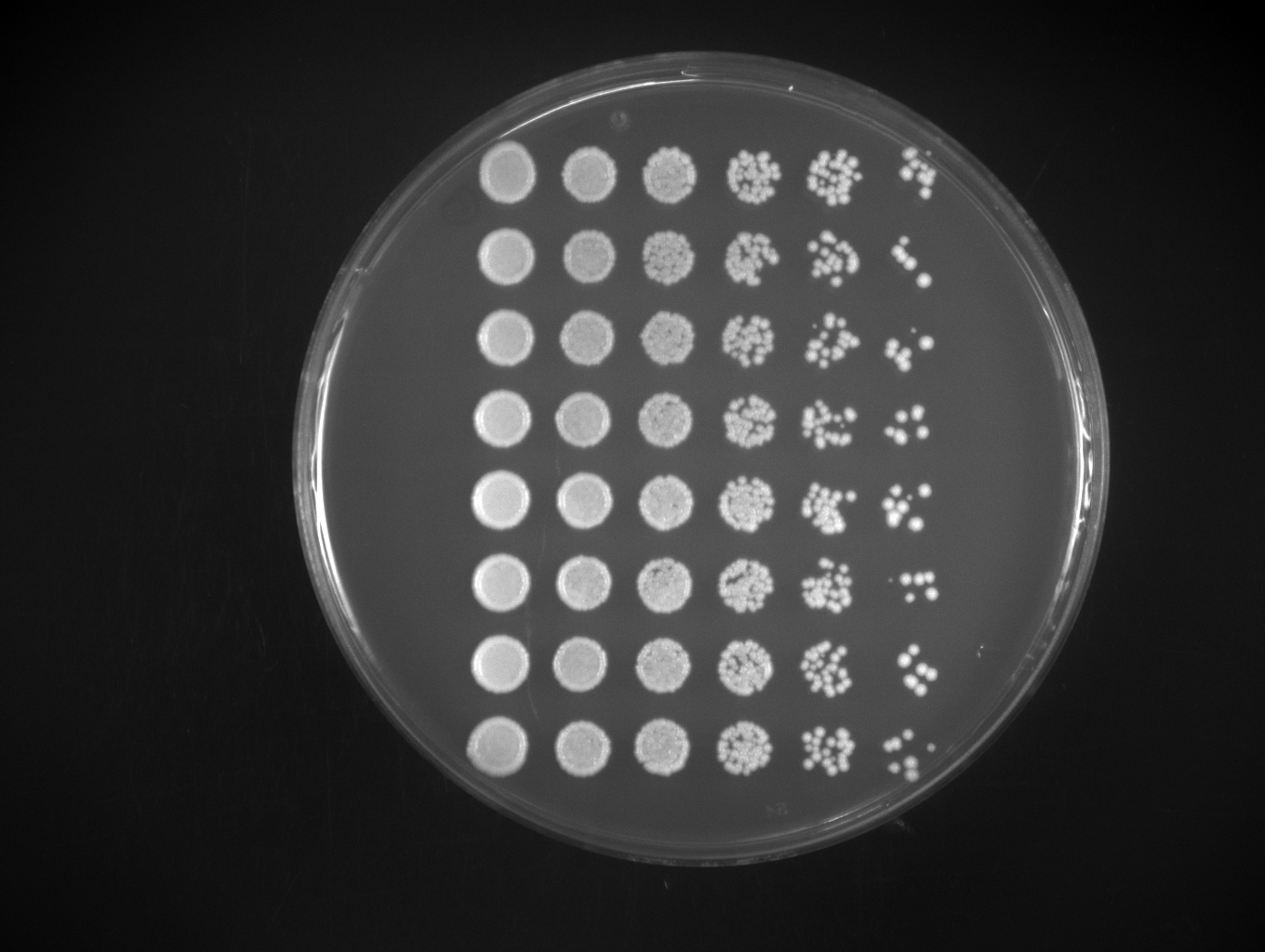

Supplement: Supplementary file 6 — Source data Fig. 4 [file 44318_2025_437_MOESM6_ESM.zip › Figure4/4C/WholeMount_lowerPlate_30C.tif]

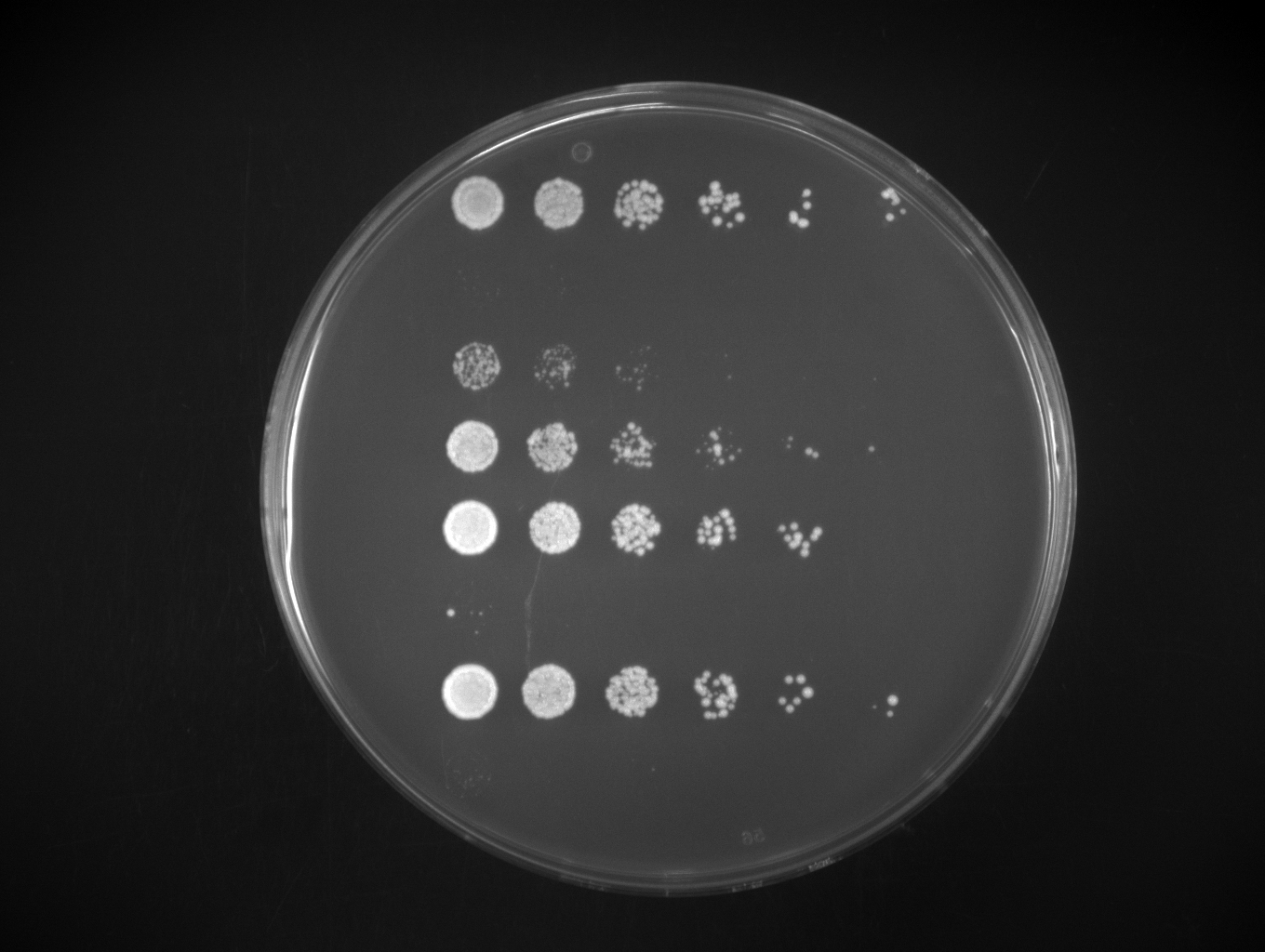

Supplement: Supplementary file 6 — Source data Fig. 4 [file 44318_2025_437_MOESM6_ESM.zip › Figure4/4C/WholeMount_lowerPlate_30C_benomyl.tif]

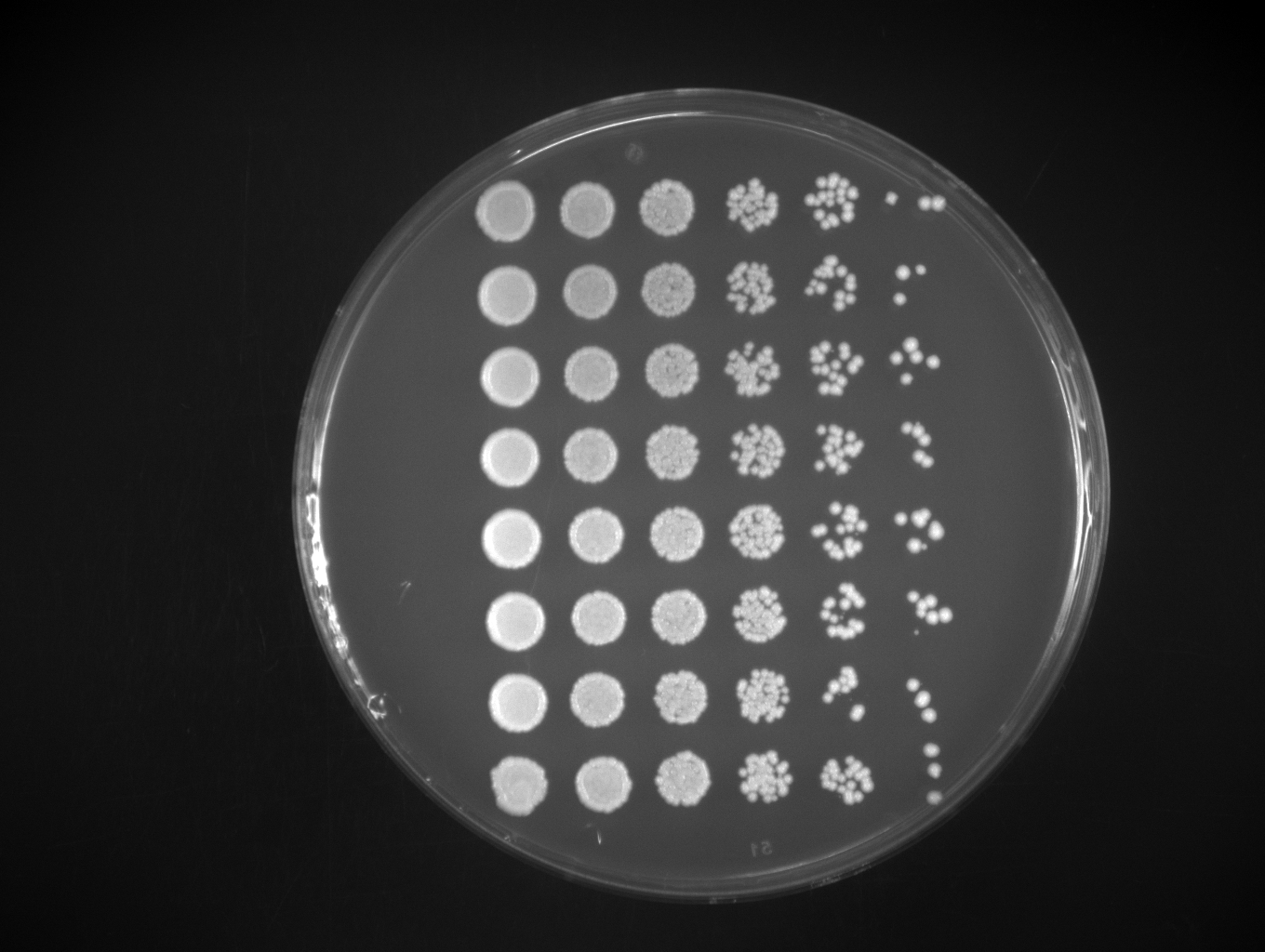

Supplement: Supplementary file 6 — Source data Fig. 4 [file 44318_2025_437_MOESM6_ESM.zip › Figure4/4C/WholeMount_upperPlate_30C.tif]

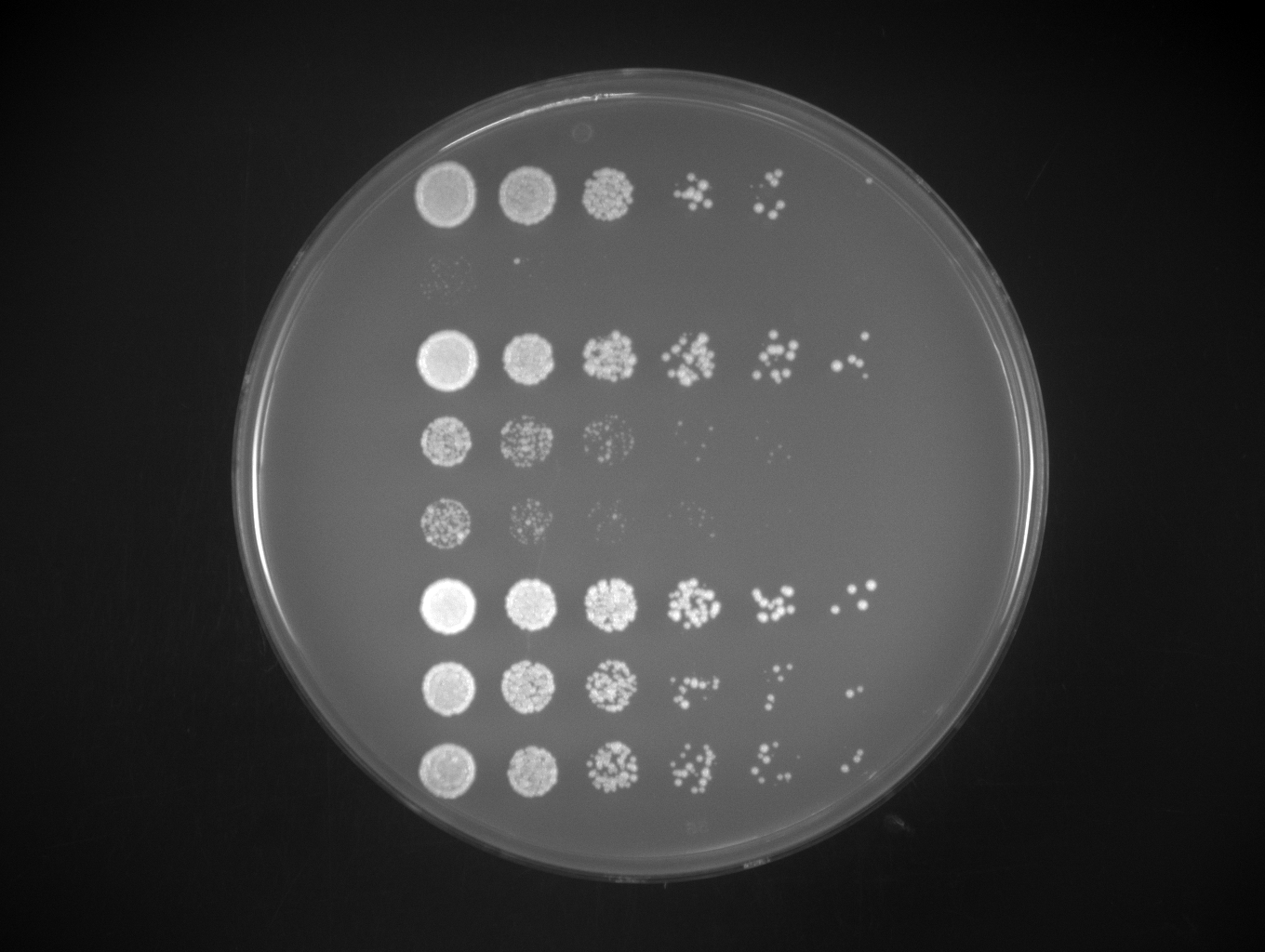

Supplement: Supplementary file 6 — Source data Fig. 4 [file 44318_2025_437_MOESM6_ESM.zip › Figure4/4C/WholeMount_upperPlate_30C_benomyl.tif]

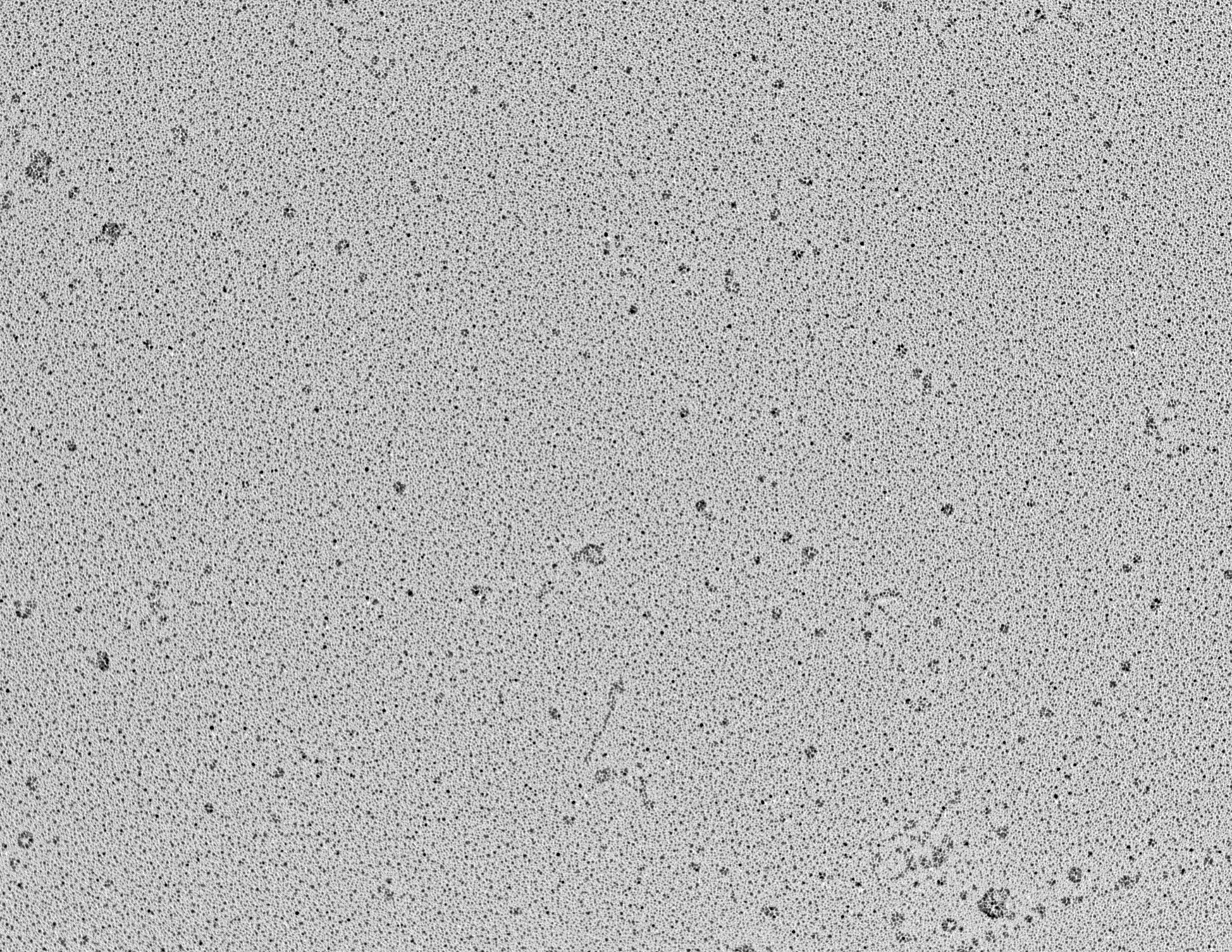

Supplement: Supplementary file 7 — Source data Fig. 5 [file 44318_2025_437_MOESM7_ESM.zip › Figure5/5B/76464.tif]

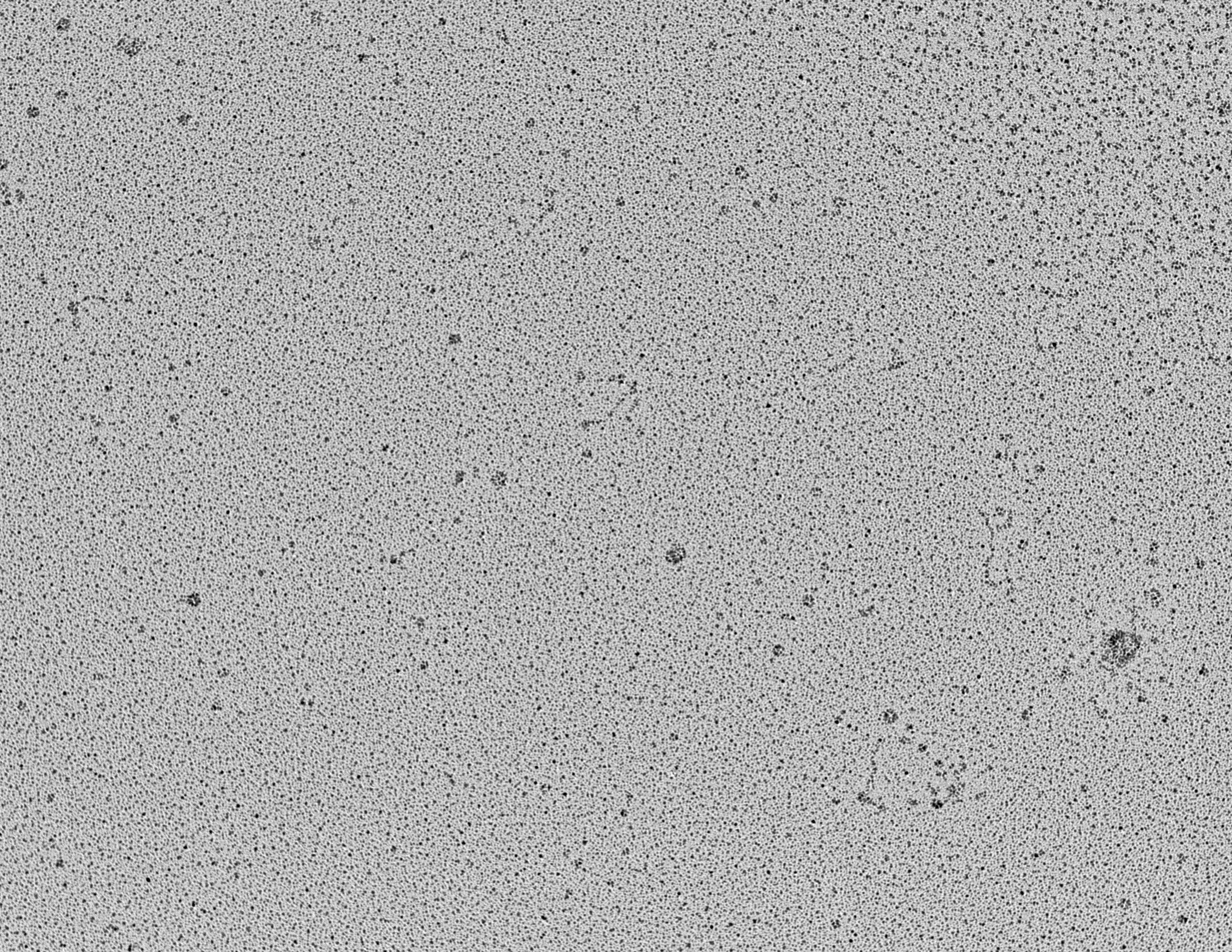

Supplement: Supplementary file 7 — Source data Fig. 5 [file 44318_2025_437_MOESM7_ESM.zip › Figure5/5B/76465.tif]

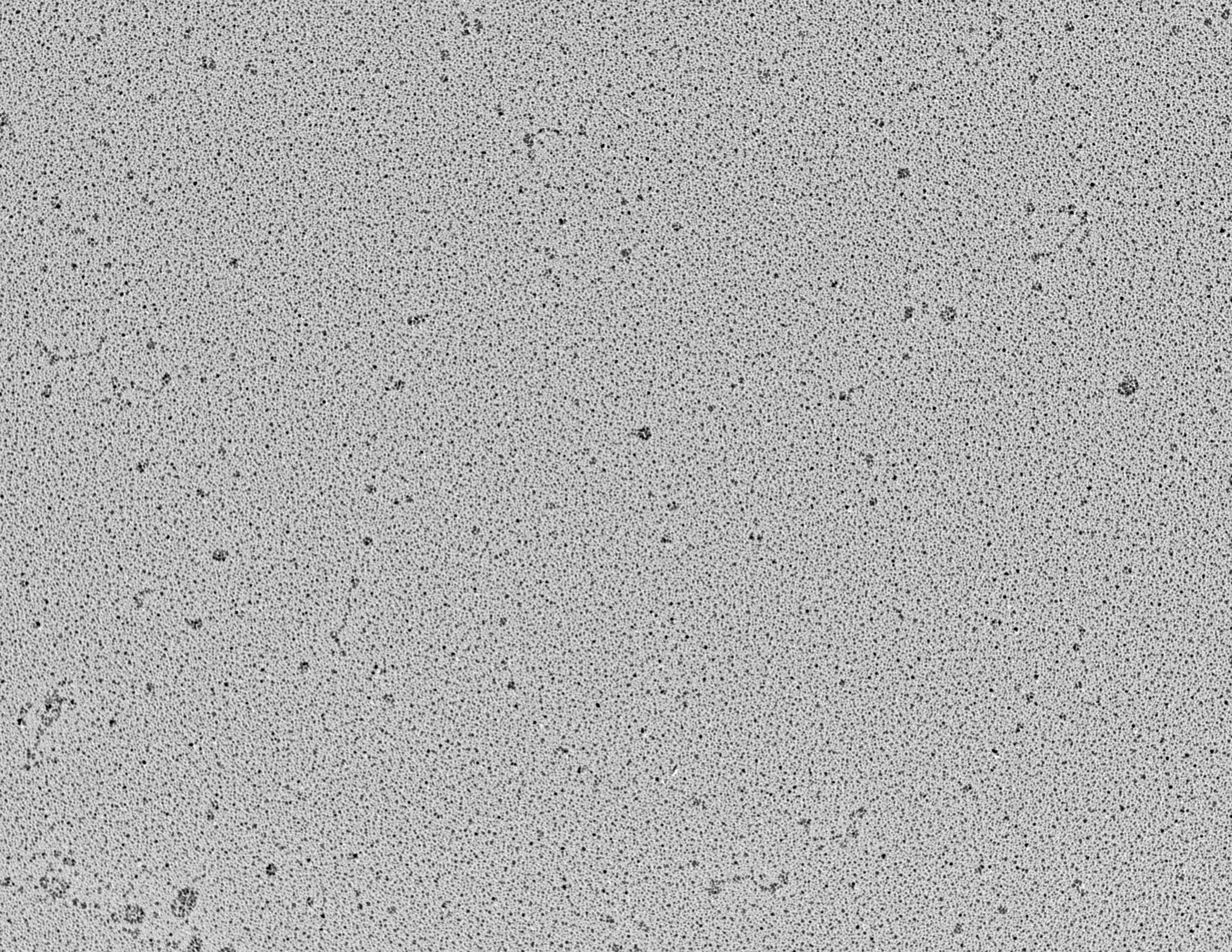

Supplement: Supplementary file 7 — Source data Fig. 5 [file 44318_2025_437_MOESM7_ESM.zip › Figure5/5B/76466.tif]

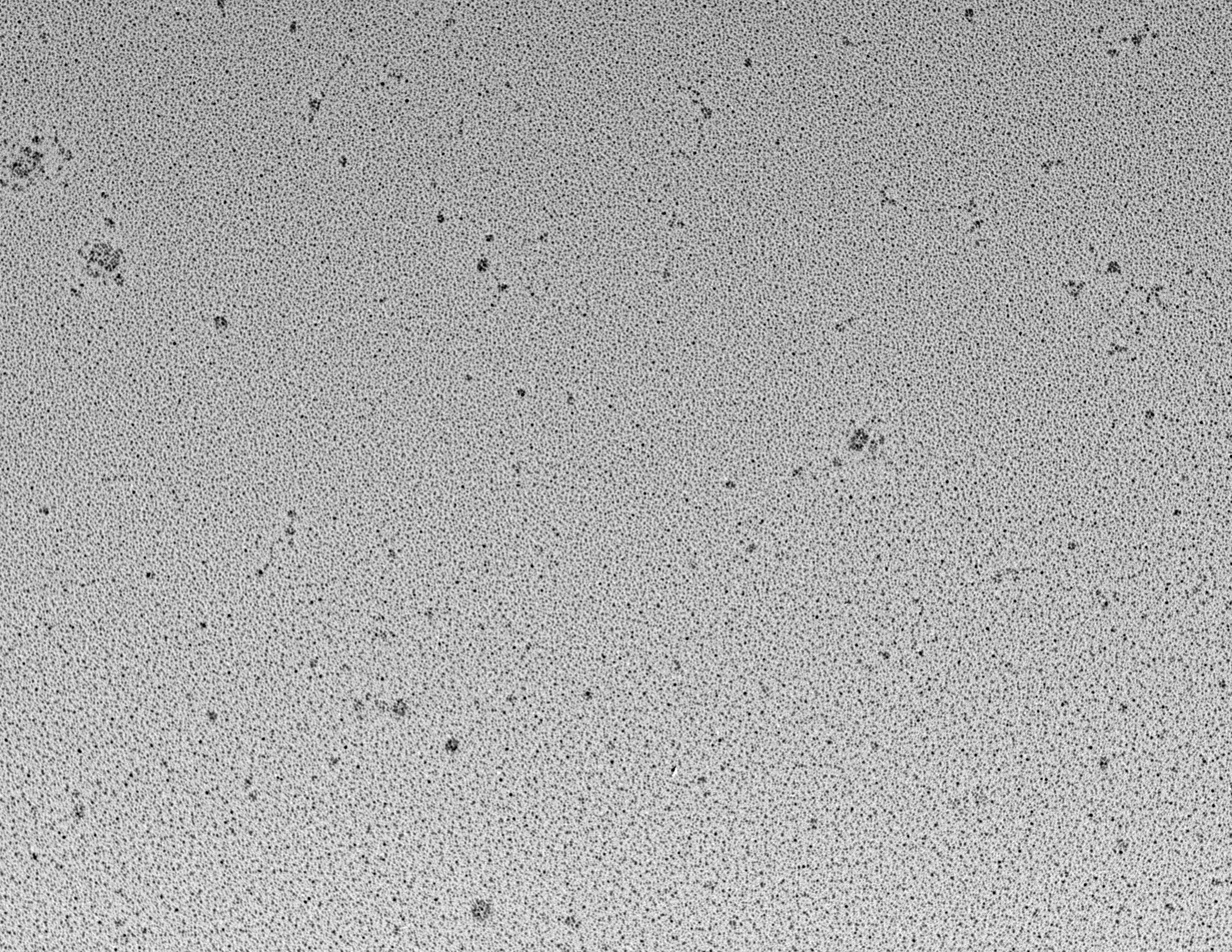

Supplement: Supplementary file 7 — Source data Fig. 5 [file 44318_2025_437_MOESM7_ESM.zip › Figure5/5B/76649.tif]

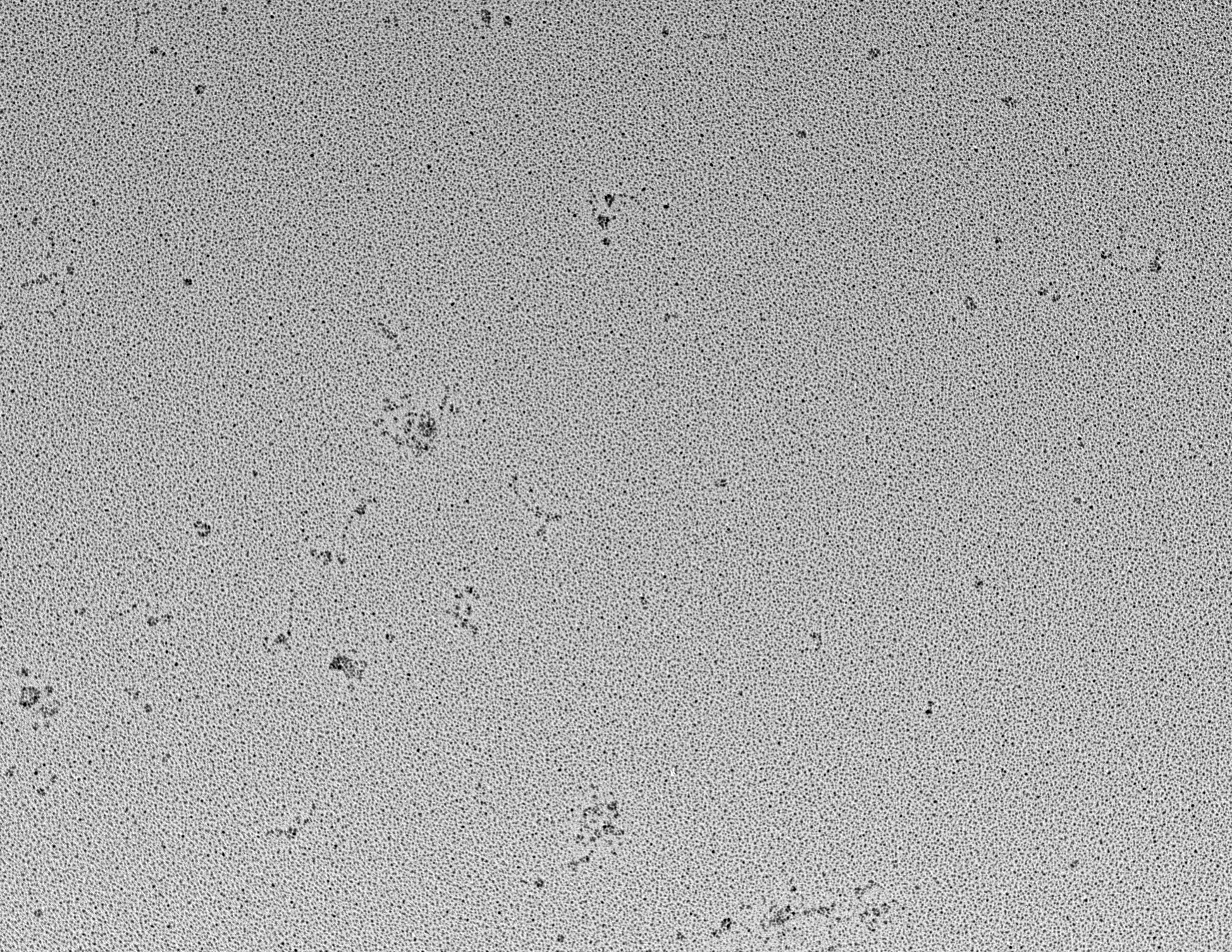

Supplement: Supplementary file 7 — Source data Fig. 5 [file 44318_2025_437_MOESM7_ESM.zip › Figure5/5B/76650.tif]

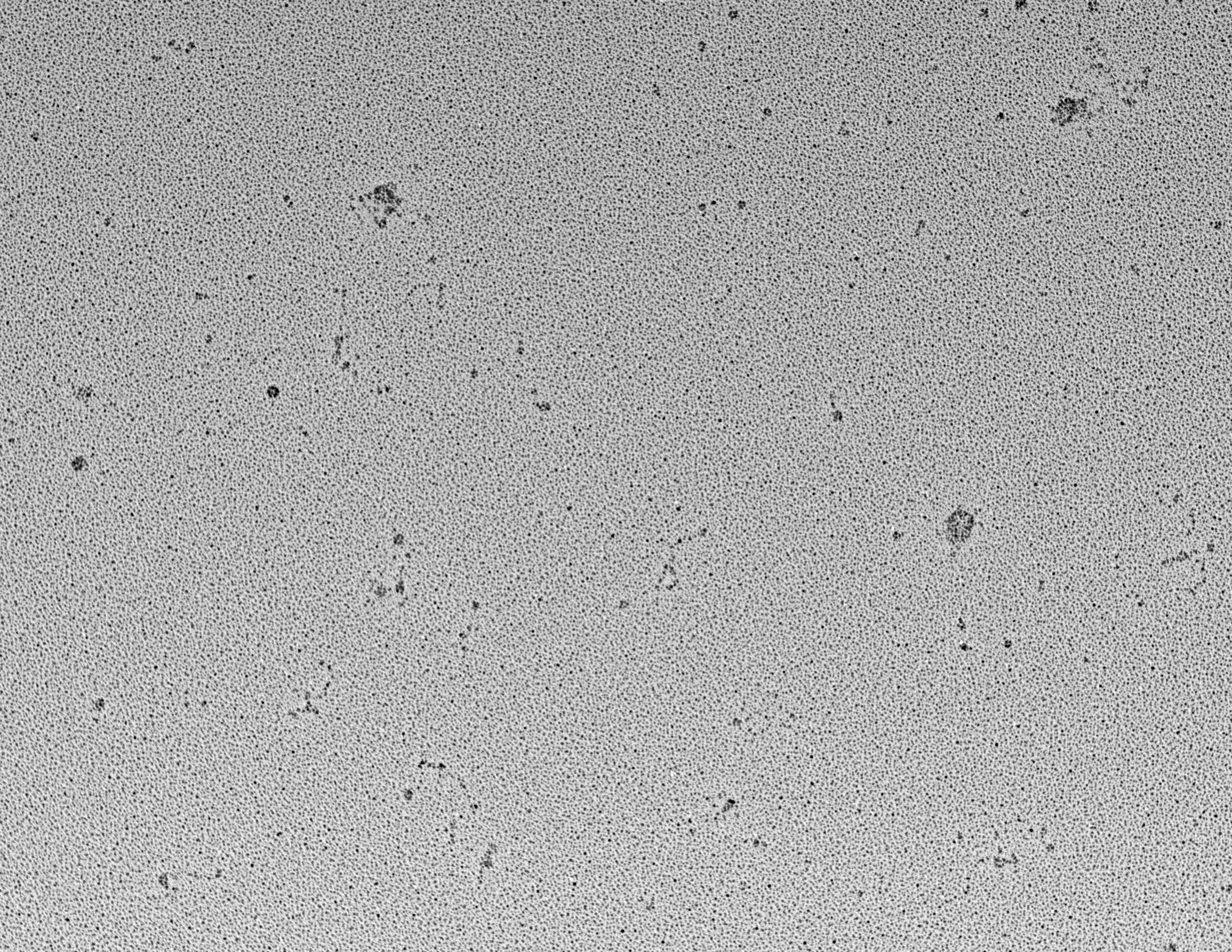

Supplement: Supplementary file 7 — Source data Fig. 5 [file 44318_2025_437_MOESM7_ESM.zip › Figure5/5B/76651.tif]

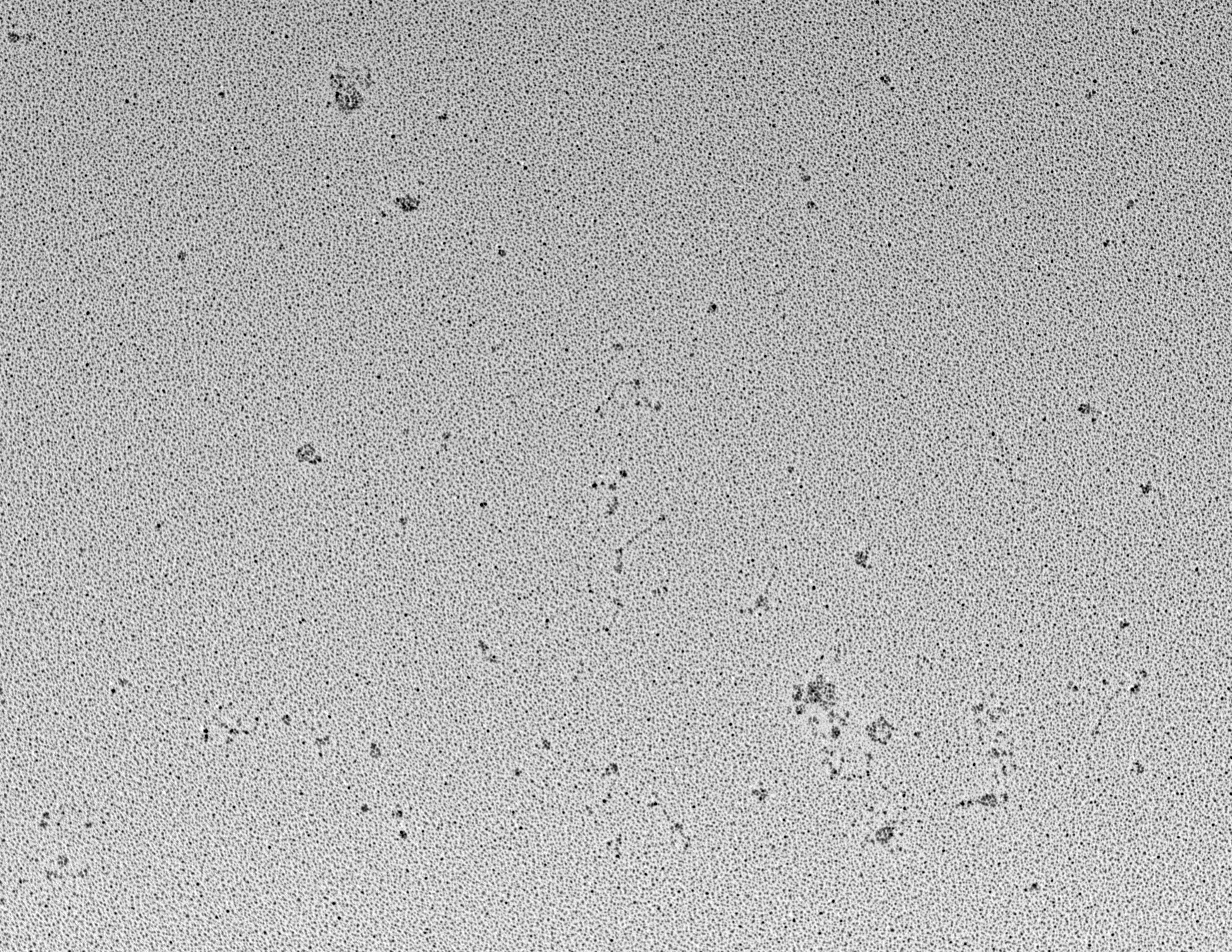

Supplement: Supplementary file 7 — Source data Fig. 5 [file 44318_2025_437_MOESM7_ESM.zip › Figure5/5B/76652.tif]

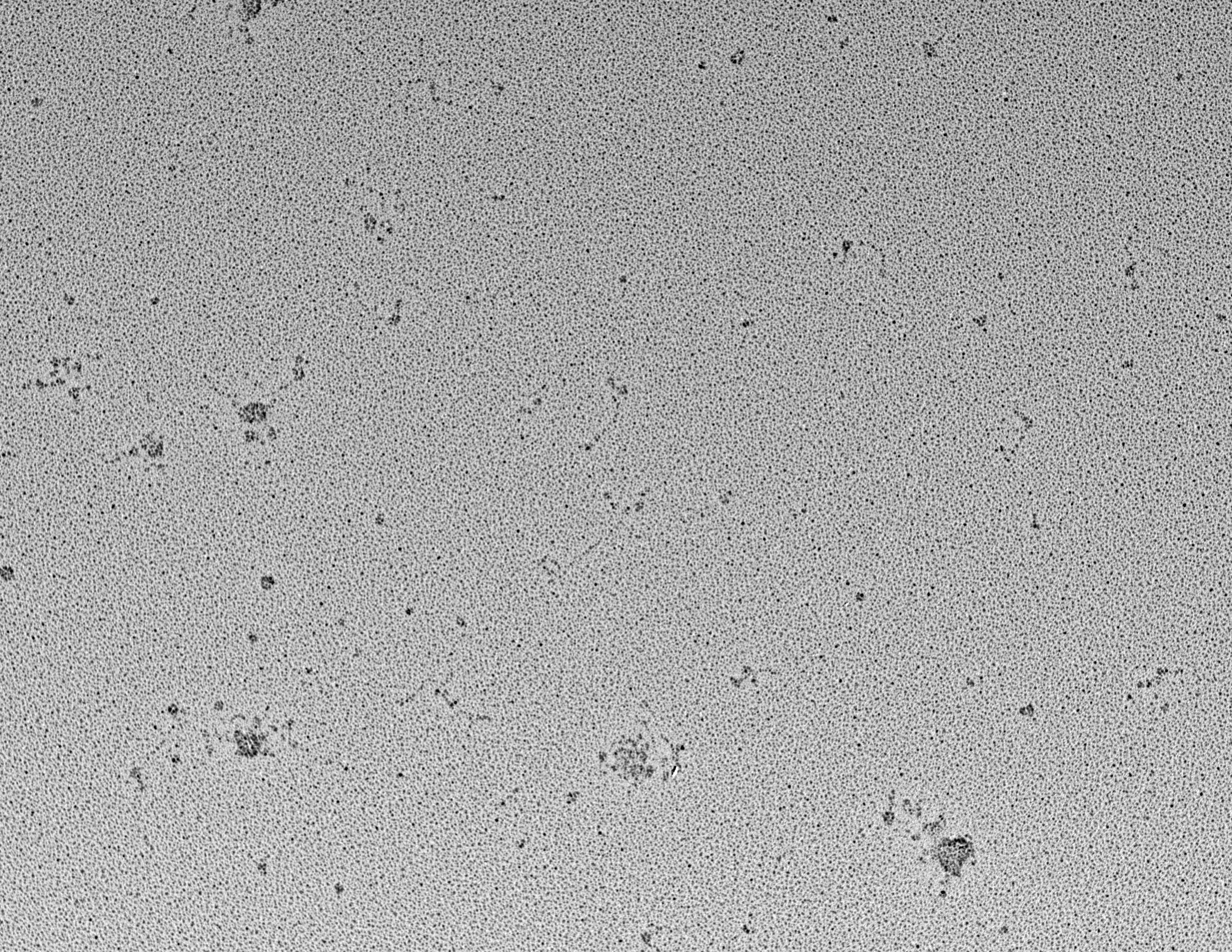

Supplement: Supplementary file 7 — Source data Fig. 5 [file 44318_2025_437_MOESM7_ESM.zip › Figure5/5B/76653.tif]

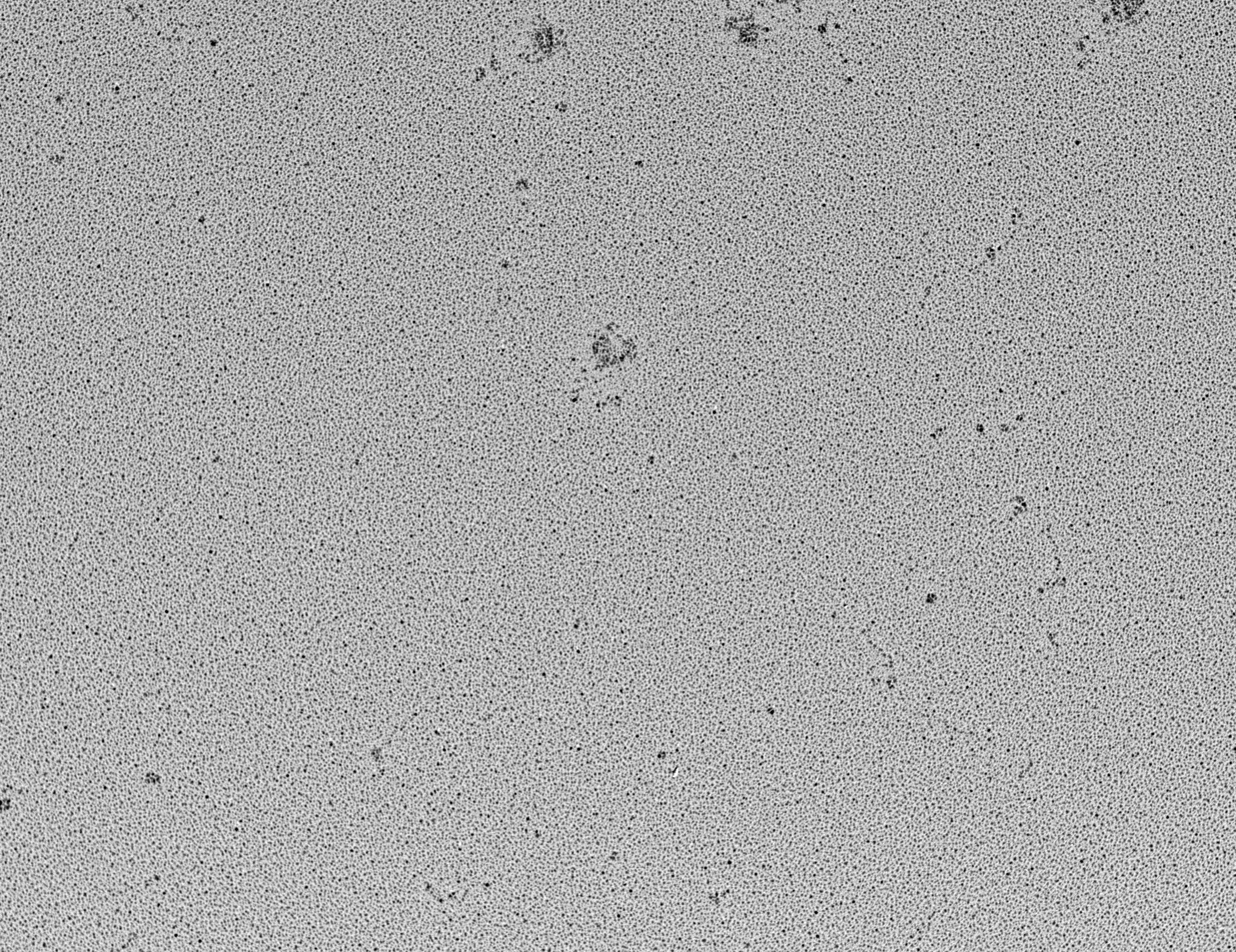

Supplement: Supplementary file 7 — Source data Fig. 5 [file 44318_2025_437_MOESM7_ESM.zip › Figure5/5B/76654.tif]

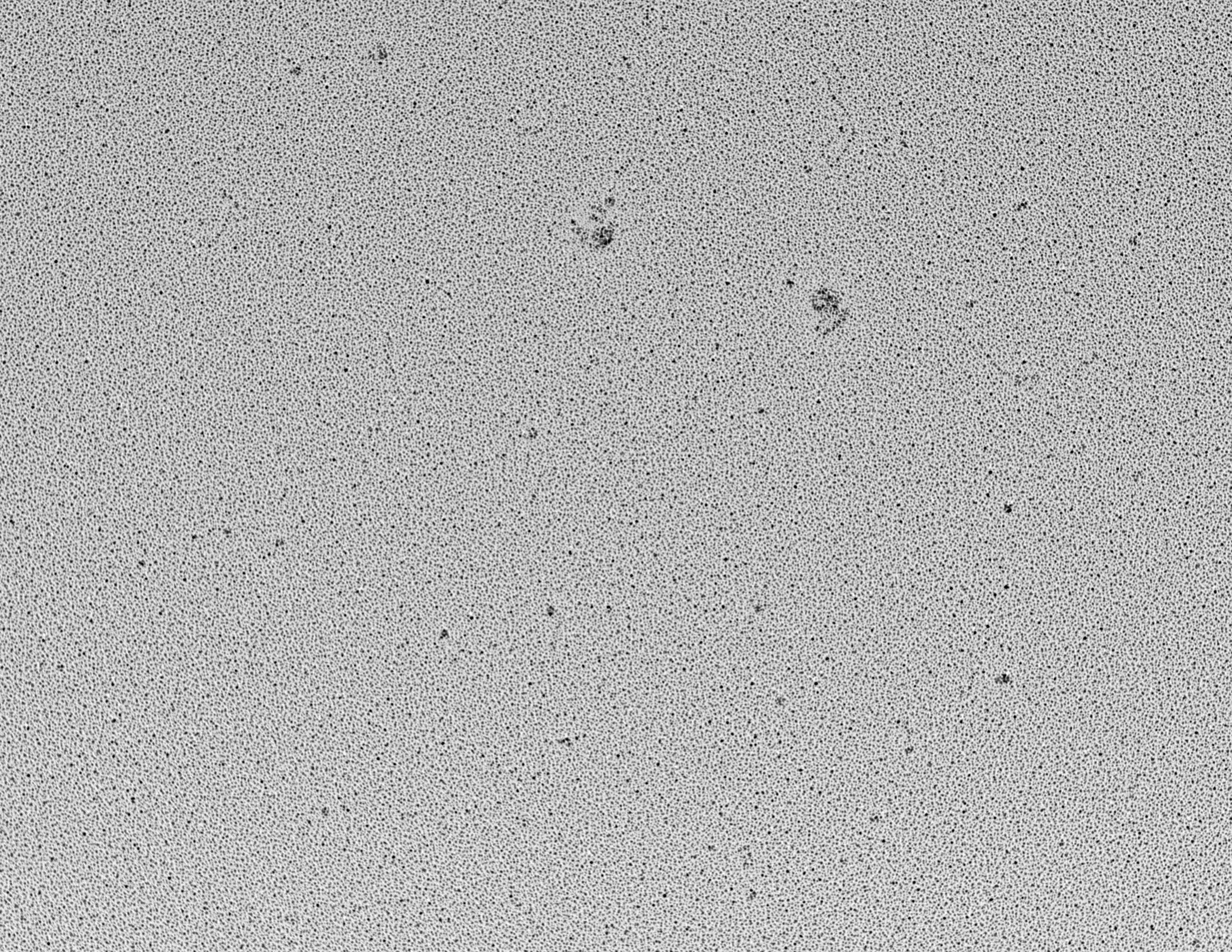

Supplement: Supplementary file 7 — Source data Fig. 5 [file 44318_2025_437_MOESM7_ESM.zip › Figure5/5B/76655.tif]

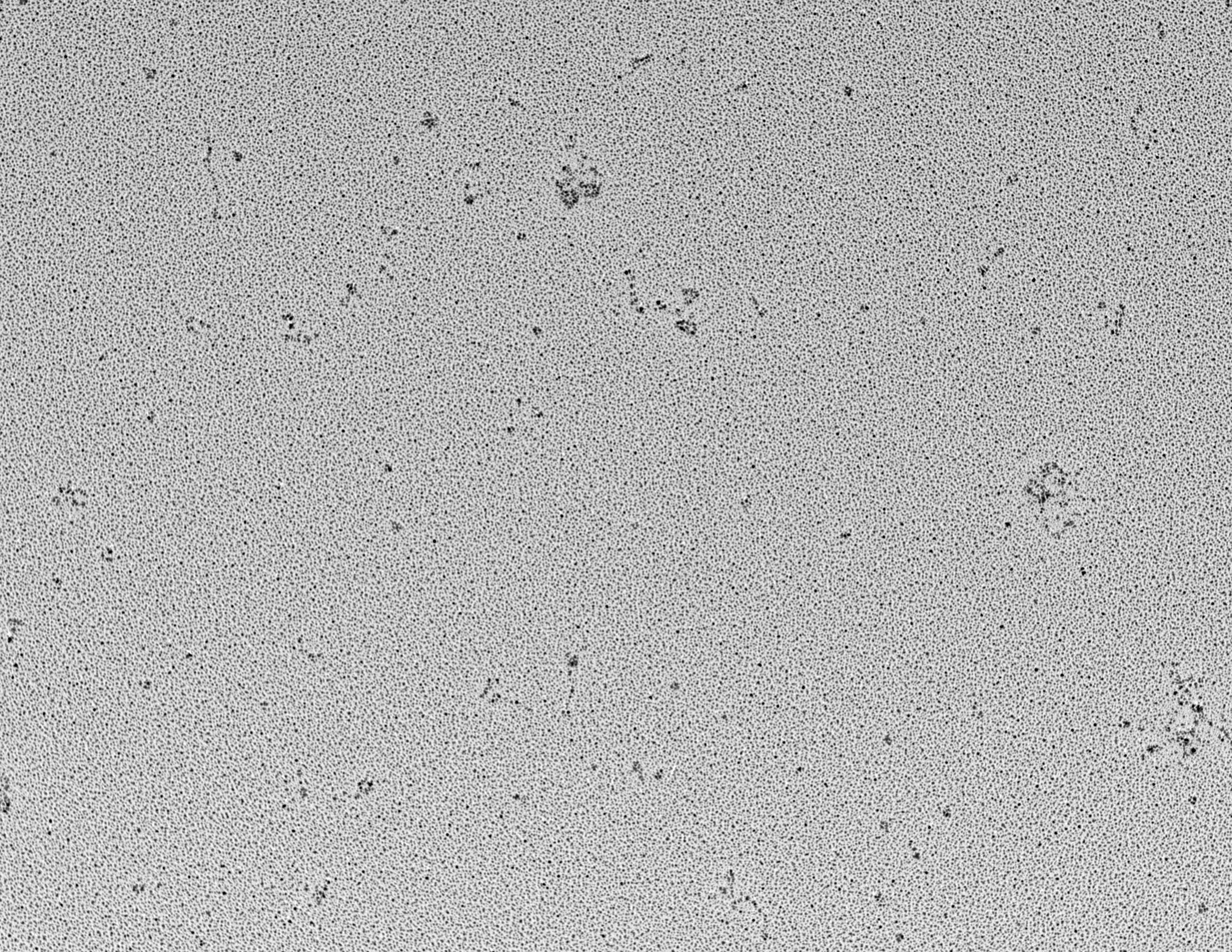

Supplement: Supplementary file 7 — Source data Fig. 5 [file 44318_2025_437_MOESM7_ESM.zip › Figure5/5B/76656.tif]

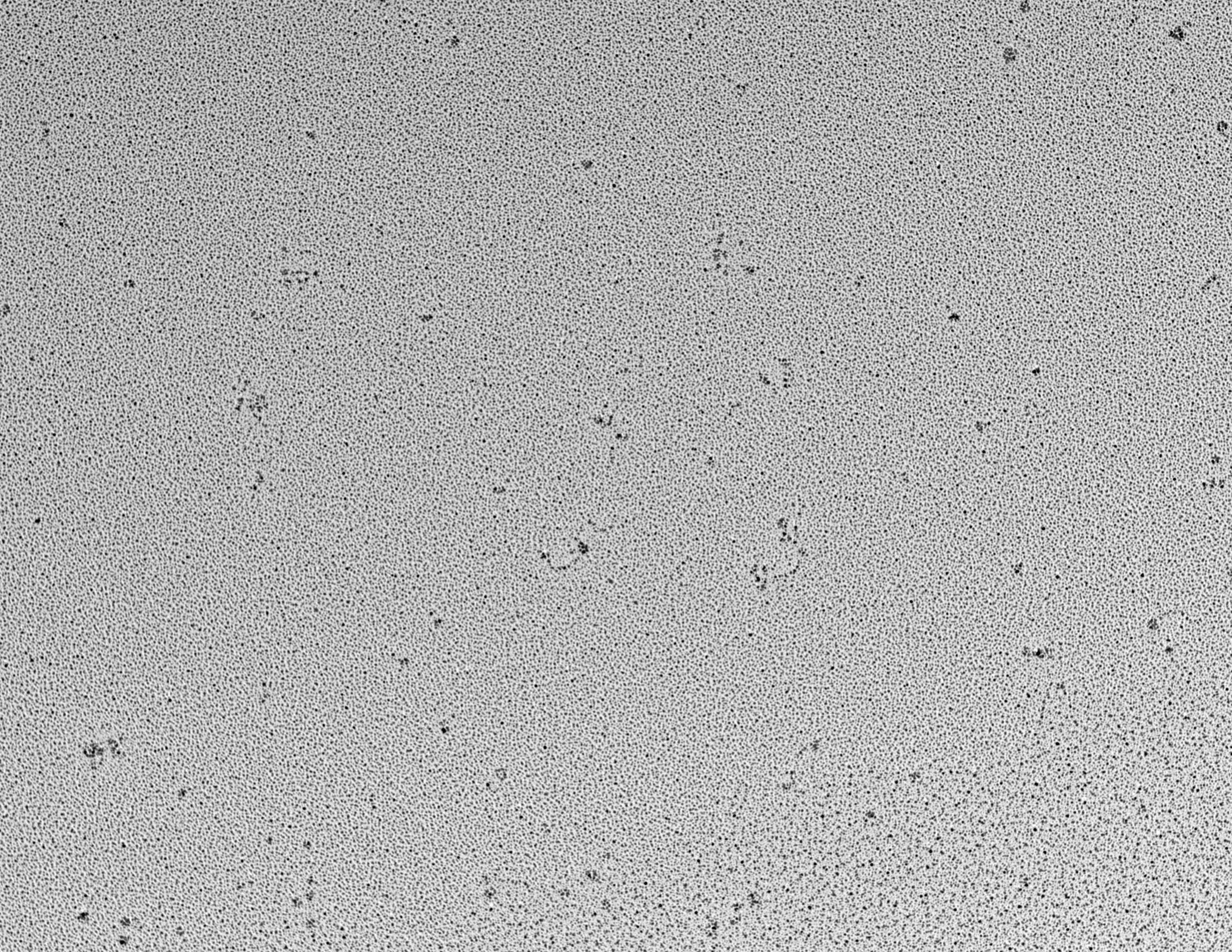

Supplement: Supplementary file 7 — Source data Fig. 5 [file 44318_2025_437_MOESM7_ESM.zip › Figure5/5B/76657.tif]

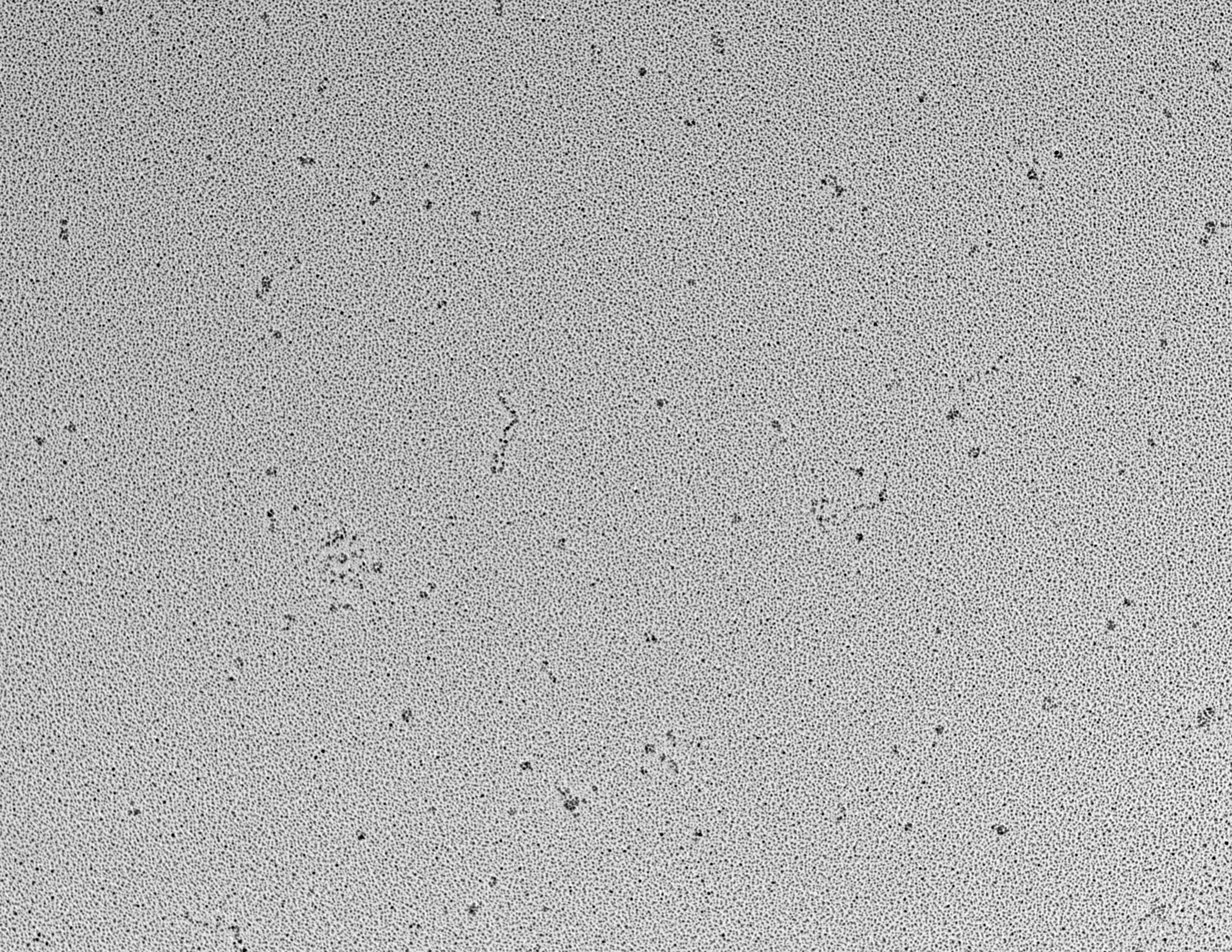

Supplement: Supplementary file 7 — Source data Fig. 5 [file 44318_2025_437_MOESM7_ESM.zip › Figure5/5B/76658.tif]

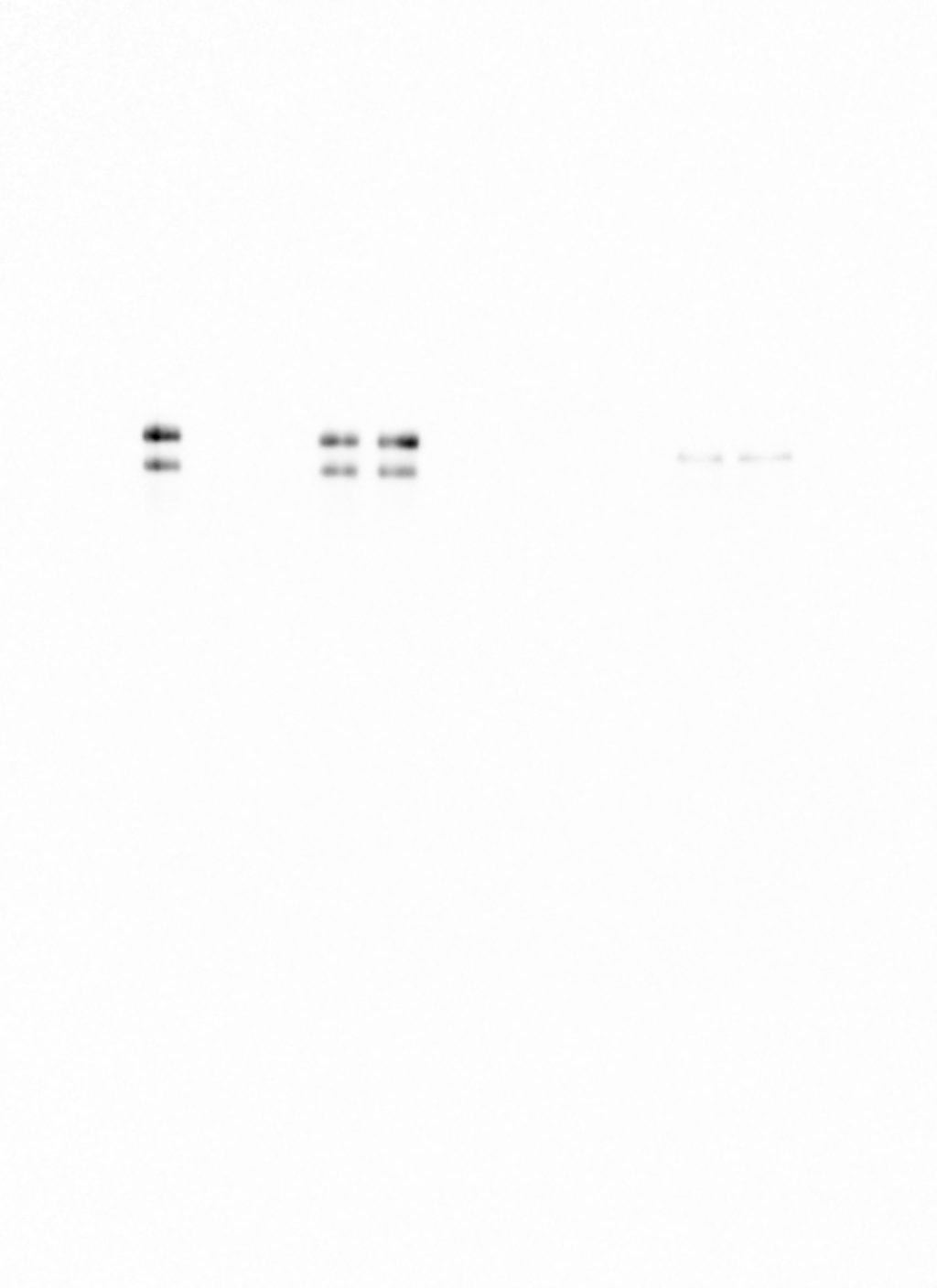

Supplement: Supplementary file 7 — Source data Fig. 5 [file 44318_2025_437_MOESM7_ESM.zip › Figure5/5C/Blot_His.tif]

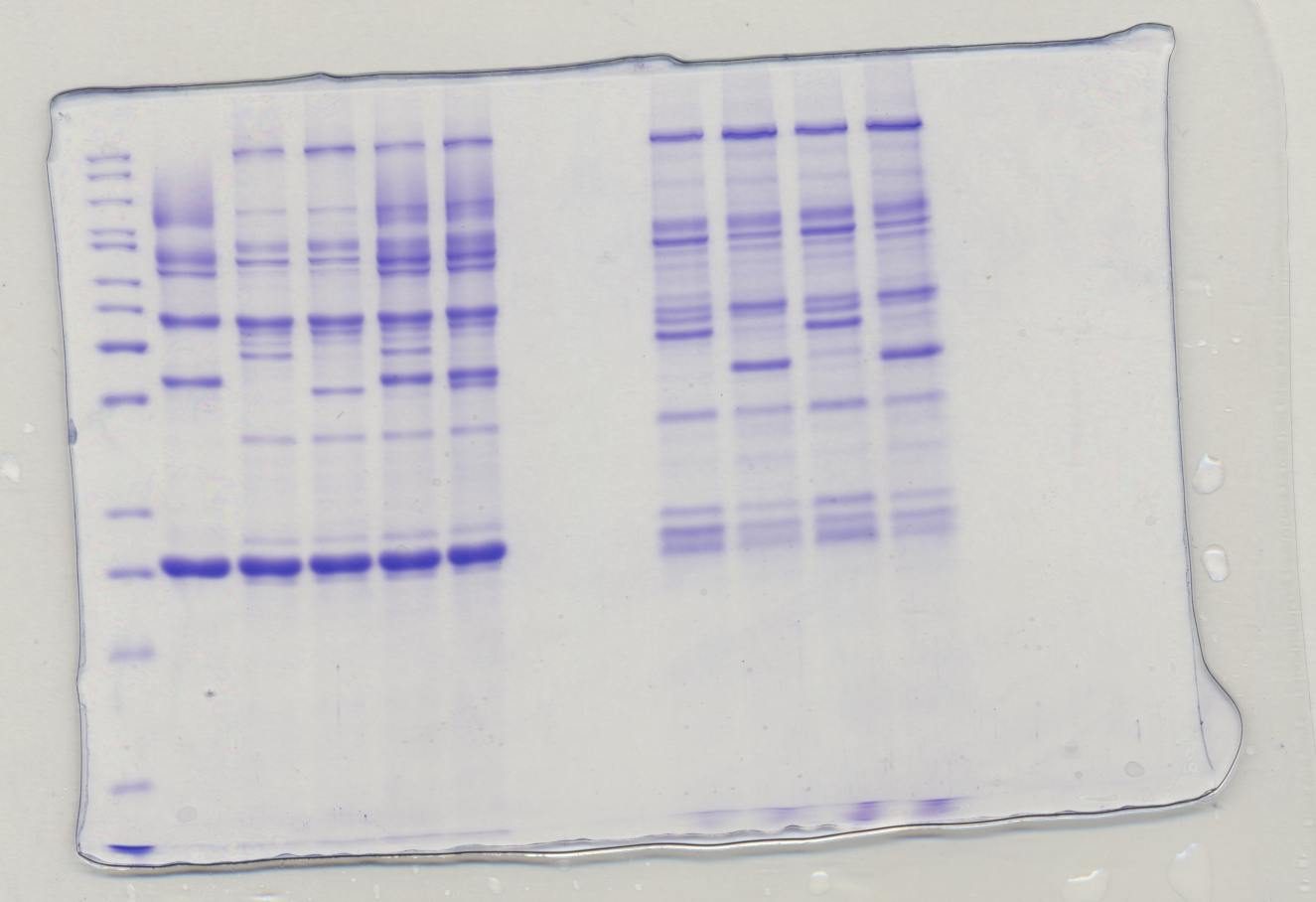

Supplement: Supplementary file 7 — Source data Fig. 5 [file 44318_2025_437_MOESM7_ESM.zip › Figure5/5C/Gel_Coomassie.tif]

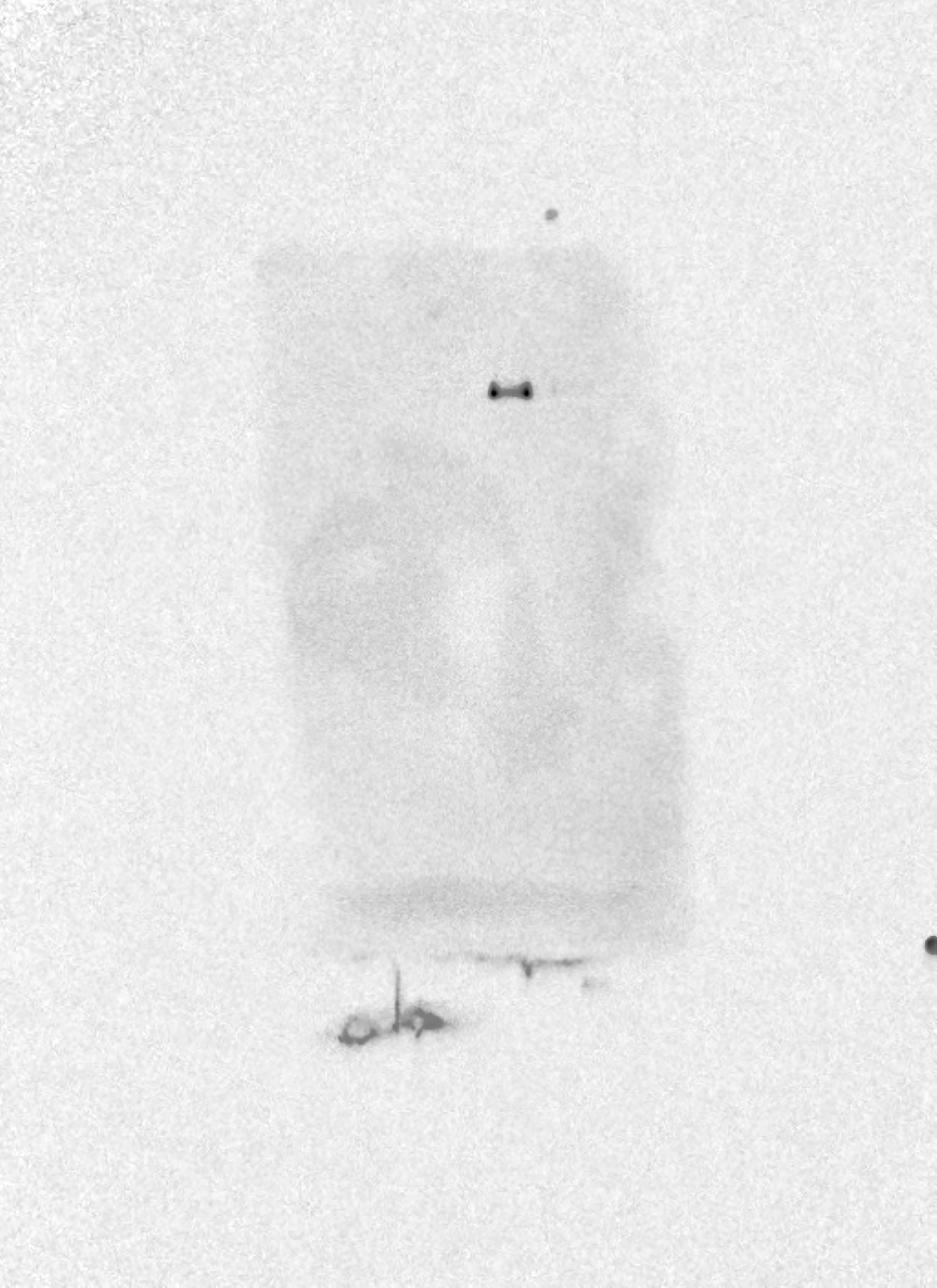

Supplement: Supplementary file 7 — Source data Fig. 5 [file 44318_2025_437_MOESM7_ESM.zip › Figure5/5D/Blot_His_elution.tif]

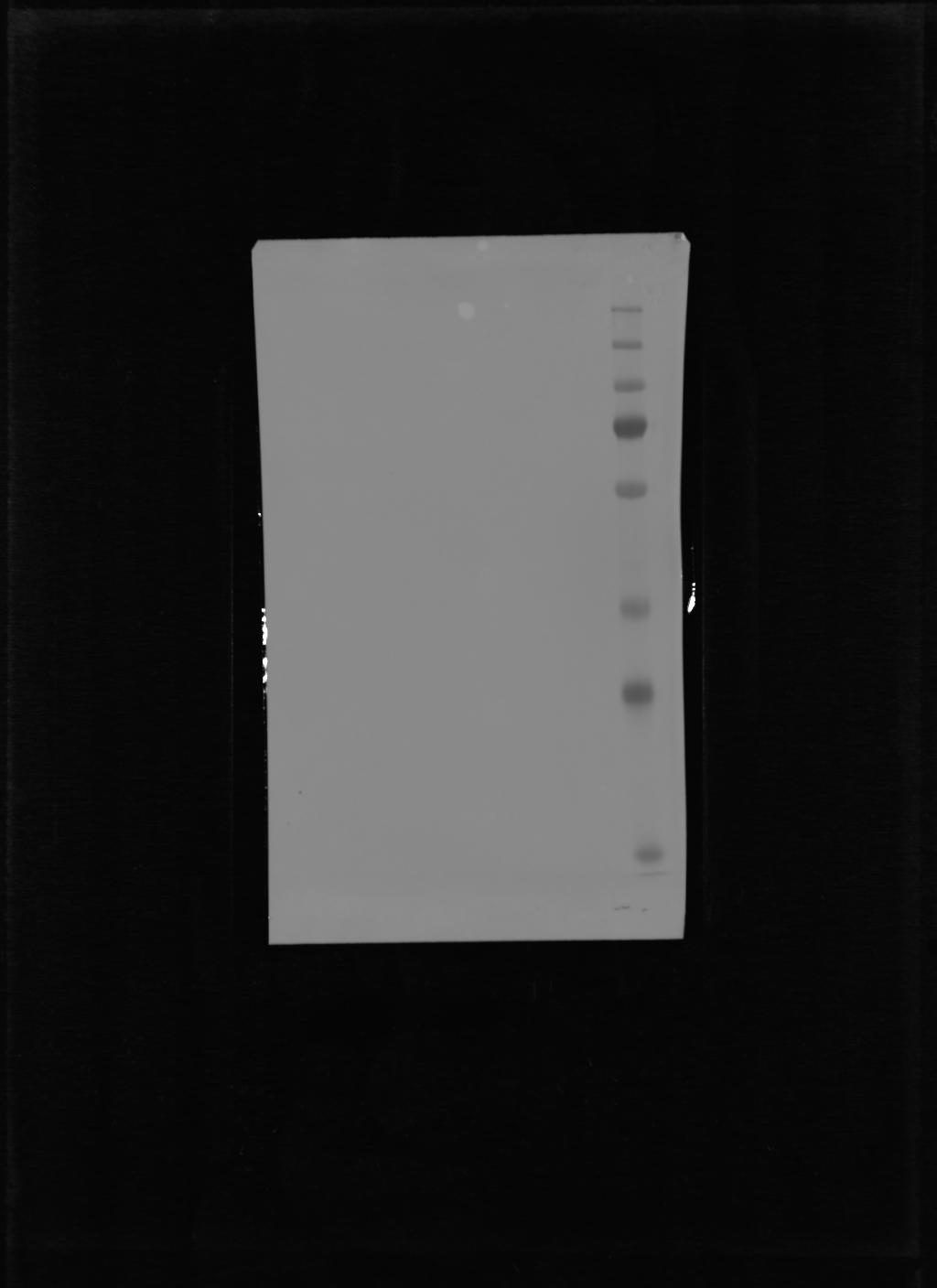

Supplement: Supplementary file 7 — Source data Fig. 5 [file 44318_2025_437_MOESM7_ESM.zip › Figure5/5D/Blot_His_elution_marker.tif]

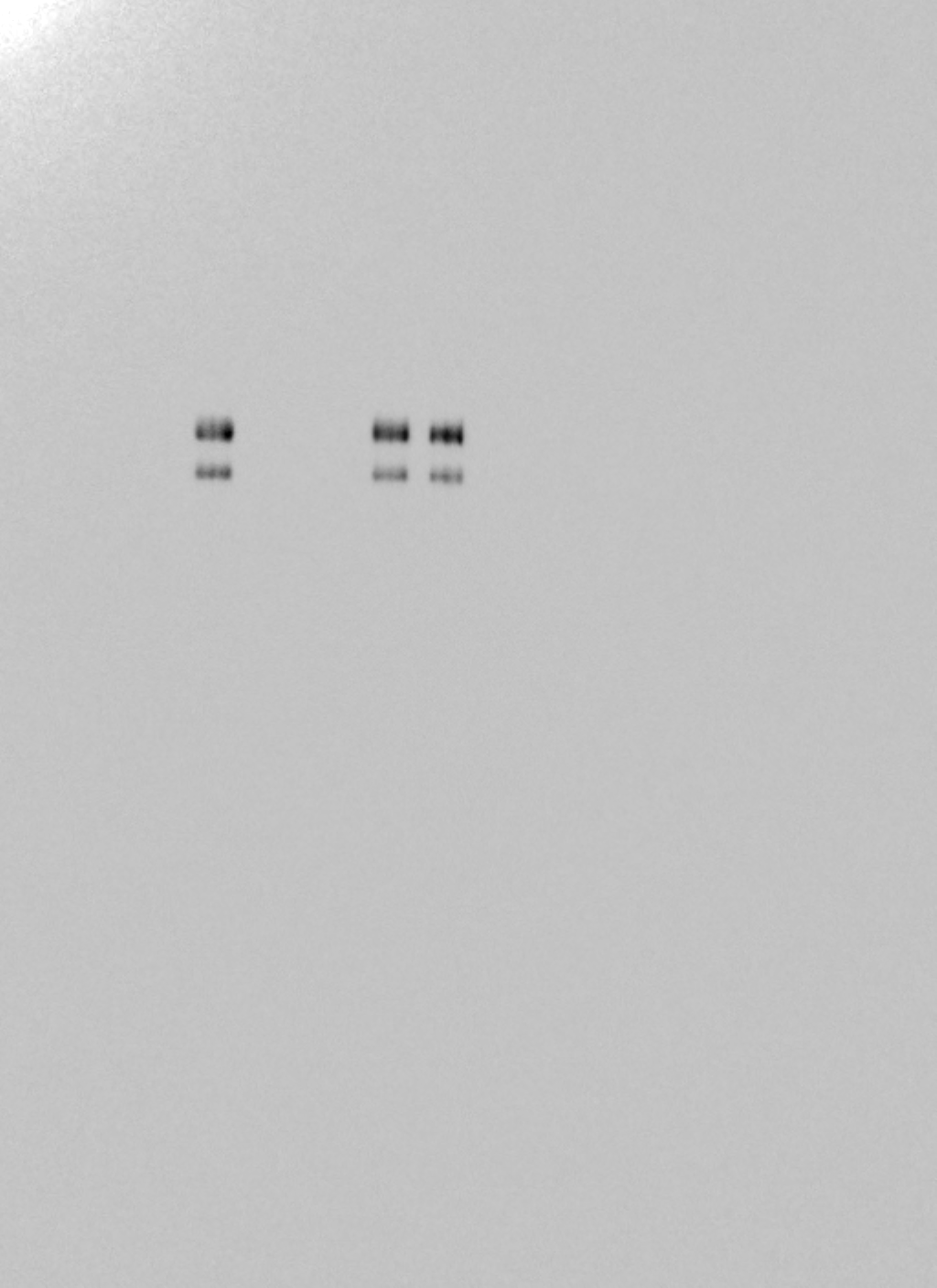

Supplement: Supplementary file 7 — Source data Fig. 5 [file 44318_2025_437_MOESM7_ESM.zip › Figure5/5D/Blot_His_input.tif]

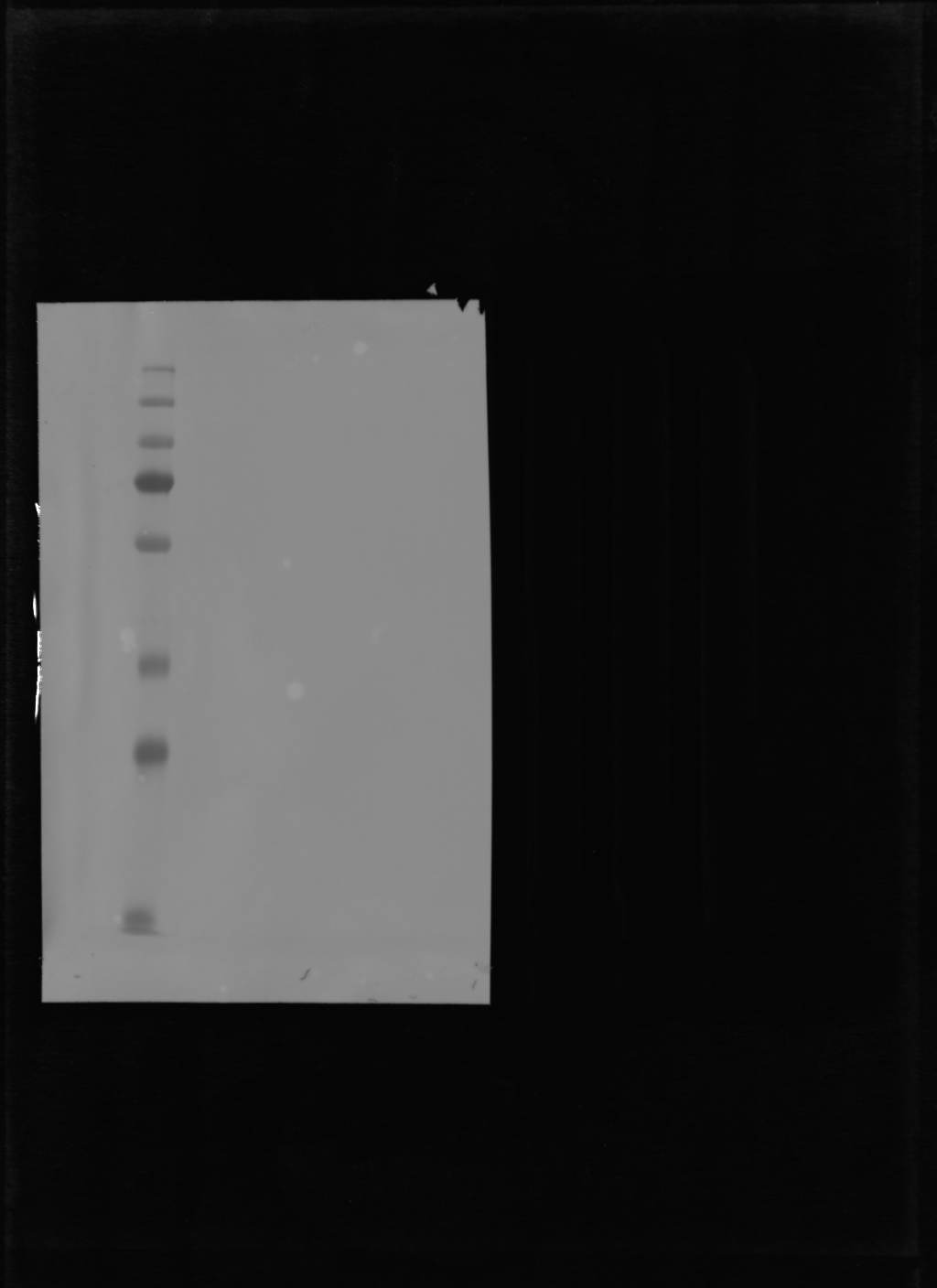

Supplement: Supplementary file 7 — Source data Fig. 5 [file 44318_2025_437_MOESM7_ESM.zip › Figure5/5D/Blot_His_input_marker.tif]

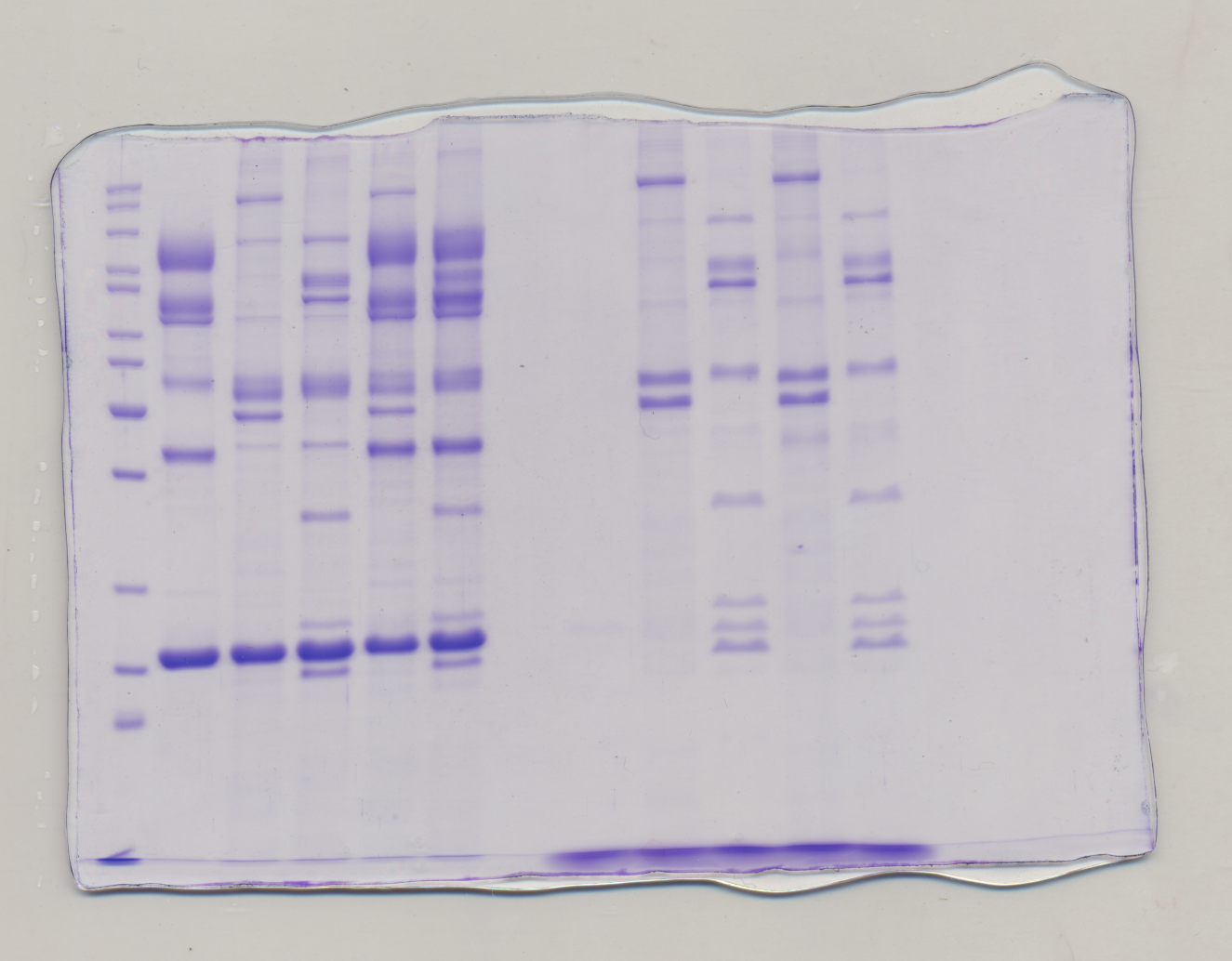

Supplement: Supplementary file 7 — Source data Fig. 5 [file 44318_2025_437_MOESM7_ESM.zip › Figure5/5D/Gel_Coomassie.tif]

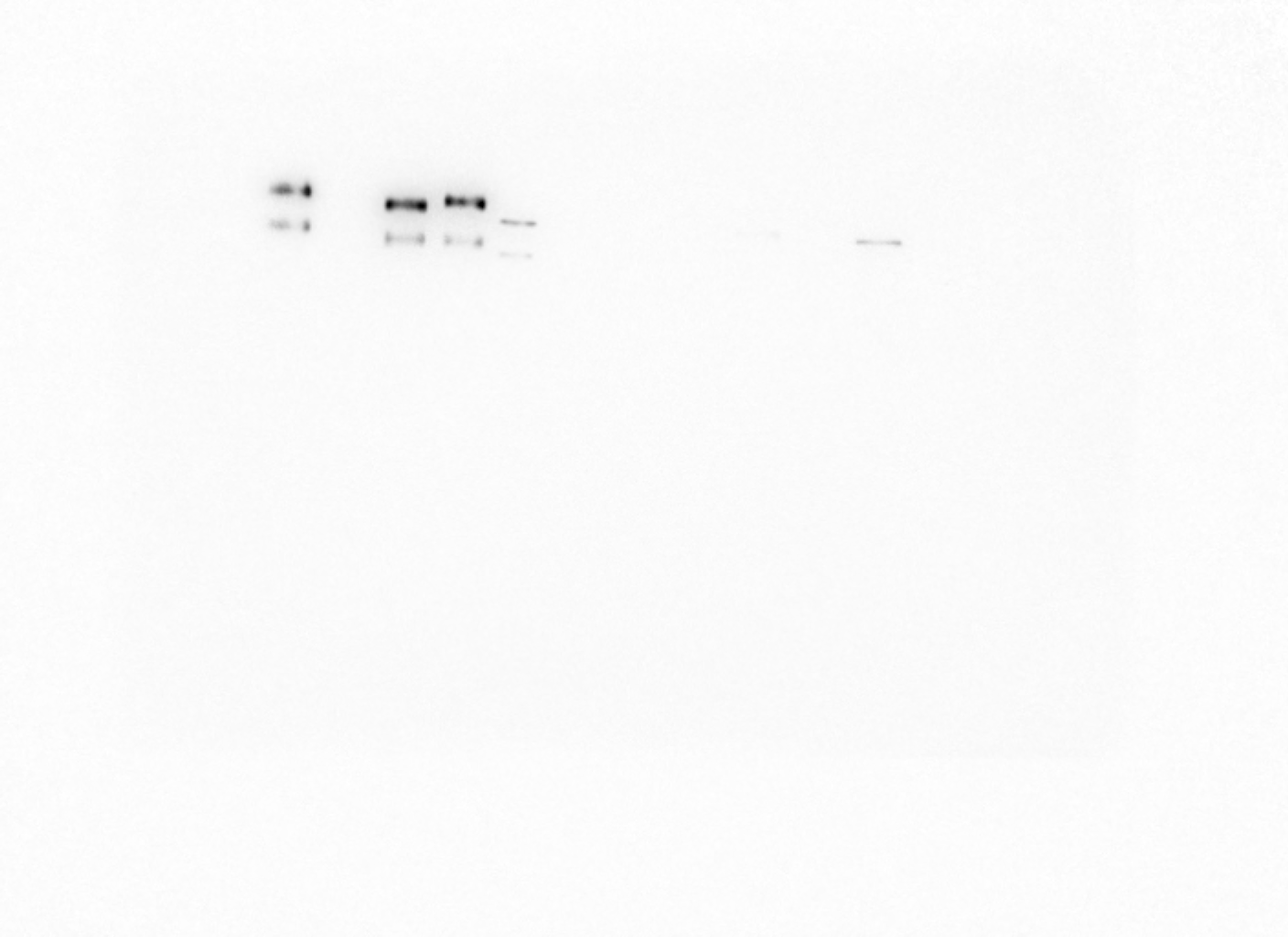

Supplement: Supplementary file 7 — Source data Fig. 5 [file 44318_2025_437_MOESM7_ESM.zip › Figure5/5E/Blot_His.tif]

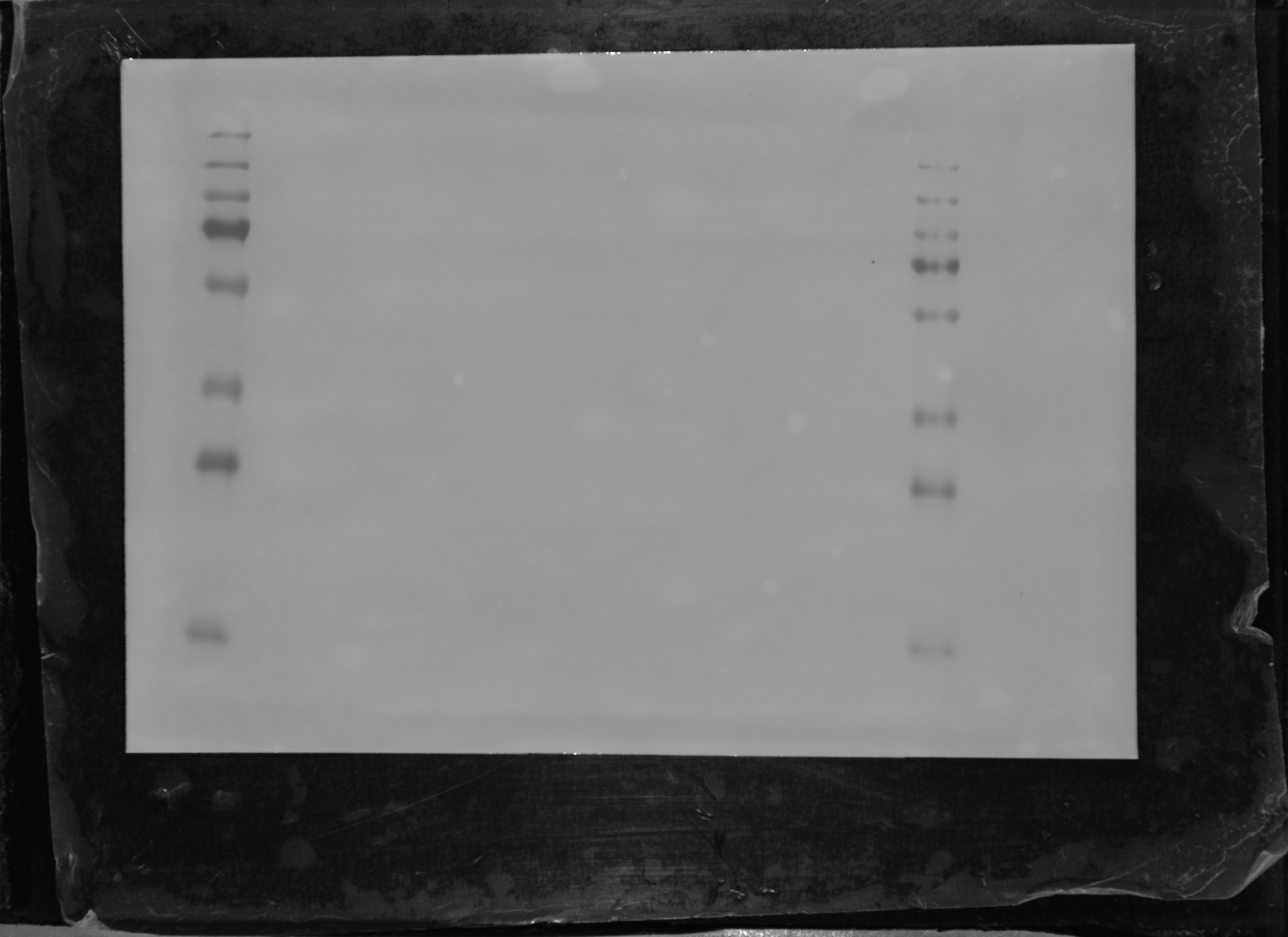

Supplement: Supplementary file 7 — Source data Fig. 5 [file 44318_2025_437_MOESM7_ESM.zip › Figure5/5E/Blot_His_marker.tif]

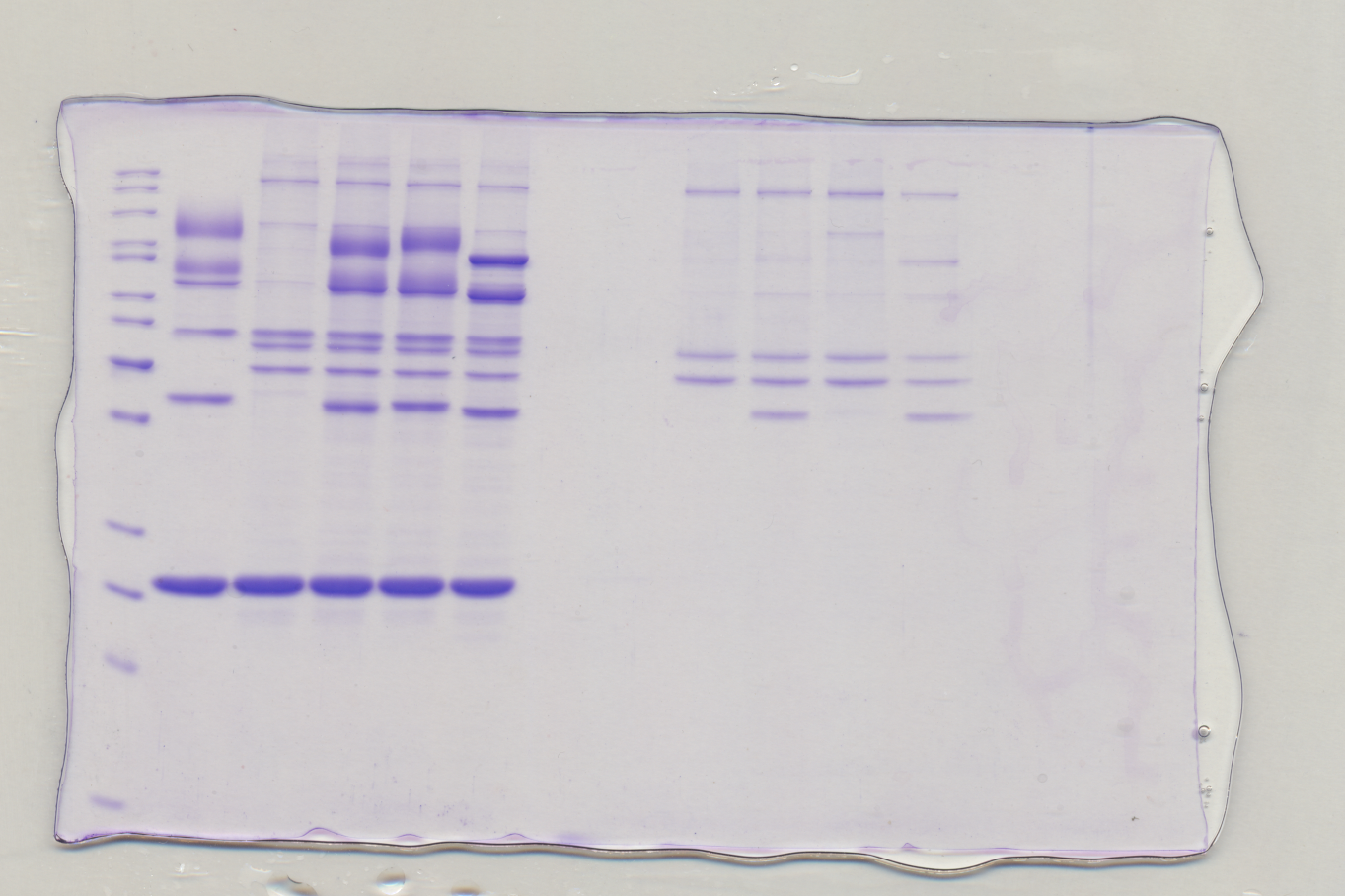

Supplement: Supplementary file 7 — Source data Fig. 5 [file 44318_2025_437_MOESM7_ESM.zip › Figure5/5E/Gel_Coomassie.tif]

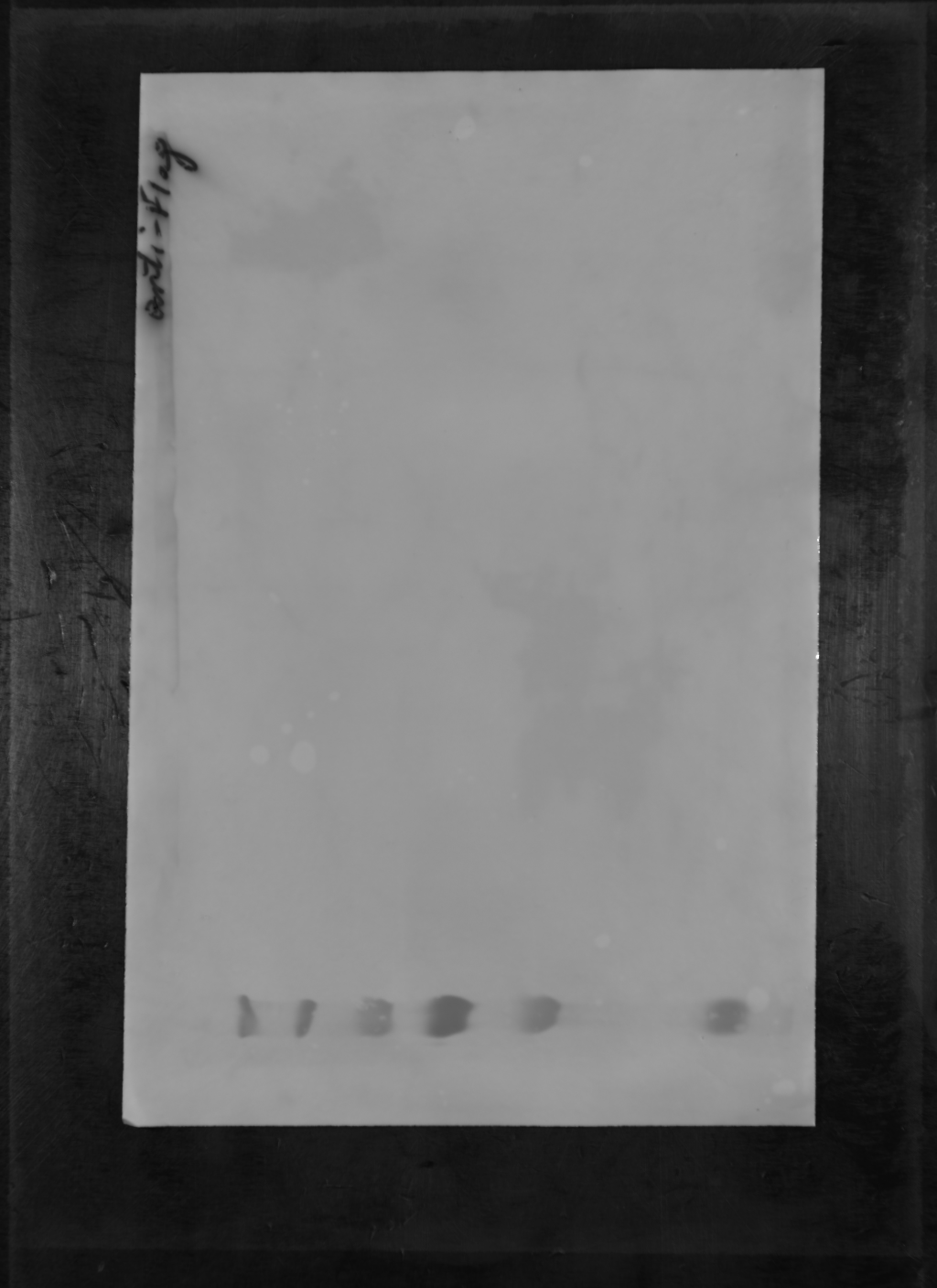

Supplement: Supplementary file 7 — Source data Fig. 5 [file 44318_2025_437_MOESM7_ESM.zip › Figure5/5F/Blot Flag_marker.tif]

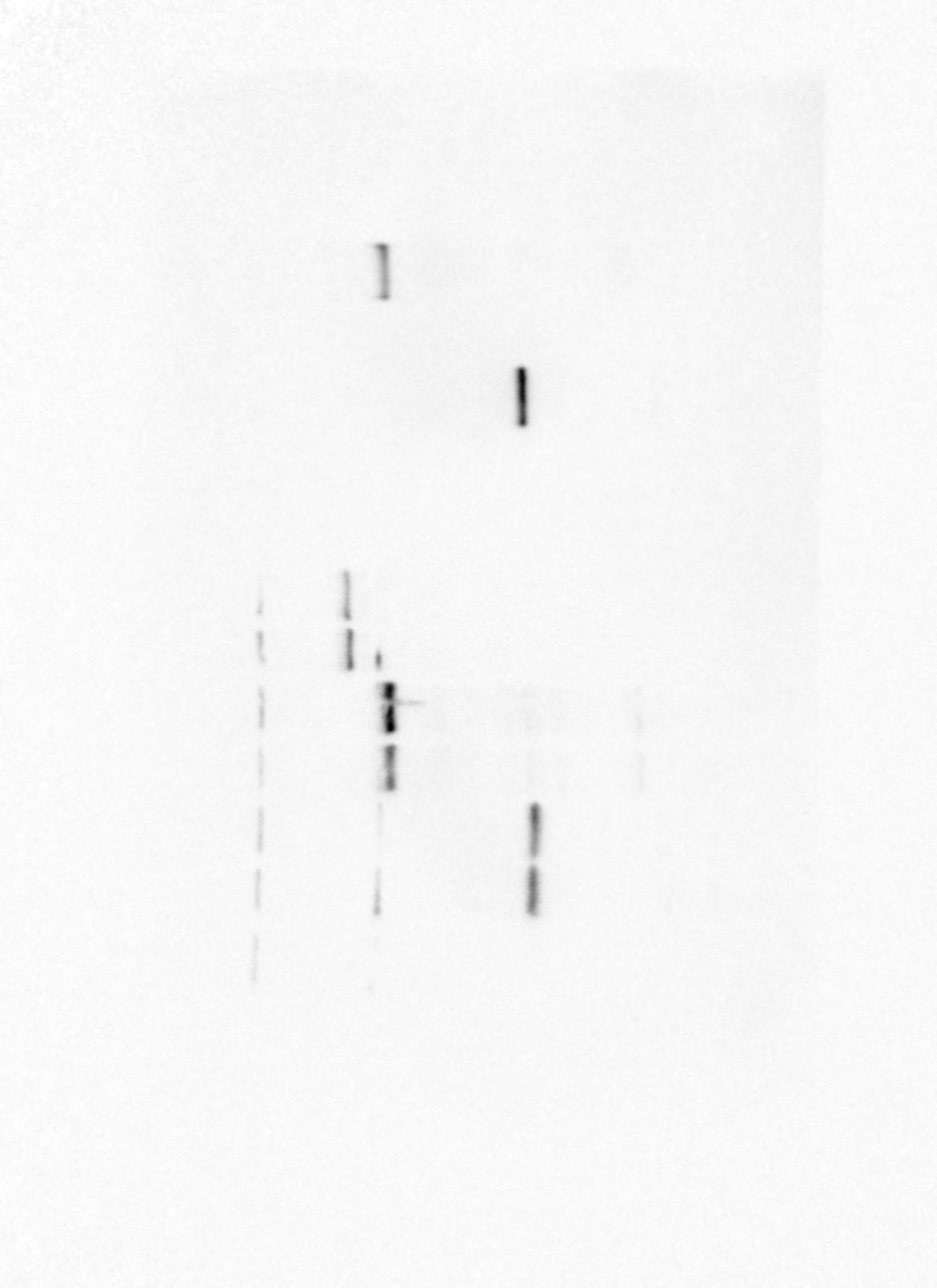

Supplement: Supplementary file 7 — Source data Fig. 5 [file 44318_2025_437_MOESM7_ESM.zip › Figure5/5F/Blot_Flag.tif]

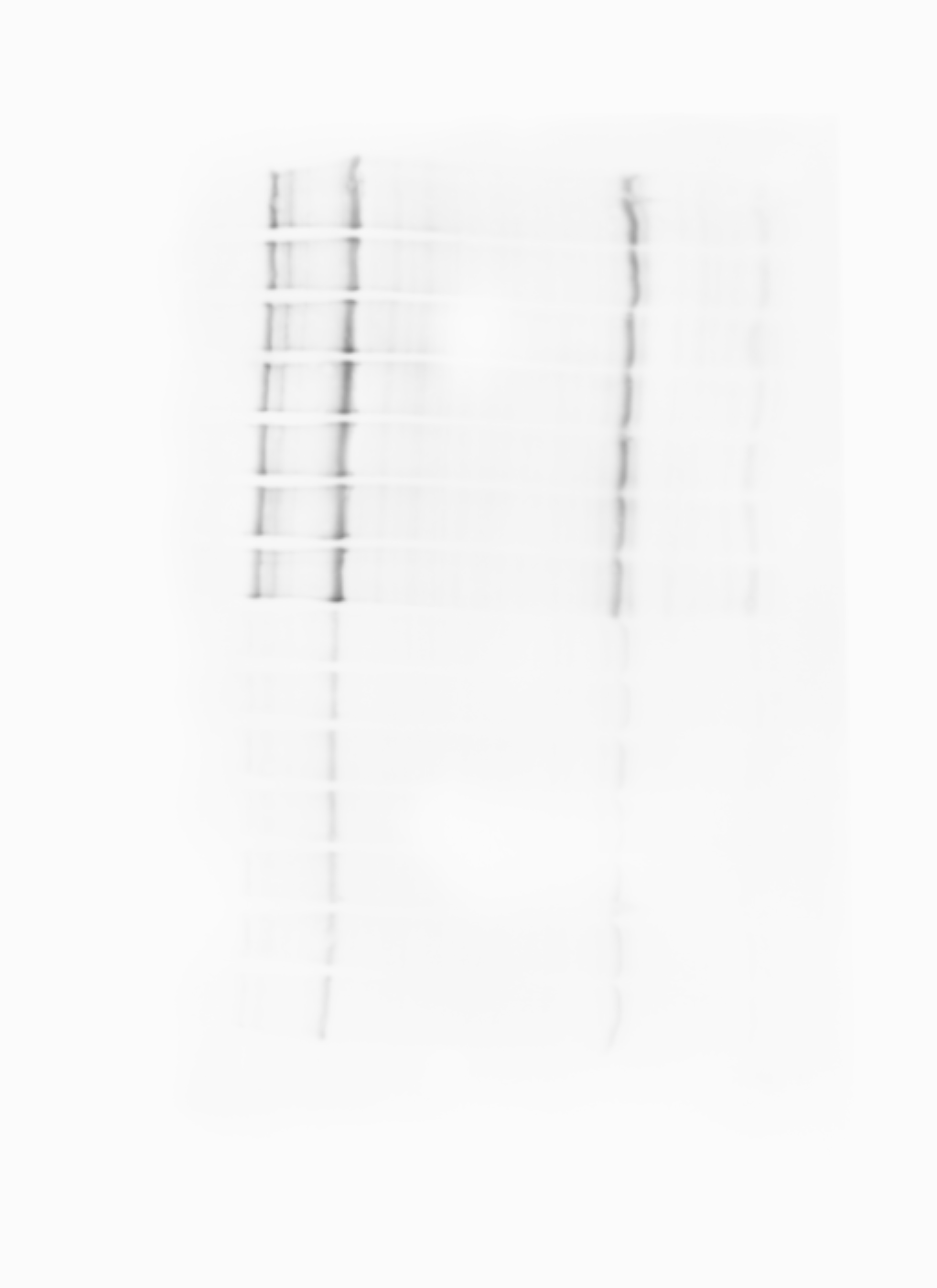

Supplement: Supplementary file 7 — Source data Fig. 5 [file 44318_2025_437_MOESM7_ESM.zip › Figure5/5F/Blot_Strep.tif]

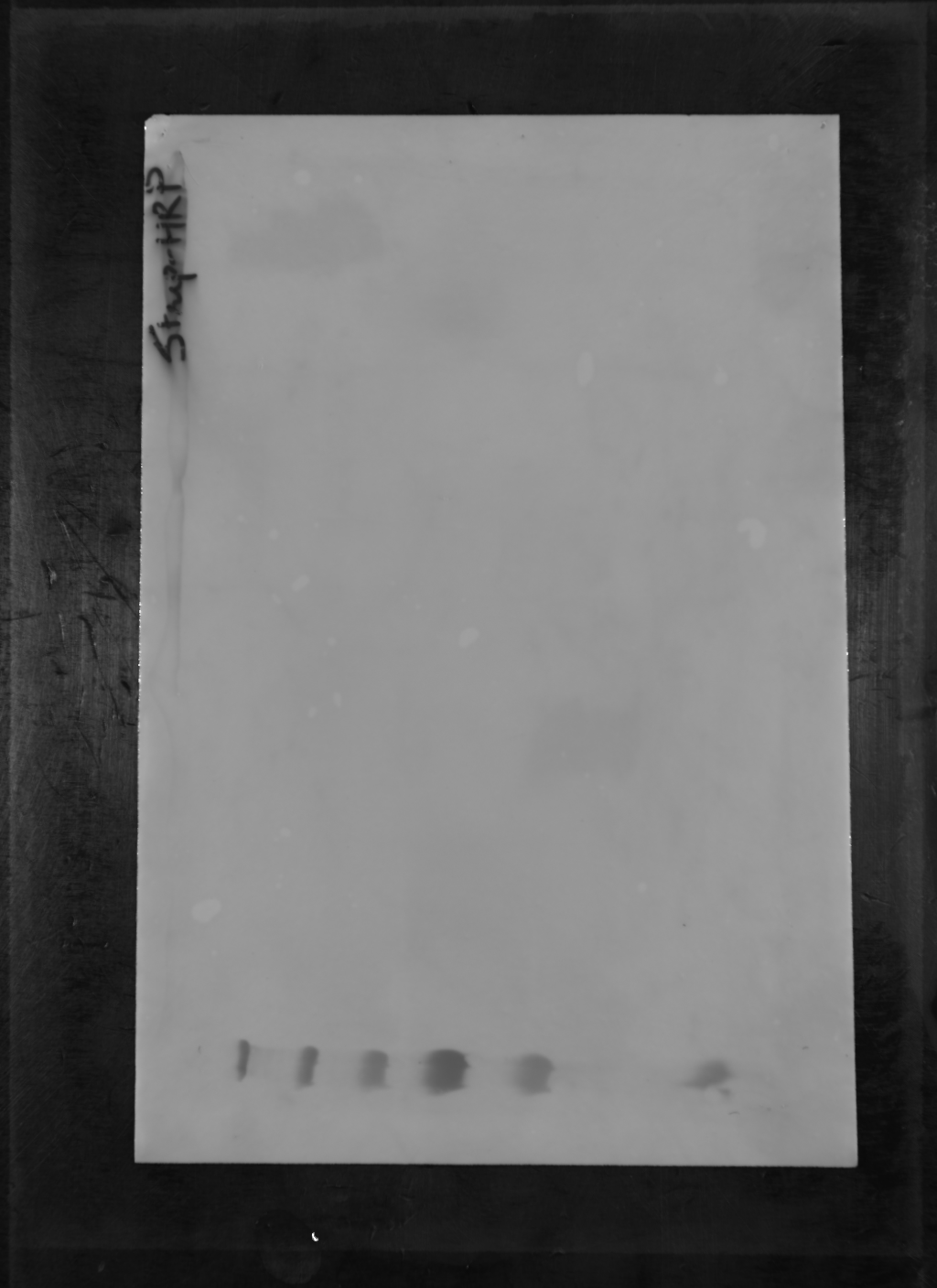

Supplement: Supplementary file 7 — Source data Fig. 5 [file 44318_2025_437_MOESM7_ESM.zip › Figure5/5F/Blot_Strep_marker.tif]

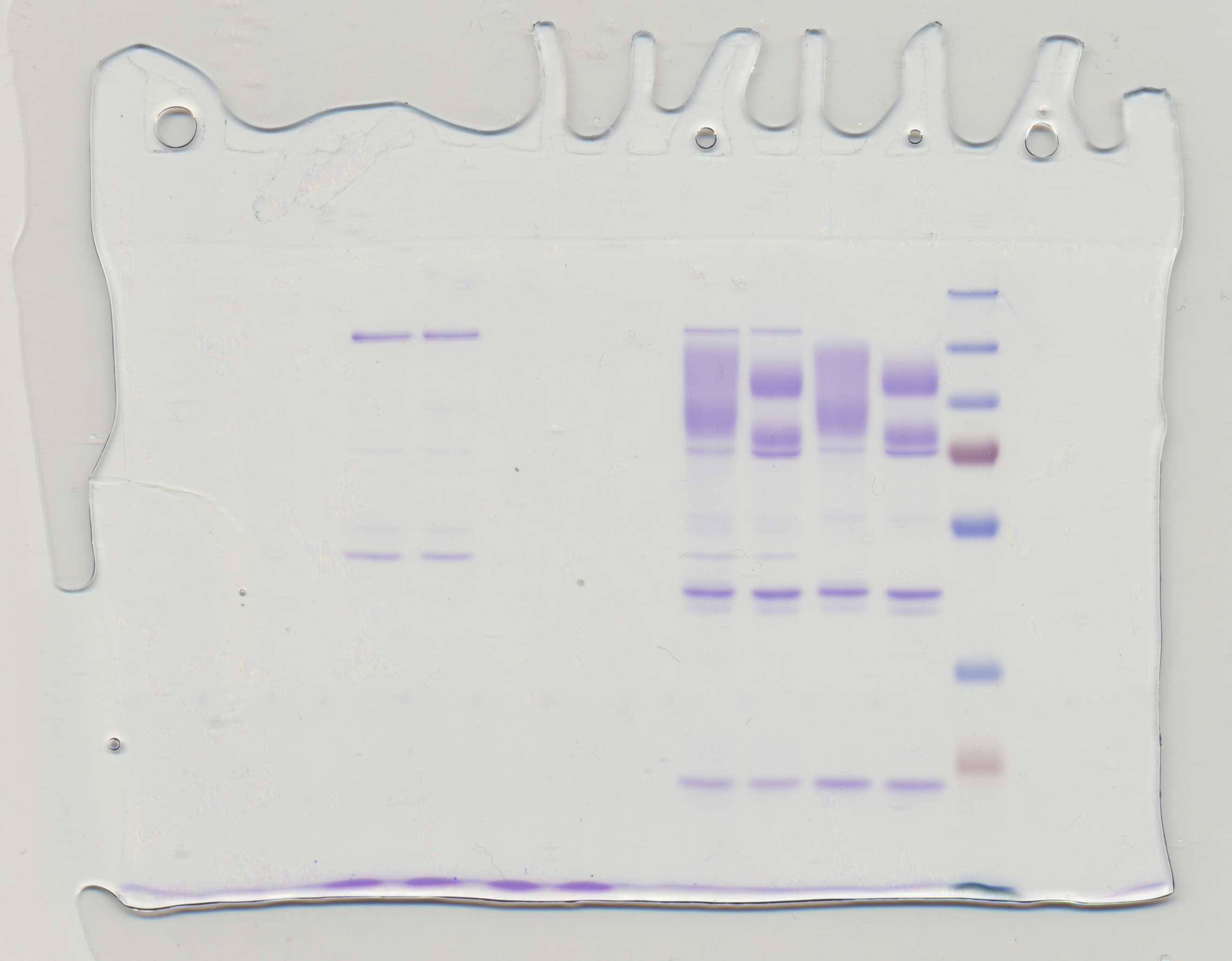

Supplement: Supplementary file 8 — Source data Fig. 7 [file 44318_2025_437_MOESM8_ESM.zip › Figure7/7D/PullDown_Coomassie.tif]

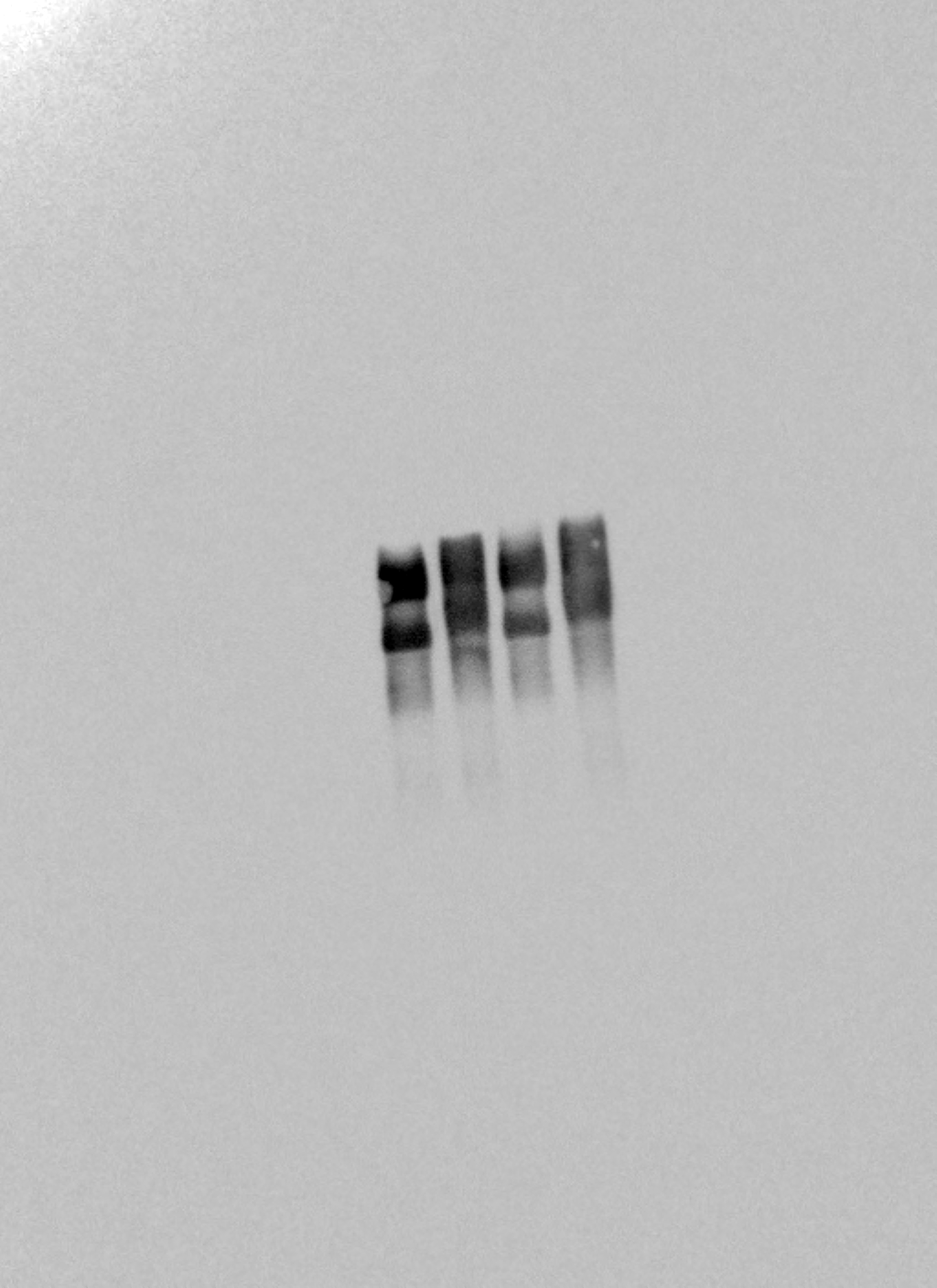

Supplement: Supplementary file 8 — Source data Fig. 7 [file 44318_2025_437_MOESM8_ESM.zip › Figure7/7D/PullDown_HisBlot_Input.tif]

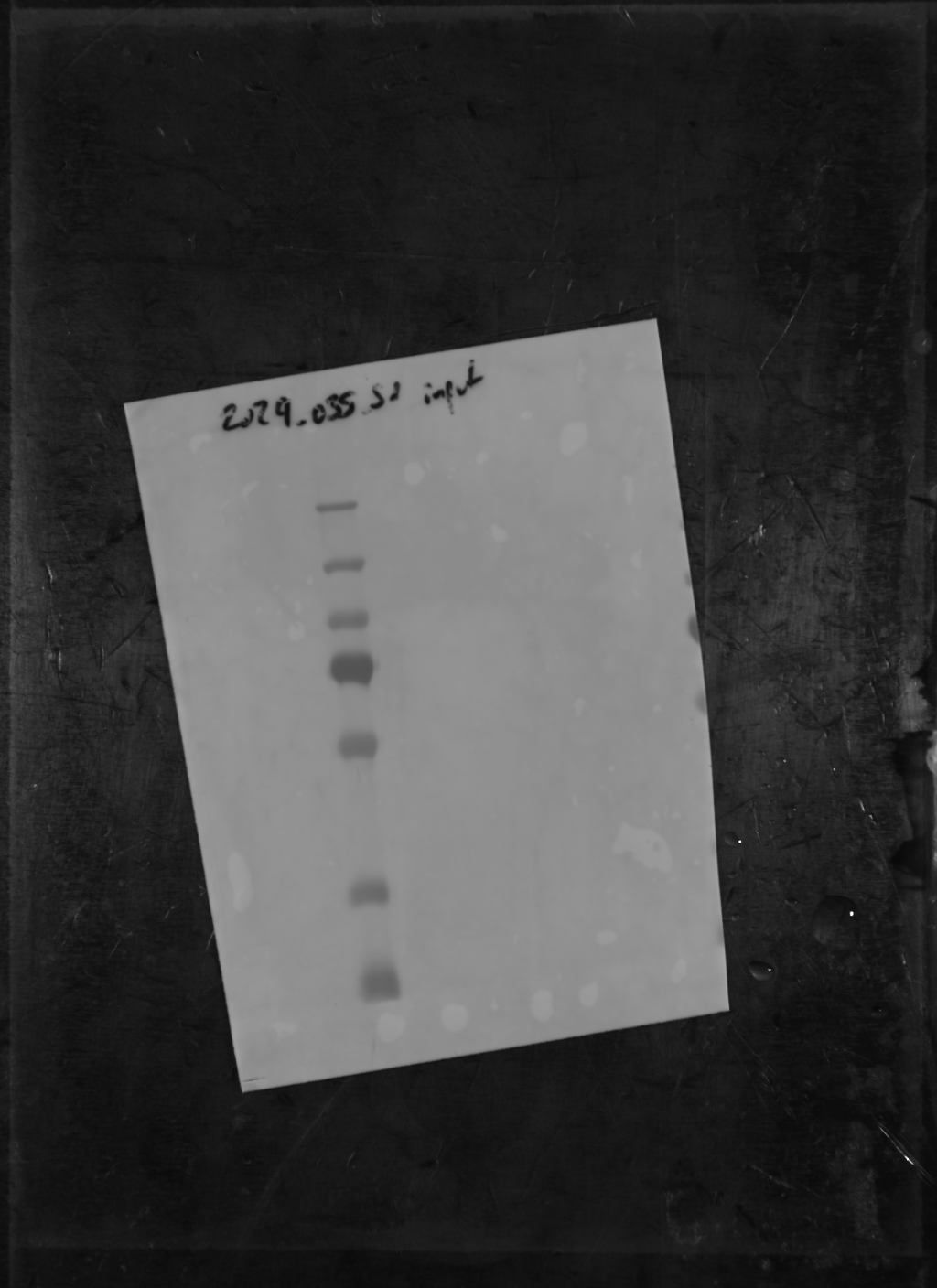

Supplement: Supplementary file 8 — Source data Fig. 7 [file 44318_2025_437_MOESM8_ESM.zip › Figure7/7D/PullDown_HisBlot_Input_marker.tif]

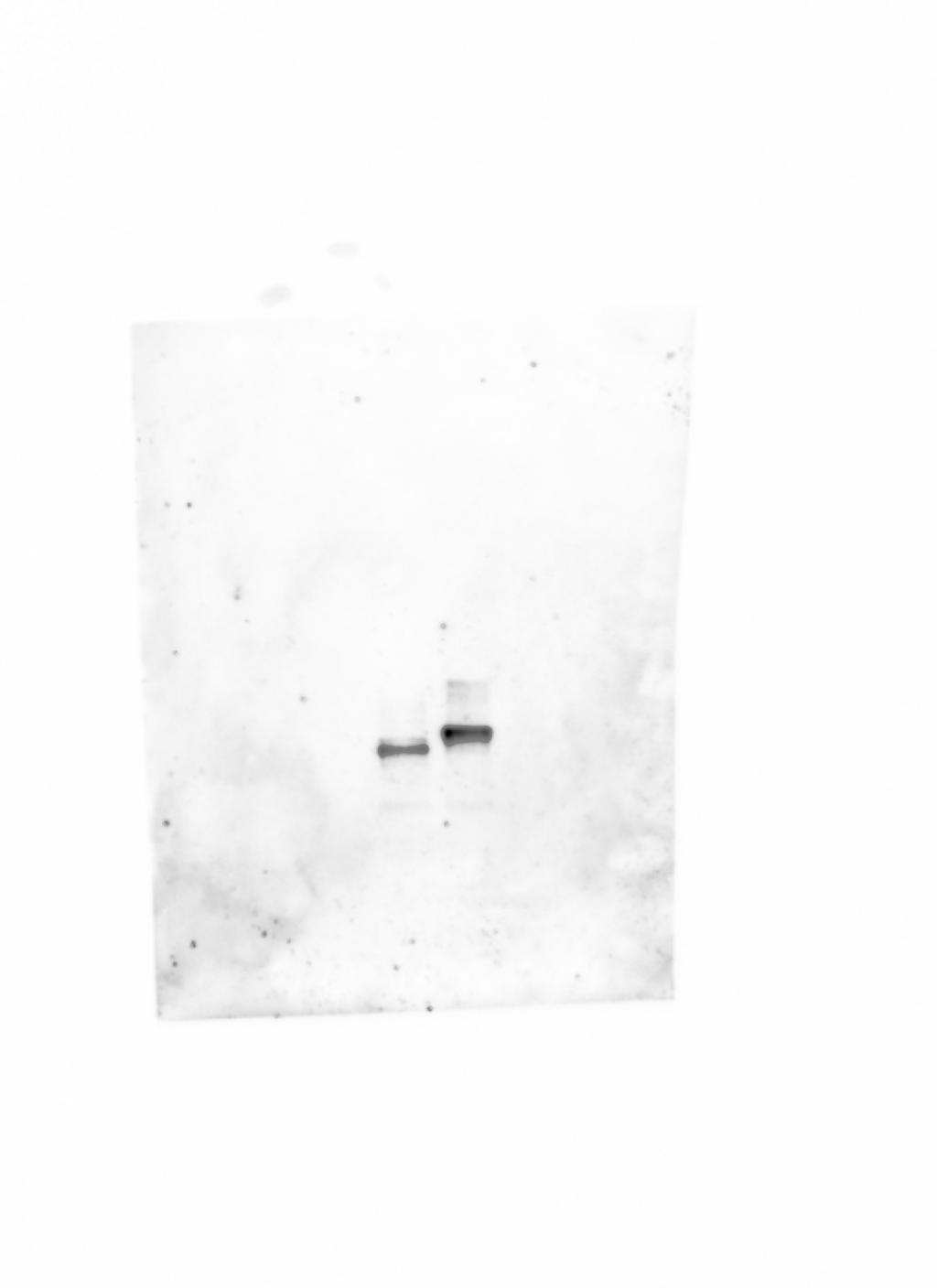

Supplement: Supplementary file 8 — Source data Fig. 7 [file 44318_2025_437_MOESM8_ESM.zip › Figure7/7D/PullDown_HisBlot_PullDown.tif]

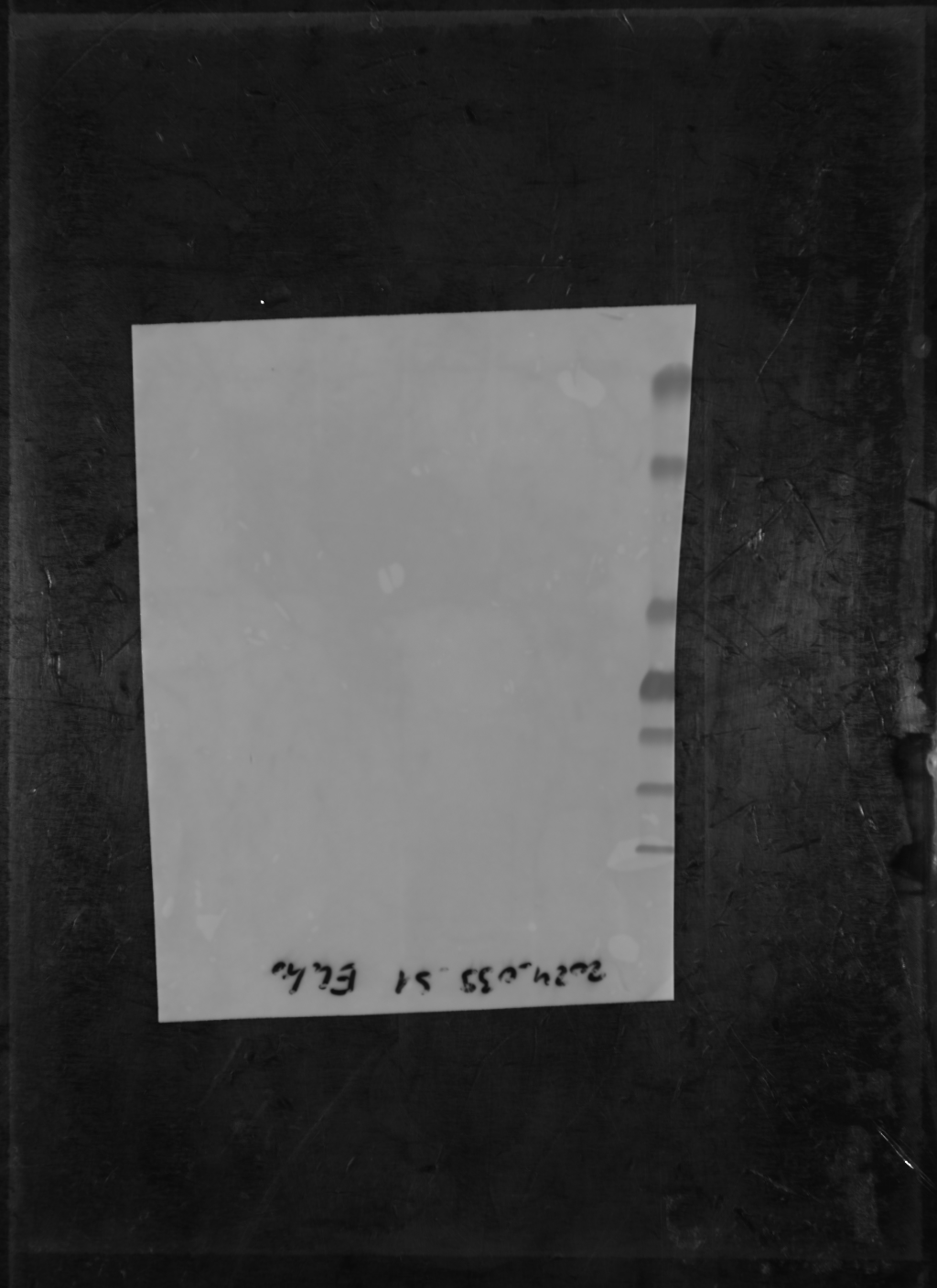

Supplement: Supplementary file 8 — Source data Fig. 7 [file 44318_2025_437_MOESM8_ESM.zip › Figure7/7D/PullDown_HisBlot_PullDown_marker.tif]

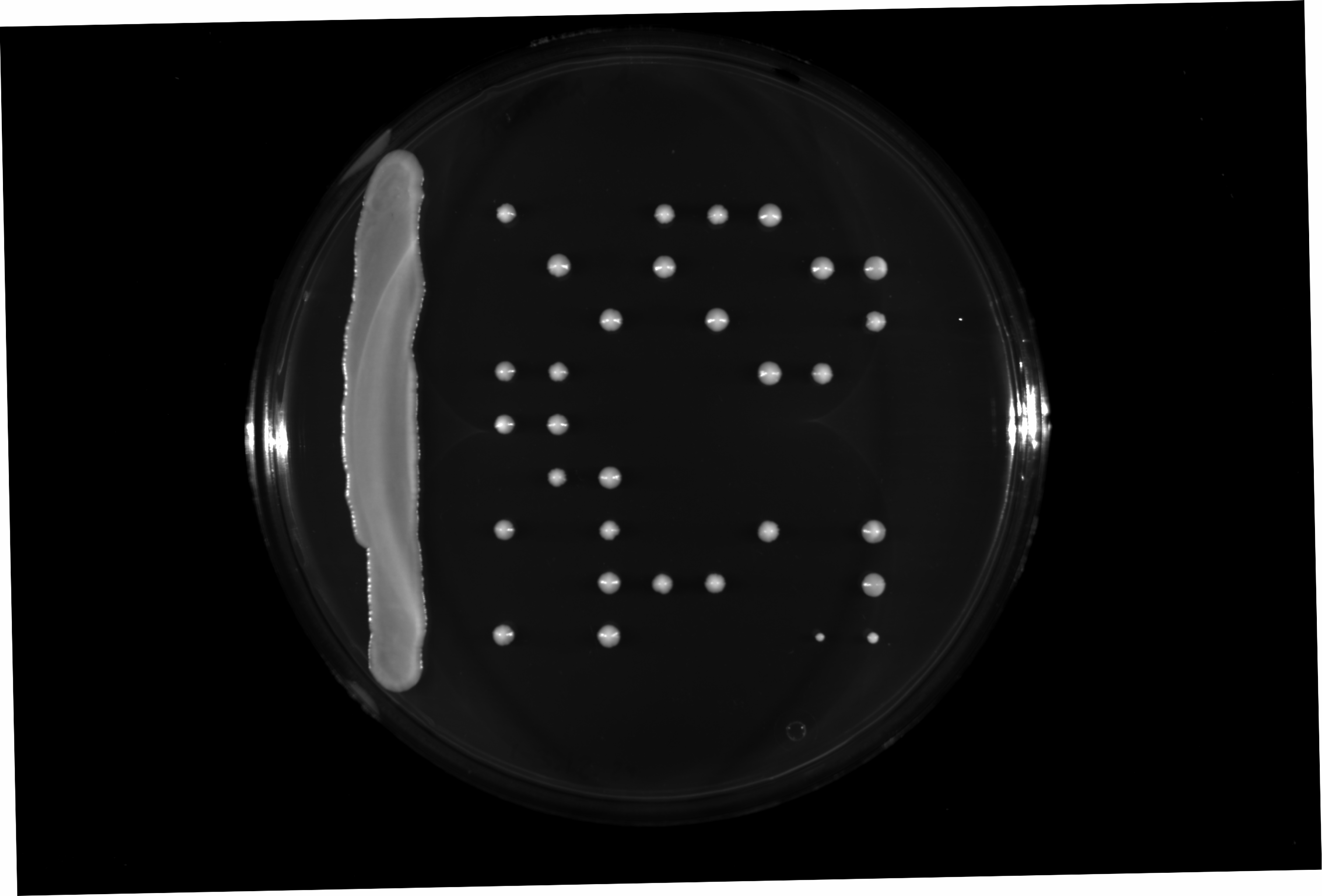

Supplement: Supplementary file 8 — Source data Fig. 7 [file 44318_2025_437_MOESM8_ESM.zip › Figure7/7E/DissectionPlate_Sli154E.tif]

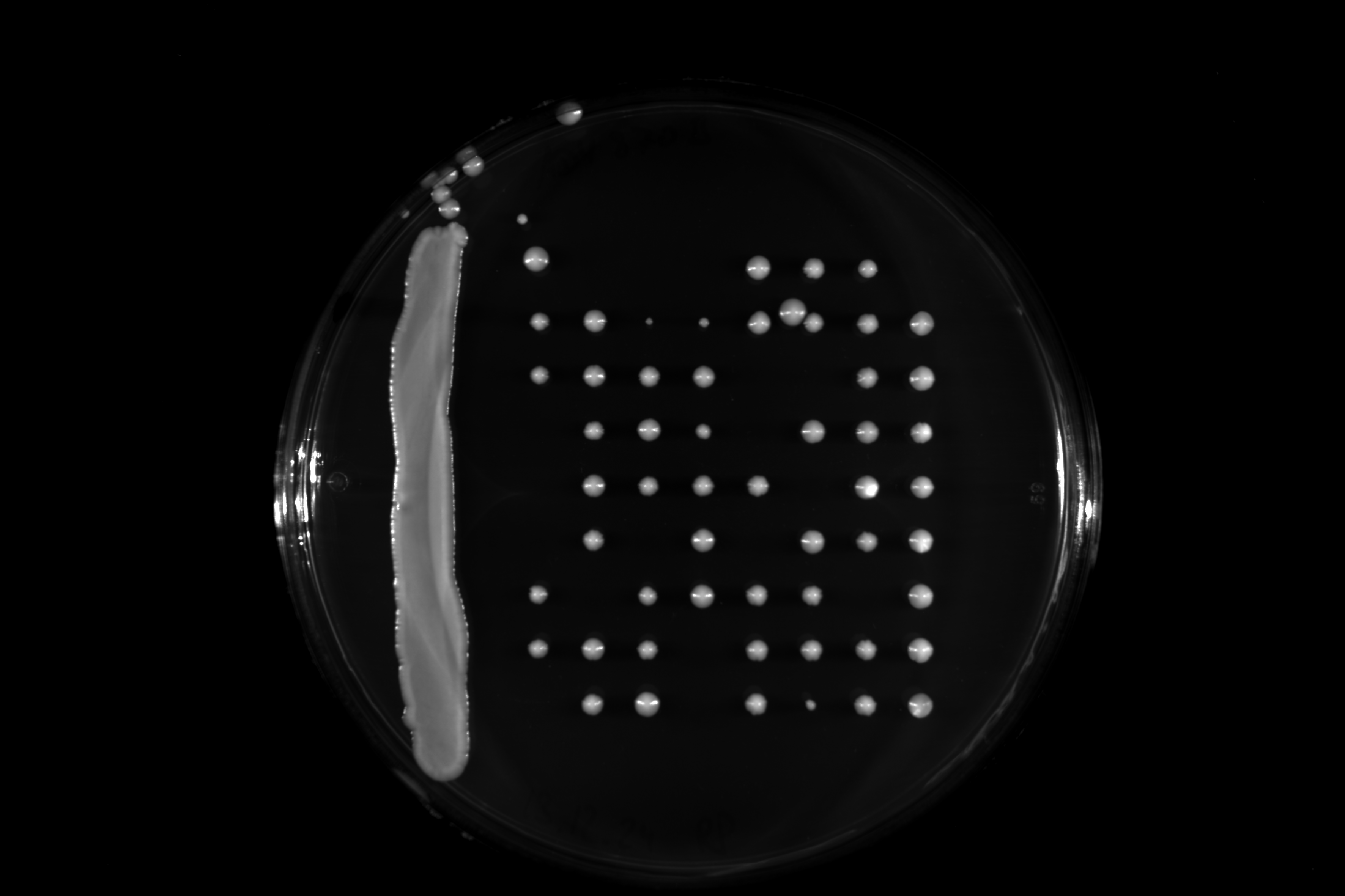

Supplement: Supplementary file 8 — Source data Fig. 7 [file 44318_2025_437_MOESM8_ESM.zip › Figure7/7E/DissectionPlate_Sli15deltaN.tif]

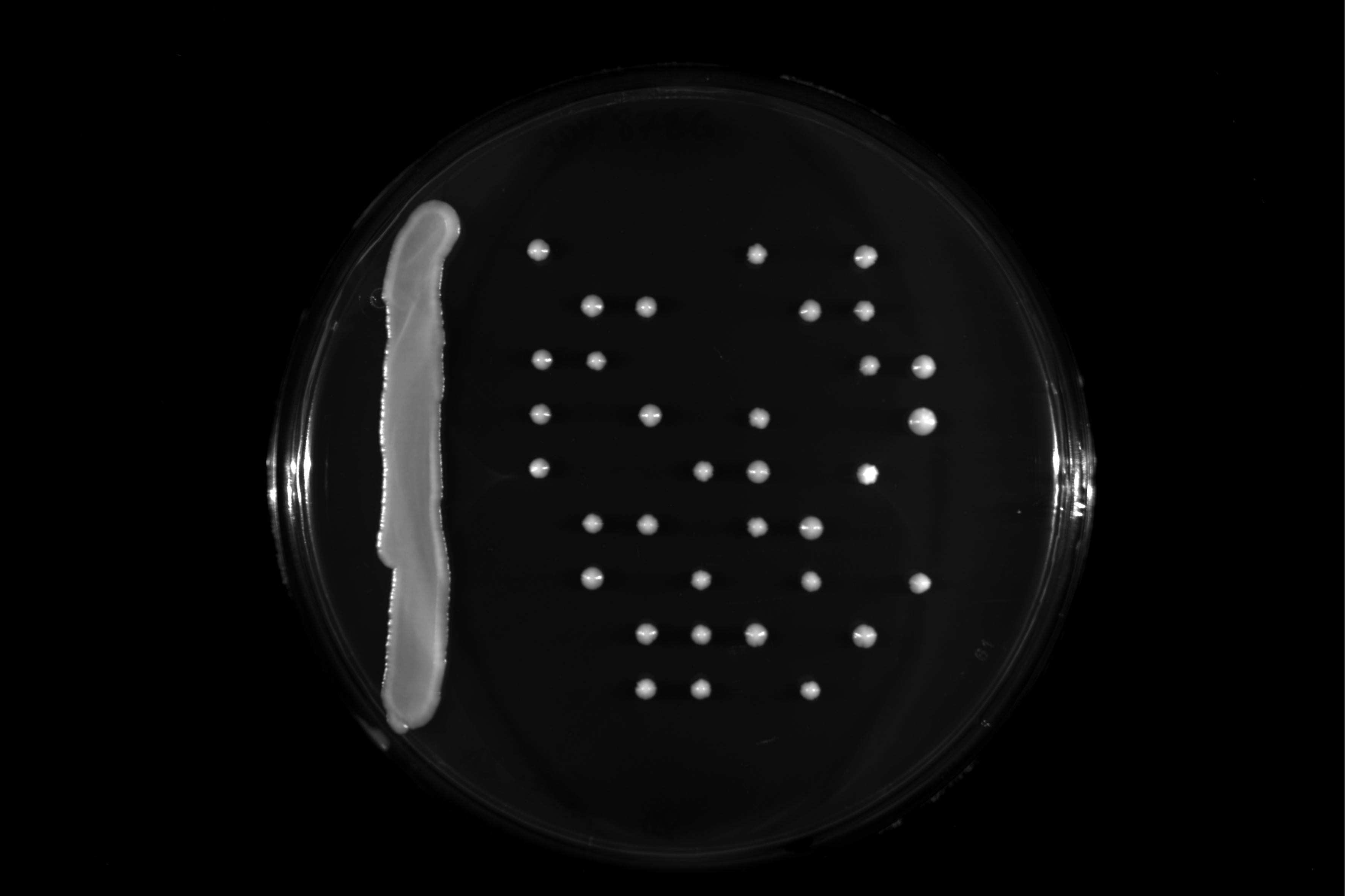

Supplement: Supplementary file 8 — Source data Fig. 7 [file 44318_2025_437_MOESM8_ESM.zip › Figure7/7E/DissectionPlate_Sli15deltaN4E.tif]

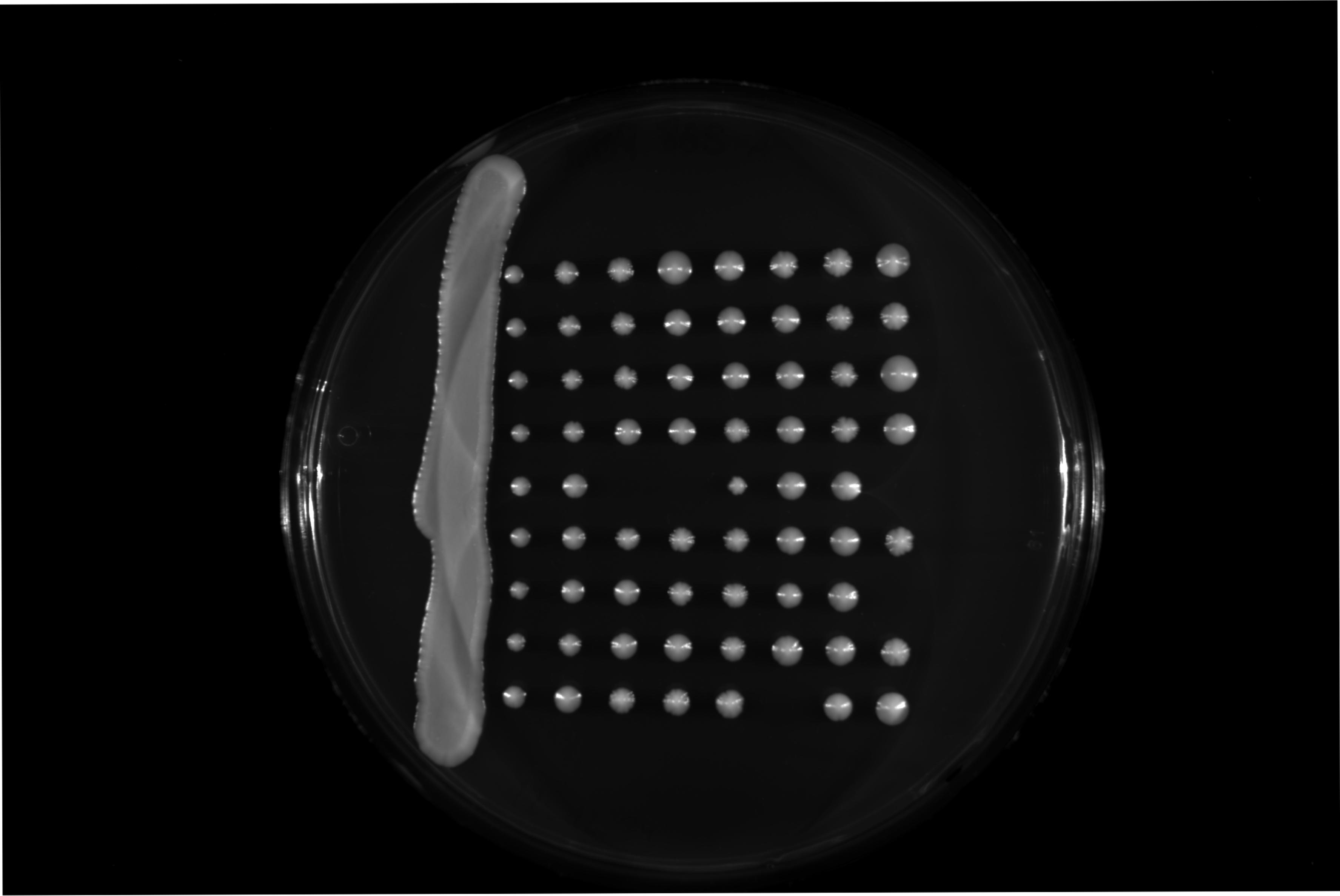

Supplement: Supplementary file 8 — Source data Fig. 7 [file 44318_2025_437_MOESM8_ESM.zip › Figure7/7E/DissectionPlate_Sli15wt.tif]

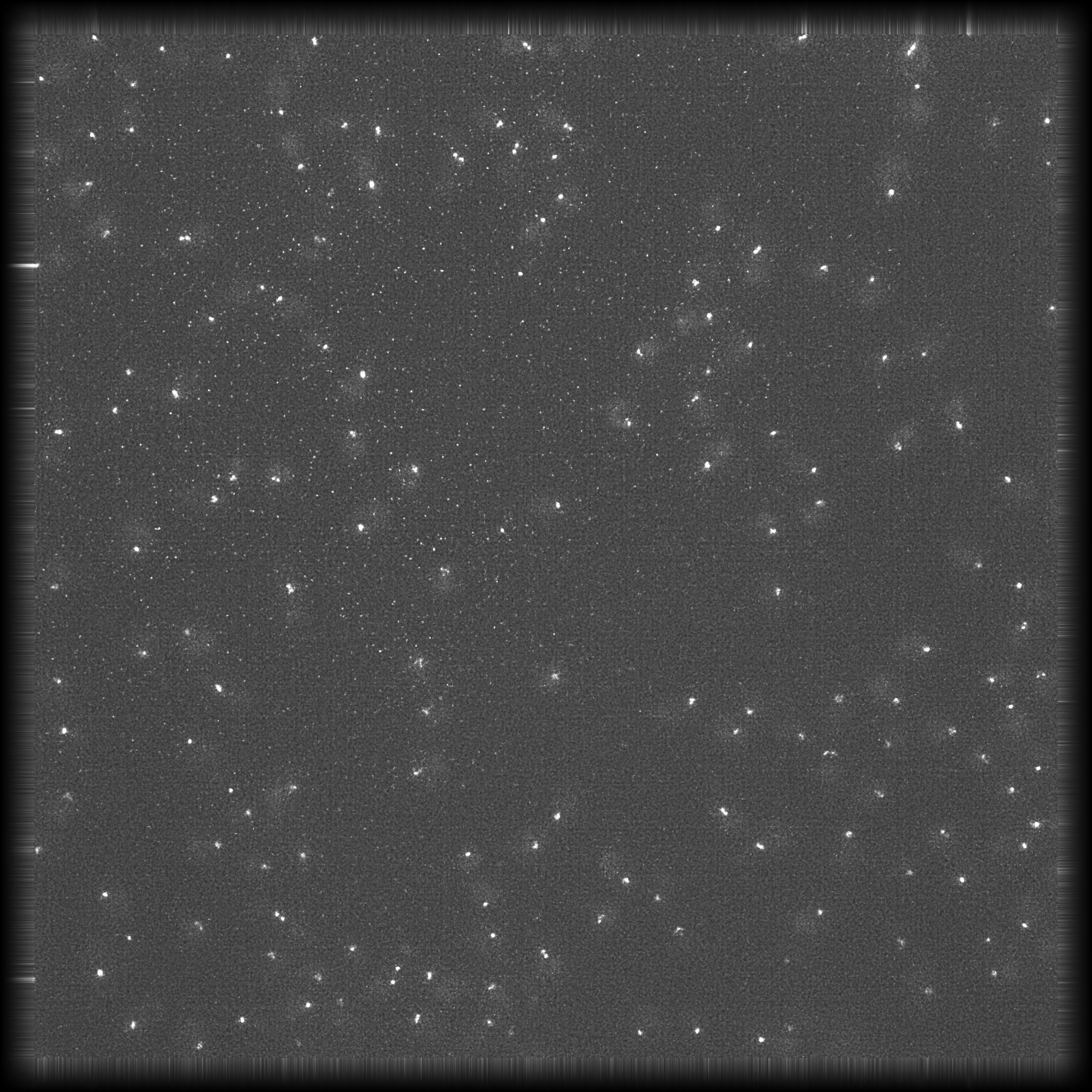

Supplement: Supplementary file 8 — Source data Fig. 7 [file 44318_2025_437_MOESM8_ESM.zip › Figure7/7F/WholeMount_Sli15_4E.tif]

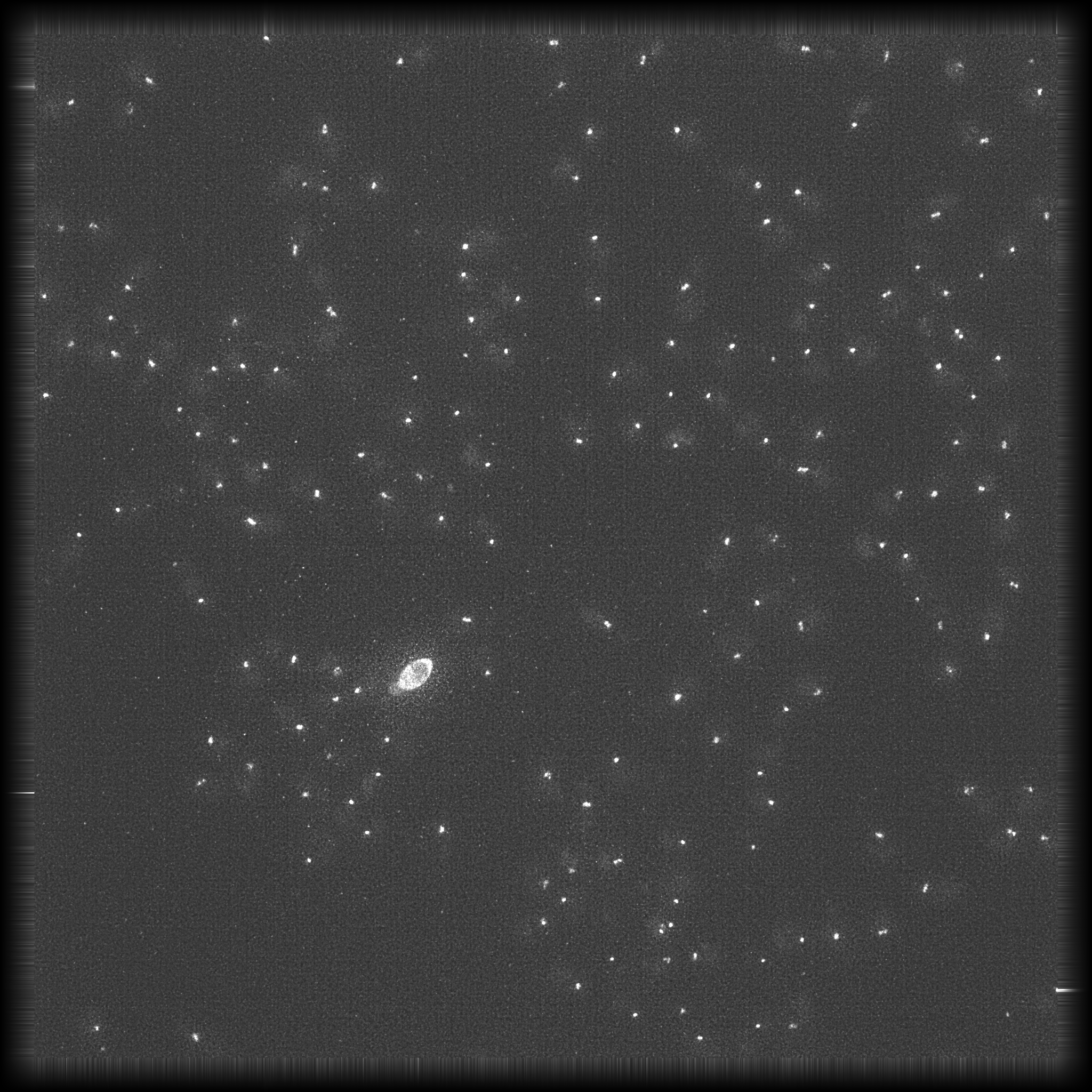

Supplement: Supplementary file 8 — Source data Fig. 7 [file 44318_2025_437_MOESM8_ESM.zip › Figure7/7F/WholeMount_Sli15_deltaN.tif]

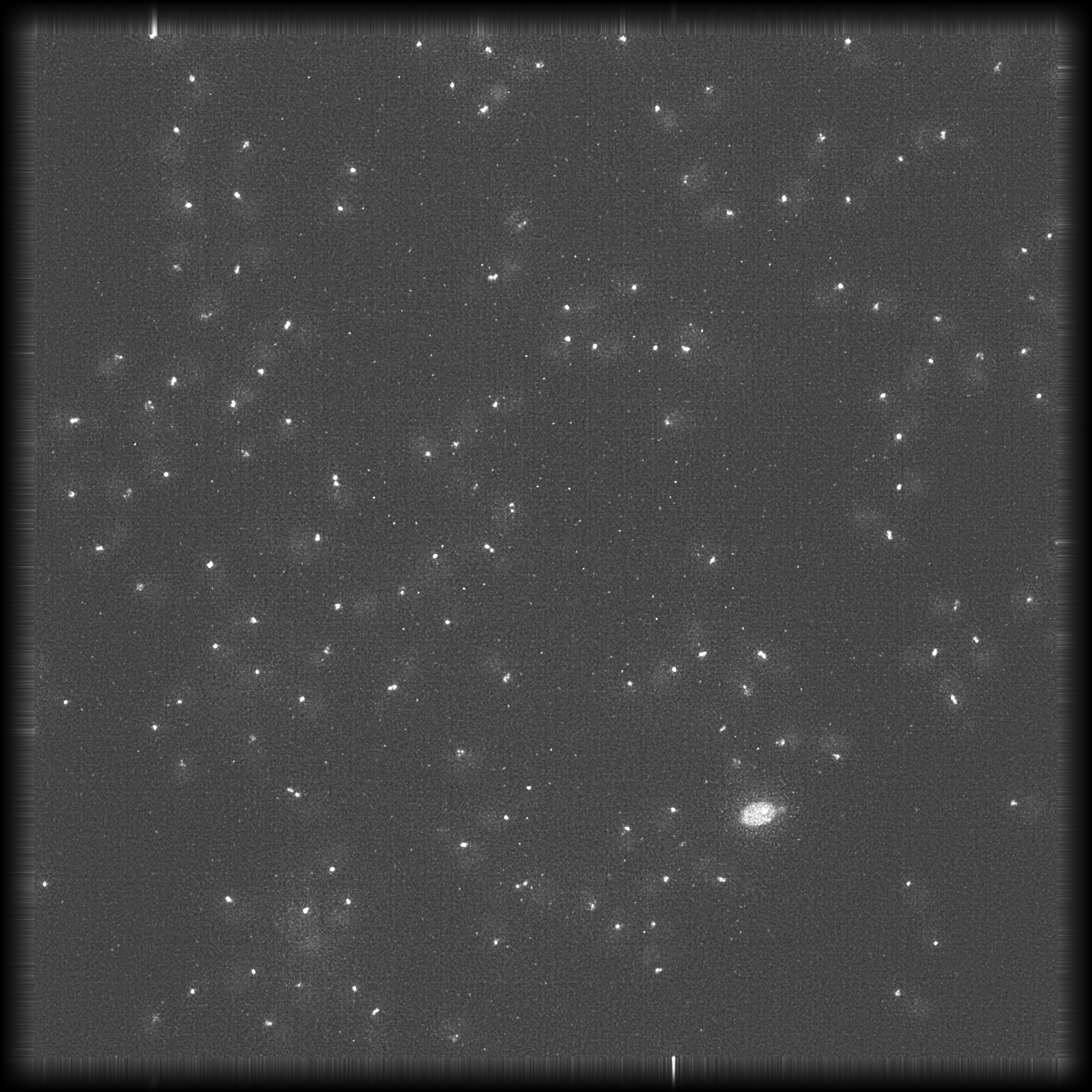

Supplement: Supplementary file 8 — Source data Fig. 7 [file 44318_2025_437_MOESM8_ESM.zip › Figure7/7F/WholeMount_Sli15_deltaN4E.tif]

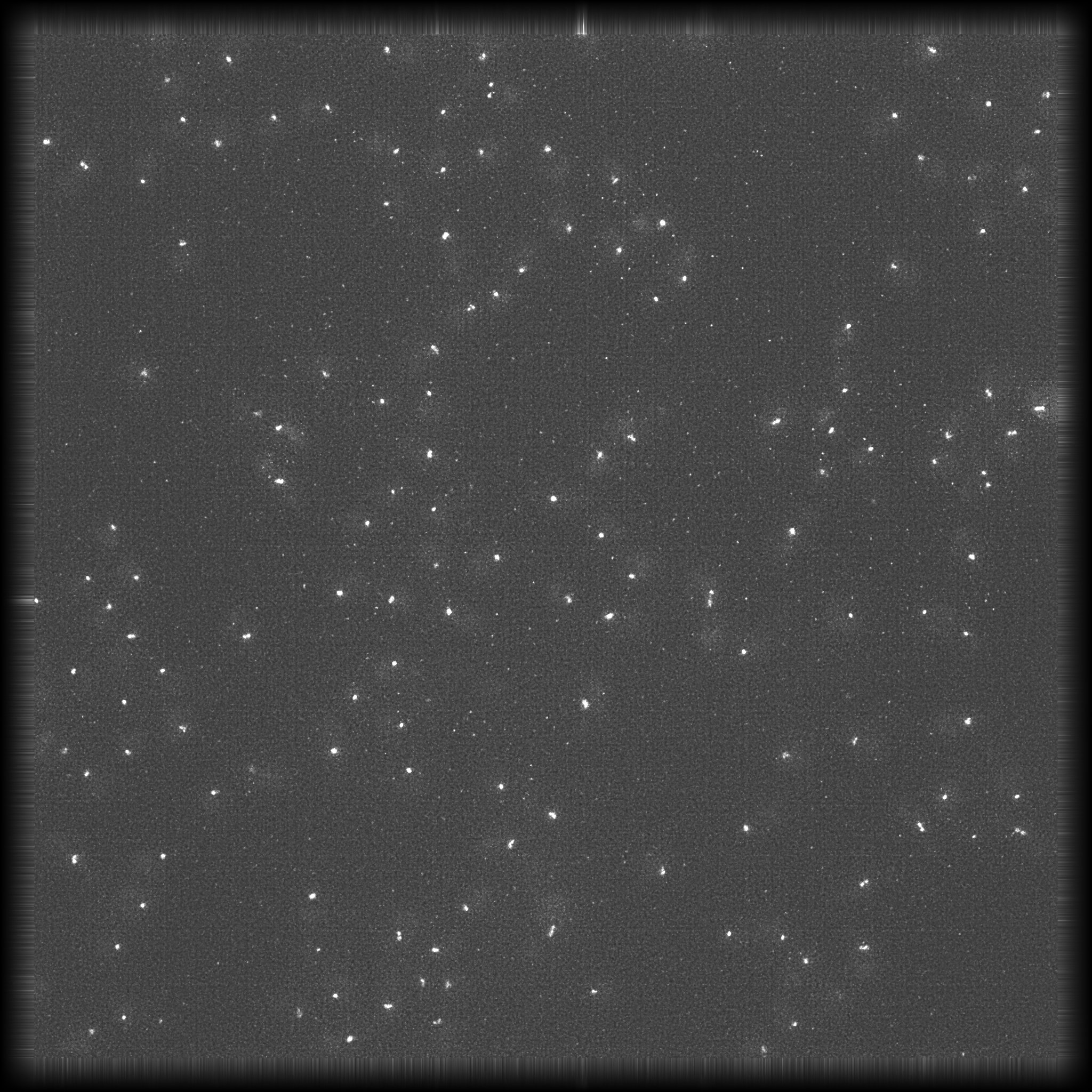

Supplement: Supplementary file 8 — Source data Fig. 7 [file 44318_2025_437_MOESM8_ESM.zip › Figure7/7F/WholeMount_Sli15_wt.tif]
