# Supplementary material for: The Spc105/Kre28 complex promotes mitotic error correction by outer kinetochore recruitment of Ipl1/Sli15
Source: EMBO J. 2025 Apr 25;44(12):3492–520. doi: 10.1038/s44318-025-00437-w (PMC12170873; doi:10.1038/s44318-025-00437-w)
Supplement: Supplementary file 6 — Source data Fig. 4 [file 44318_2025_437_MOESM6_ESM.zip › Figure4/4C/information on SDA 20 09 23.docx]

**Upper plate**

1. ADY565 Kre28^wt^
2. ADY567 Kre28^∆Zwint^
3. ADY768 Kre28^wt^, Fusion I
4. ADY769 6A Kre28^∆Zwint^, Fusion I
5. ADY769 7A Kre28^∆Zwint^, Fusion I
6. ADY802.1 Kre28^wt^, Fusion II
7. ADY803.2 Kre28^∆Zwint^, Fusion II
8. ADY803.3 Kre28^∆Zwint^, Fusion II

**lower plate**

1. ADY565 Kre28^wt^
2. ADY567 Kre28^∆Zwint^
3. ADY769 6A Kre28^∆Zwint^, Fusion I
4. ADY803.2 Kre28^∆Zwint^, Fusion II
5. ADY794.1 Kre28^wt^, Fusion I mut.
6. ADY795.1 Kre28^∆Zwint^, Fusion I mut.
7. ADY840.1 Kre28^wt^, Fusion II mut.
8. ADY841 Kre28^∆Zwint^, Fusion II mut.
