# Supplementary material for: The Spc105/Kre28 complex promotes mitotic error correction by outer kinetochore recruitment of Ipl1/Sli15
Source: EMBO J. 2025 Apr 25;44(12):3492–520. doi: 10.1038/s44318-025-00437-w (PMC12170873; doi:10.1038/s44318-025-00437-w)
Supplement: Supplementary file 9 — Expanded View Figures [file 44318_2025_437_MOESM9_ESM.pdf]

Expanded View Figures

Figure EV1. Refers to Fig. 1: Detailed mutagenesis analysis of the Zwint helix.

(**A**) Multiple sequence alignment of Kre28 sequences from different yeast species. Conserved residues are colored according to the Clustal color scheme. Positions of smaller Zwint helix deletions and alanine substitutions of conserved residues are marked. (**B**) Western blot analysis of different Kre28/Zwint deletion or point mutants. Extracts of log-phase yeast cells were analyzed, Pgk1 blot serves as a loading control. (**C**, **D**) Serial dilution assays of haploid yeast strains with different Kre28 Zwint helix deletions or point mutations of conserved residues. Strains with the indicated genotypes were serially diluted and spotted on YEPD or YEPD + benomyl plates and incubated at the indicated temperatures.

▶

© The Author(s)

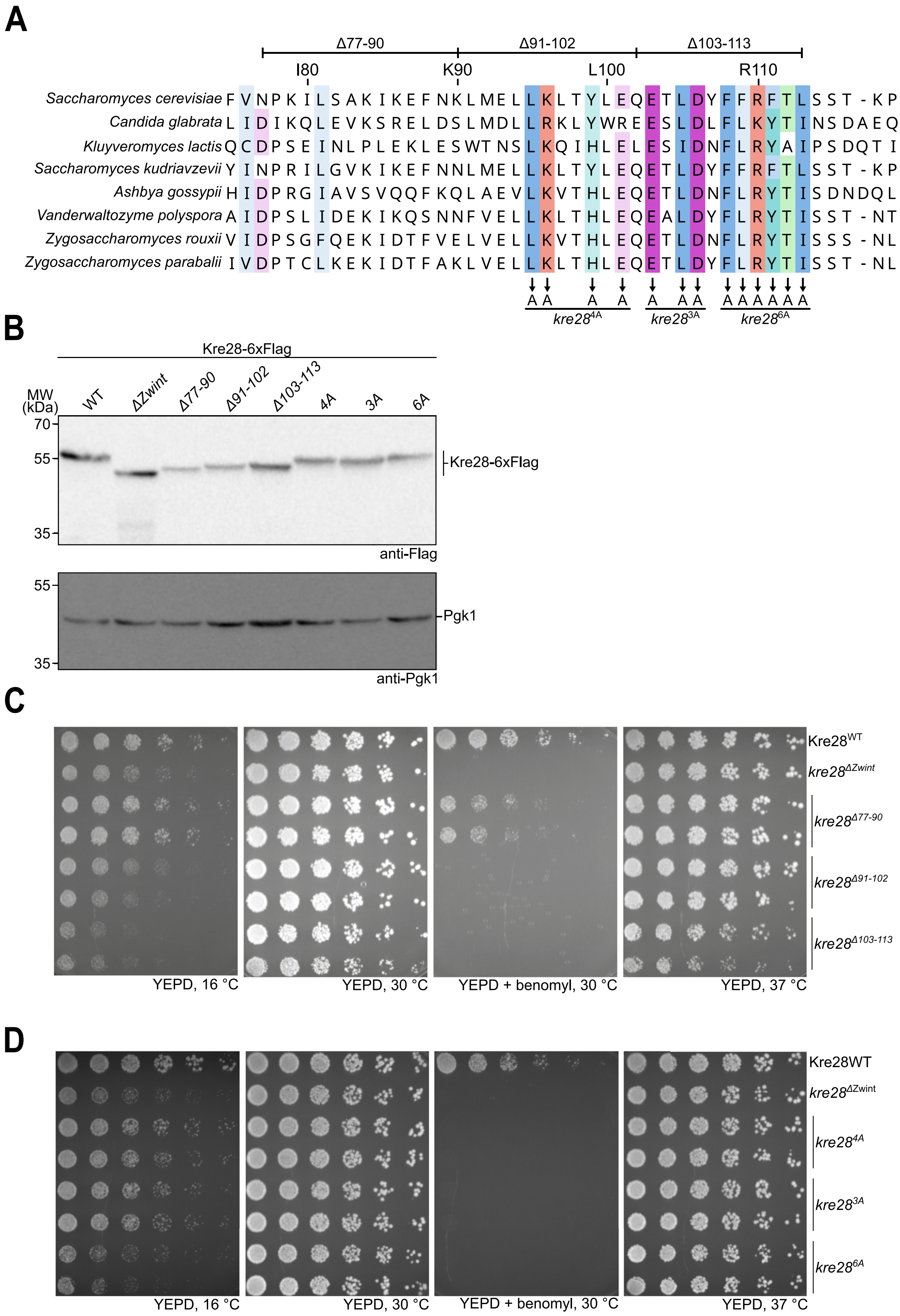
A
Δ77-90
Δ91-102
Δ103-113
I80
K90
L100
R110
Q120
Saccharomyces cerevisiae
Candida glabrata
Kluyveromyces lactis
Saccharomyces kudriavzevii
Ashbya gossypii
Vanderwaltozyme polyspora
Zygosaccharomyces rouxii
Zygosaccharomyces parabalii
kre28^4A
kre28^3A
kre28^6A
B
Kre28-6xFlag
MW (kDa)
WT
ΔZwint
Δ77-90
Δ91-102
Δ103-113
4A
3A
6A
70
55
35
Kre28-6xFlag
anti-Flag
Pgk1
anti-Pgk1
C
Kre28^WT
kre28^ΔZwint
kre28^Δ77-90
kre28^Δ91-102
kre28^Δ103-113
YEPD, 16 °C
YEPD, 30 °C
YEPD + benomyl, 30 °C
YEPD, 37 °C
D
Kre28WT
kre28^ΔZwint
kre28^4A
kre28^3A
kre28^6A
YEPD, 16 °C
YEPD, 30 °C
YEPD + benomyl, 30 °C
YEPD, 37 °C

 © The Author(s)

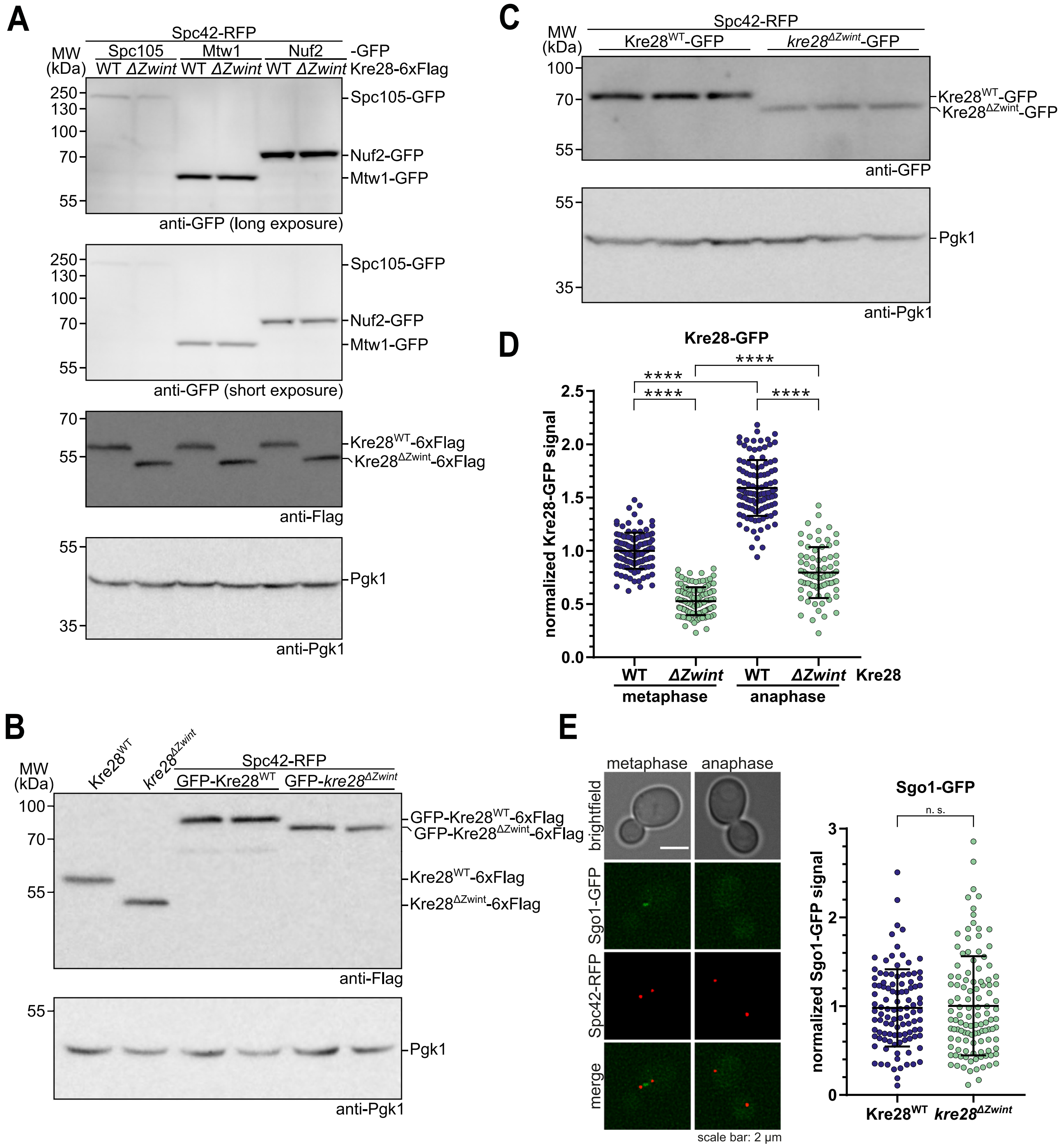
A
Spc42-RFP
MW (kDa)
Spc105 Mtw1 Nuf2 -GFP
WT ΔZwint WT ΔZwint WT ΔZwint Kre28-6xFlag
250
130
100
70
55
Spc105-GFP
Nuf2-GFP
Mtw1-GFP
anti-GFP (long exposure)
anti-GFP (short exposure)
Kre28WT-6xFlag
Kre28ΔZwint-6xFlag
anti-Flag
Pgk1
35
anti-Pgk1
B
Kre28WT
kre28ΔZwint
Spc42-RFP
GFP-Kre28WT
GFP-kre28ΔZwint
GFP-Kre28WT-6xFlag
GFP-Kre28ΔZwint-6xFlag
anti-Flag
anti-Pgk1
C
Spc42-RFP
Kre28WT-GFP
kre28ΔZwint-GFP
Kre28ΔZwint-GFP
anti-GFP
anti-Pgk1
D
Kre28-GFP
****
normalized Kre28-GFP signal
0.0
0.5
1.0
1.5
2.0
2.5
WT ΔZwint WT ΔZwint Kre28
metaphase
anaphase
E
metaphase
anaphase
brightfield
Sgo1-GFP
Spc42-RFP
merge
scale bar: 2 µm
Sgo1-GFP
n. s.
normalized Sgo1-GFP signal
0
1
2
3
Kre28WT
kre28ΔZwint

© The Author(s)

Figure EV2. Refers to Fig. 3: Western blot analysis of GFP-tagged KMN subunits and live-cell microscopy of Sgo1-GFP.

(**A**) Western blot analysis to compare the expression of different GFP-tagged KMN subunits. in Kre28 wild-type and mutant cells. Pgk1 served as loading control. (**B**) Western blot analysis of expression level of N-terminal GFP-tagged Kre28 in wild-type and mutant form. (**C**) Western blot analysis of C-terminal GFP-tagged Kre28 in wild-type and mutant form. (**D**) Quantification of Kre28-GFP fluorescence signals at metaphase and anaphase kinetochores. Strains with either $Kre28^{WT}$-GFP or *kre28*$^{\Delta Zwint}$-GFP were analyzed by live-cell microscopy. The C-terminus of Spc42 was fused to RFP to label the position of the mitotic spindle. The mean of metaphase signal intensities in the wild-type strain was defined as 1 and all other data was normalized to this value. Means +/− standard deviation are plotted. *P* values were calculated with a Kruskal–Wallis test, and displayed as follows: ****$P < 0.0001$. Exact *P* values for (**E**): in all cases ****<0.0001. $n > 100$ kinetochore clusters were analyzed for each condition. Representative data from two independent experiments is shown. (**E**) Live-cell microscopy for analysis of Sgo1-GFP kinetochore localization in a $Kre28^{WT}$ or *kre28*$^{\Delta Zwint}$ strain background. Representative images of a metaphase and an anaphase cell are shown on the left and quantification of Sgo1-GFP signals in cells with short mitotic spindles is shown on the right. Data was normalized to the mean value measured for the $Kre28^{WT}$ strain. Mean values +/− standard deviation are plotted. A Mann–Whitney test was used to test for statistical significance.

© The Author(s)

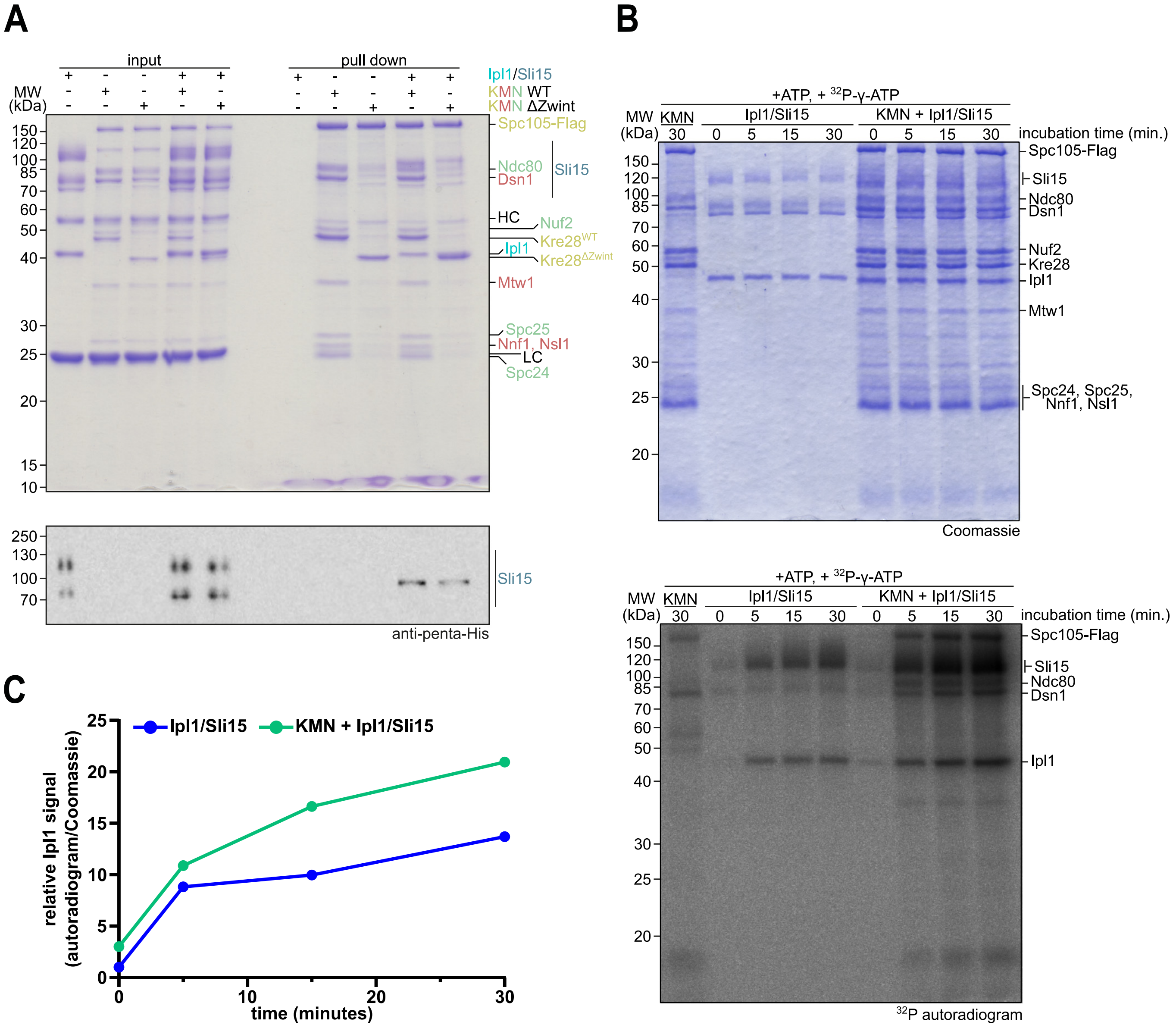

Figure EV3. Refers to Fig. 5: Pull -down experiment and in vitro kinase reaction with Ipl1/Sli15 and KMN.

(**A**) Pull-down assays to analyze binding of Ipl1/Sli15 to KMN. KMN with either Kre28^{WT} or Kre28$^{\Delta Zwint}$ was purified from Sf9 cells and immobilized on M2 anti-Flag beads. Loaded beads were incubated with buffer or recombinant Ipl1/Sli15, afterwards proteins bound to the beads were eluted with 3xFlag peptide. Input and pull-down samples were analyzed by SDS-PAGE and Coomassie staining or western blot to detect 6xHis-tagged Sli15. (**B**) In vitro kinase reaction with Ipl1/Sli15 or Ipl1/Sli15 in the presence of recombinant KMN analyzed over time. Coomassie-stained gel at the top, corresponding autoradiograph at the bottom. For control a KMN only sample taken 30 min after incubation with radioactive ATP is shown in the first lane. (**C**) Quantification phosphorylated Ipl1 signal over time alone (blue curve) or in the presence of KMN (green curve).

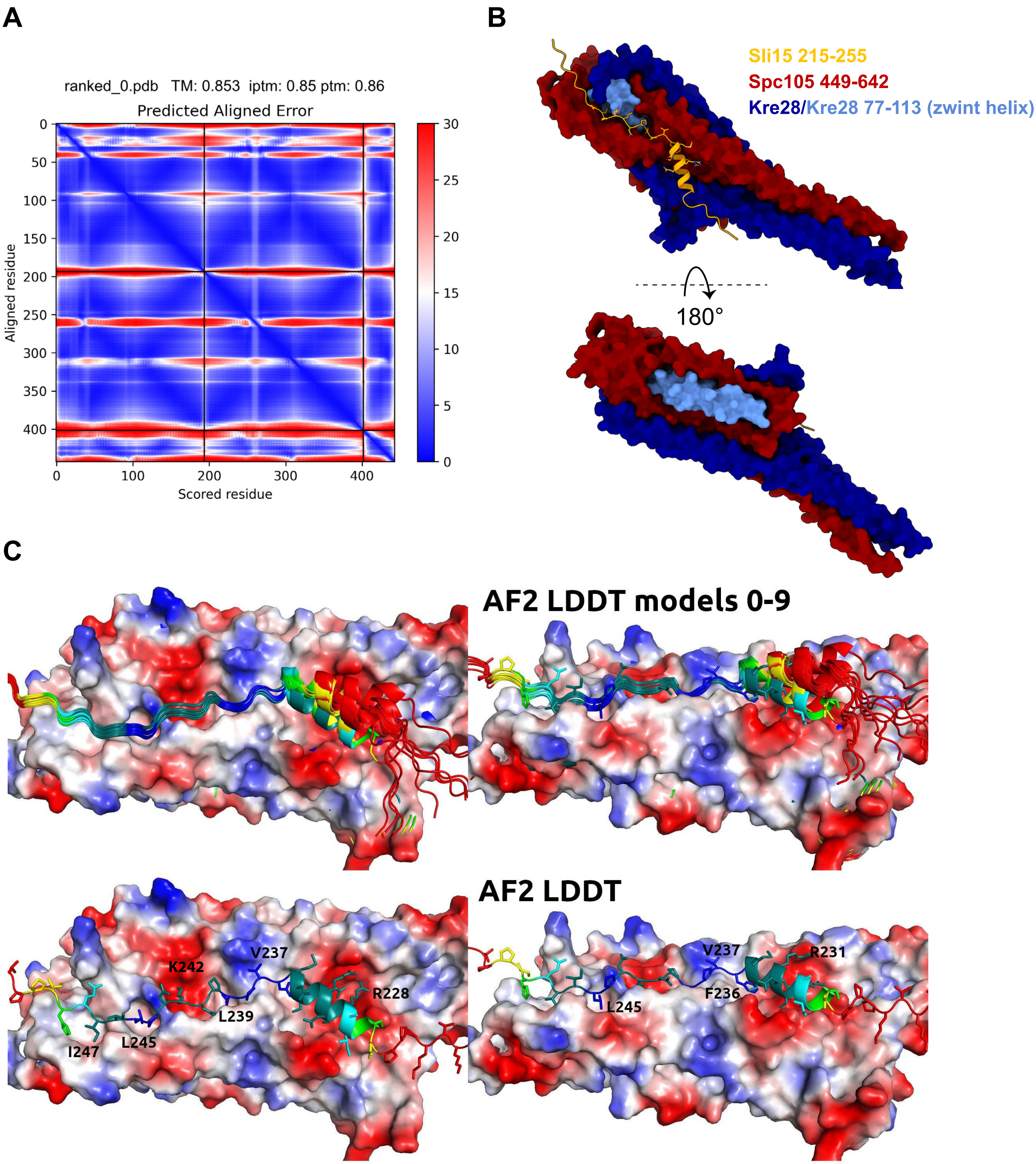
A
ranked_0.pdb TM: 0.853 iptm: 0.85 ptm: 0.86
Predicted Aligned Error
Aligned residue
Scored residue
B
Sli15 215-255
Spc105 449-642
Kre28/Kre28 77-113 (zwint helix)
180°
C
AF2 LDDT models 0-9
AF2 LDDT
K242
V237
R228
L239
I247
L245
V237
R231
F236
L245

 © The Author(s)

Figure EV4. Refers to Fig. 7.

(**A**) PAE plot of the top-ranked model of the Spc105 (449–642)-Kre28 (1–208)- Sli15 (215–255) complex. Fragment boundaries are indicated by black lines, blue are favorable scores, red are unfavorable. (**B**) Position of the Kre28 Zwint helix (light blue) in the top-ranked model. Note that only a part of the Zwint helix is involved in the binding interface with Sli15, while the majority of the helix is on the opposite side of the Spc105/Kre28 bundle. (**C**) Top row: Superposition of the ten top-ranked models of the Sli15 peptide on Spc105/Kre28. pLDDT scores for the Sli15 peptides are indicated with blue and green showing favorable, and red unfavorable scores. The Spc105/Kre28 surface is colored by electrostatic potential. Bottom: Details of the top-ranked model, with key side chains of the Sli15 peptide indicated as sticks. Coloring by pLDDT scores as above.

© The Author(s)

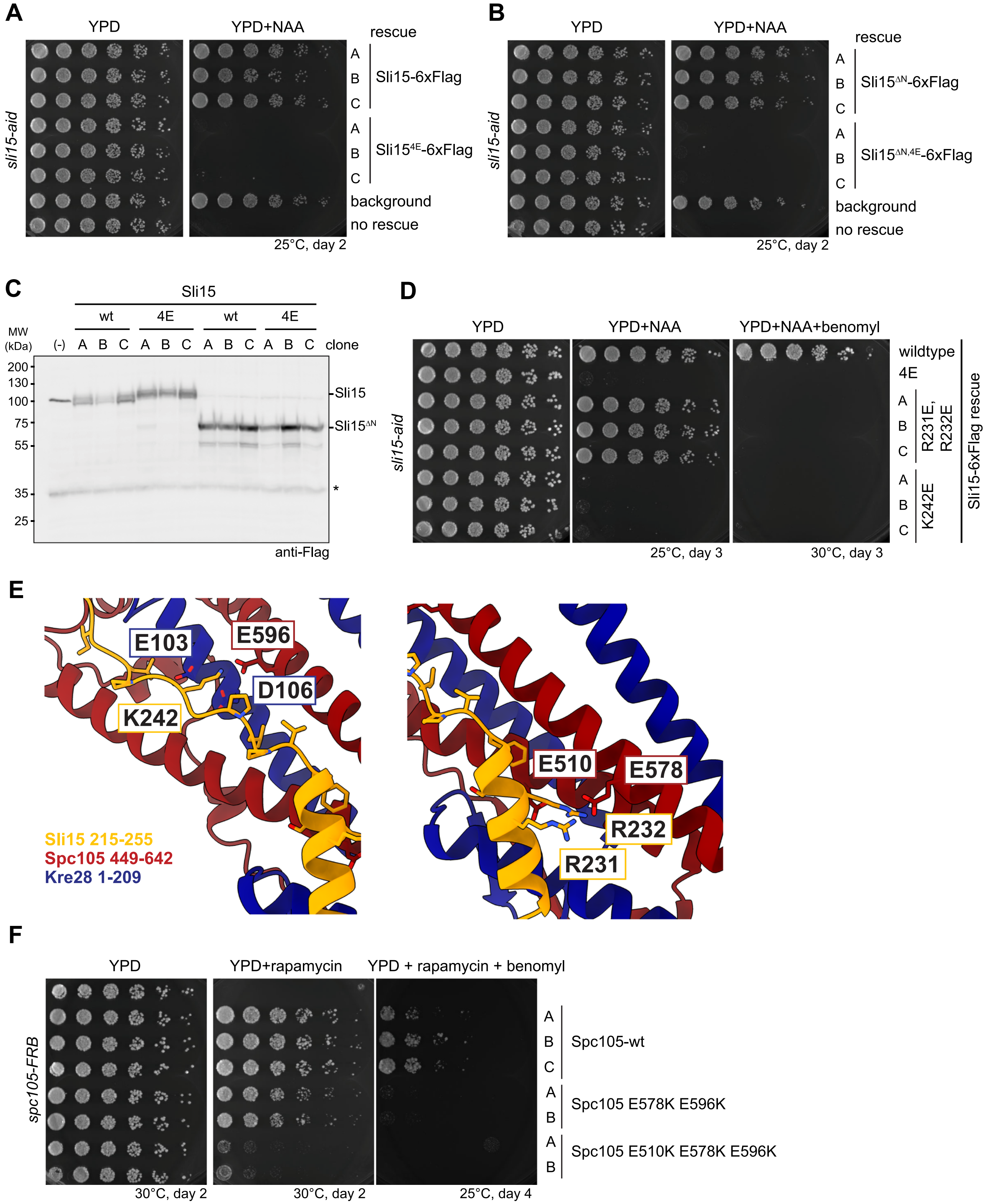
A
YPD
YPD+NAA
rescue
sli15-aid
A
B
C
Sli15-6xFlag
A
B
C
Sli15^4E-6xFlag
background
no rescue
25°C, day 2
B
YPD
YPD+NAA
rescue
sli15-aid
A
B
C
Sli15^ΔN-6xFlag
A
B
C
Sli15^ΔN,4E-6xFlag
background
no rescue
25°C, day 2
C
Sli15
wt
4E
wt
4E
MW (kDa)
(-) A B C A B C A B C A B C clone
200
130
100
75
55
35
25
Sli15
Sli15^ΔN
*
anti-Flag
D
YPD
YPD+NAA
YPD+NAA+benomyl
sli15-aid
wildtype
4E
A
B
C
R231E, R232E
A
B
C
K242E
Sli15-6xFlag rescue
25°C, day 3
30°C, day 3
E
E103
E596
D106
K242
Sli15 215-255
Spc105 449-642
Kre28 1-209
E510
E578
R232
R231
F
YPD
YPD+rapamycin
YPD + rapamycin + benomyl
spc105-FRB
A
B
C
Spc105-wt
A
B
Spc105 E578K E596K
A
B
Spc105 E510K E578K E596K
30°C, day 2
30°C, day 2
25°C, day 4

Figure EV5. Refers to Fig. 7.

(**A**) Serial dilution assay testing full-length Sli15 wild-type and -4E rescue constructs in a *sli15-aid* system. Three clones for wild type and mutant are tested. Background indicates a strain lacking *sli15-aid*, no rescue indicates a *sli15-aid* strain without a Sli15 rescue construct. (**B**) Serial dilution assay testing Sli15-ΔN wild-type and -4E rescue constructs in a *sli15-aid* system. Three clones for each version are tested. Background indicates a strain lacking sli15-aid, no rescue indicates a sli15-aid strain without a Sli15 rescue construct. (**C**) Western Blot analysis of Sli15-Flag rescue constructs tested in A and B. Asterisk denotes unspecific band. (**D**) Serial dilution assay testing the indicated Sli15 rescue constructs in a *sli15-aid* system. Three clones for each version (Sli15 R231E R232E, or Sli15 K242E) are tested. (**E**) Left side: Details of the predicted binding site surrounding Sli15 K242 (orange) with interacting residues in Kre28 (dark blue) or Spc105 (dark red) highlighted. Right side: Details of the predicted binding site surrounding Sli15 residues R231 and R232. (**F**) Serial dilution assay testing the effects of Spc105 mutations predicted to affect the Sli15-binding site. Spc105 rescue constructs are tested in an Spc105-FRB anchor-away system in which upon rapamycin addition the endogenous Spc105 protein is removed from the nucleus. Top row: control lacking a rescue construct. Three clones for wild-type rescue constructs, and two clones of each mutant are tested.
